# General intelligence disentangled via a generality metric for natural and artificial intelligence

**DOI:** 10.1038/s41598-021-01997-7

**Published:** 2021-11-24

**Authors:** José Hernández-Orallo, Bao Sheng Loe, Lucy Cheke, Fernando Martínez-Plumed, Seán Ó hÉigeartaigh

**Affiliations:** 1 VRAIN, Universitat Politècnica de València, Valencia, Spain; 2 Leverhulme Centre for the Future of Intelligence, University of Cambridge, Cambridge, UK; 3 Psychometrics Centre, University of Cambridge, Cambridge, UK; 4 Department of Psychology, University of Cambridge, Cambridge, UK; 5 Joint Research Centre, European Commission, Brussels, Belgium; 6 Centre for the Study of Existential Risk, University of Cambridge, Cambridge, UK

**Keywords:** Psychology, Computer science, Behavioural methods

## Abstract

Success in all sorts of situations is the most classical interpretation of general intelligence. Under limited resources, however, the capability of an agent must necessarily be limited too, and *generality* needs to be understood as comprehensive performance *up to a level of difficulty*. The degree of generality then refers to the way an agent’s capability is distributed as a function of task difficulty. This dissects the notion of general intelligence into two non-populational measures, *generality* and *capability*, which we apply to individuals and groups of humans, other animals and AI systems, on several cognitive and perceptual tests. Our results indicate that generality and capability can decouple at the individual level: very specialised agents can show high capability and vice versa. The metrics also decouple at the population level, and we rarely see diminishing returns in generality for those groups of high capability. We relate the individual measure of generality to traditional notions of general intelligence and cognitive efficiency in humans, collectives, non-human animals and machines. The choice of the difficulty function now plays a prominent role in this new conception of generality, which brings a quantitative tool for shedding light on long-standing questions about the evolution of general intelligence and the evaluation of progress in Artificial *General* Intelligence.

## Introduction

The intuitive idea of general intelligence is usually associated with “the capacity of getting along well in all sorts of situations”^1^, for a broad range of tasks^2–5^. In the real world, however, there are environmental pressures for specialisation, as ‘tasks’ are not equally likely, and not equally rewarded. But even for those tasks that are equally important, an agent with bounded resources (brain, energy, computation, etc.) would benefit from being *optimised* for the easy tasks. This cognitive pressure can happen through evolution in natural intelligence, be embedded by design in artificial intelligence (AI), or be learnt through development.

However, for more than a century there has been sustained evidence for something that could be reasonably called general intelligence in humans^6,7^, and some other animals^8,9^. There has also been a fundamental interest within the field of AI in building general intelligence, from the early general problem solver^10^, and McCarthy’s call for “generality in AI”^11^, to the recent goals of achieving progress towards Artificial General Intelligence (AGI). How can we reconcile this evidence with the environmental, especially cognitive, pressures that make agents more effective for a pocket of tasks?

First, we need to dissociate generality from capability, two notions that have been entangled under the term general intelligence. This entanglement has also been asymmetric. We are used to consider *savants*, specialists that are very capable for a specific set of tasks. However, can we conceive a general system that is not very intelligent, such as a generic assistant that can *only* do a wide range of simple things? Second, we need to reconsider the elimination of very easy and very difficult instances for evaluation. This practice is usually done for the sake of population variability—so finding that some agents behave better than others. Very easy instances are uninformative, because all the individuals solve them. So are very difficult instances, because no individual solves them. The range of difficulties is adjusted and narrowed for each test, so that there is sufficient variance to explain in the population^12^. Then, a *single* factor emerges explaining most of it, as first found by Spearman^6^. It was called the *g* factor, because it captures the populational variability of general performance in mental ability tests. But can we analyse generality without relying on the population variance? Third, we need to reformulate the hypothesis that generality may saturate at some high levels of capability. The traditional narrowing of difficulties to increase the variance has led to the controversial Spearman’s Law Of Diminishing Returns (SLODR), a hypothesis positing that *g* is manifested more strongly on less-able groups than more-able groups^6,13^. Can we re-examine this hypothesis once generality is decoupled from capability?

After all, the notions of general intelligence, and the *g* factor in particular, are populational. They are usually derived from the distribution of test results in humans, or some particular subgroups. Despite the use of species as populations, the extension of these notions to non-human animals has been problematic, because of small samples (of subjects and tasks), and some other methodological issues^8,9^. More critically, the notion of population is elusive for artificial agents, hybrids and collectives^14^. In the end, when we finally consider the question that some individuals can be more general than others, we then realise that, to date, no measure of *individual* generality has yet been introduced, different from the agent’s capability.

We must define generality independently from how other agents behave and how many instances we have of each domain or difficulty. This would solve the issue of relativity to a population and to a predefined partition of subdomains when comparing capability and generality. This would also help overhaul the analysis of benchmarks and competitions in AI, where no natural populations exist^15,16^. We would be able to detect when the agents specialise (overfit) for a subset of tasks, use tricks for pockets of problems^17,18^, and do not distribute their capability in a general way. Ultimately, we need a measure of generality in order to give a more objective definition of AGI (Artificial General Intelligence) This may pave the way for safer—yet general—systems in the future, by controlling capability through limited resources^19,20^.

The rationale behind such a new measure of generality is that, independently of its overall capability, *an agent can only be called fully general if it covers all tasks up to an equivalent level of difficulty, determined by the resources that are needed for them*. We derive this notion formally in the following sections and apply it to a range of tasks for humans, non-human animals and AI systems. It should be noted that this new measure of ‘generality’ does not replace the cognitive understanding of general intelligence, referred to as the existence of a broad mental capacity that influences performance on other kinds of cognitive tasks. Indeed, our endeavour deviates significantly from the “holey grail of general intelligence”, as criticised by Sternberg^21^ two decades ago. To really “provide the whole grail”, in Sternberg’s words, we have to break it into two.

The major contributions of this paper are the following: (1) The concept of general intelligence is disentangled through the introduction of two direct measures of generality and capability. (2) These measures are derived for individuals, and the values are independent from the population. (3) We prove theoretically and illustrate empirically that under conditions of limited cognitive resources and a range of equally likely problems, maximising expected positive outcome is benefitted by both high generality and high capability. (4) We show experimentally that diminishing returns in generality when capability is high do not appear for a range of subjects (humans, non-human animals and AI systems), and in a range of cognitive and perceptual tests. The first two contributions represent methodological and measurement innovations that can transform the way we evaluate capability and generality for individuals and populations. The second two contributions suggest an alternative, simple explanation for why general intelligence may appear at different points of evolution, and for why we should aim at generality in AI research. Overall, the metrics can help provide a quantitative, rather than qualitative, characterisation of Natural and Artificial General Intelligence.

## Methods

The first scientific notion of general intelligence for humans was introduced by Charles Spearman^6^. He observed that humans who performed well on some cognitive tests usually performed well on the others. More technically, the test correlations were positive, something that is known as a ‘positive manifold’. He derived the notion of the ‘*g* factor’, which is a latent factor that explained the variability in the matrix. However, this concept of the *g* factor is populational: the *g* factor does not provide a measure of the *generality* of an agent, but a feature of the population sample.

From this initial view of a dominant *g* factor explaining most of human cognitive performance, theories of intelligence have taken less extreme views, such as Carroll’s three-stratum theory^22^ or Sternberg’s triarchic theory of intelligence^23^. At the methodological level, psychometrics^24^, through classical test theory and item response theory (IRT), has been applied to test data, ranging from personality traits to cognitive abilities. In IRT, latent factors—including ability and difficulty—are estimated from items and respondents after the assumption of some parametric models and distributions. This ability in the IRT models is relative to the population used for the estimation and hence not properly comparable to other populations, even if evaluated on the same battery. While instance difficulty plays an important role in IRT, the distinction between ability and generality and the connection between generality and task difficulty have not been made explicit to date.

In animal cognition research, especially in evolutionary terms, general intelligence is also usually understood populationally (with intra- and inter-species factors, known as *g* and *G* respectively)^8,9^. From a methodological point of view, there are three important caveats when analysing general intelligence in non-human animals^25^. First, the samples are usually small, limiting the application of some techniques such as PCA or factor analysis. Second, the spectrum of tasks is narrow, not really covering a broad range of situations. Third, “variability in task difficulty” is limited “to avoid ceiling and floor effects” (all the agents failing or succeeding systematically for very easy or very hard tasks), to “allow for individual variation”^25^. However, animal cognition research can go beyond the psychometric approach to determine the pressures for general intelligence in the context of the evolution of nervous systems and the ecological niches of each species. General intelligence is usually explained as a response to cognitively-demanding niches. It is reasonable to assume that nervous systems evolve with resource constraints (see, e.g., the wiring economy principle^26^ in neuroscience, which postulates that neural networks are arranged in animal brains to minimise metabolic resource consumption). Given these limited resources and associated evolutionary effort, a species that has accommodated sufficient cognitive capability to succeed in (less demanding) scenarios *A* and *B* (but not more demanding *C*) is more likely to thrive than a species that succeeds in *C* (but not *A* nor *B*), assuming the three scenarios are equally likely in the species’s environment, and equally rewarded. Generality can thus be seen in the context of the trade-offs in cognition^27^ and the ecological amplitude between generalists (able to thrive in a wide variety of environmental conditions) and specialists (able to thrive only in a narrow range of environmental conditions). However, we lack a metric of generality placing an individual in this range between specialists and generalists.

Another different view of generality is based on the *transitivity* of performance, which is an important indicator in some AI competitions. For instance, in the general video game AI (GVGAI) competition, it has been found that “performance is non-transitive”^28^, meaning that “different algorithms perform best on different games”. In game theory, transitivity is easily understood in agent-vs-agent situations (e.g., chess): if $\pi _a$ beats $\pi _b$ and $\pi _b$ beats $\pi _c$ transitivity would imply $\pi _a$ beats $\pi _c$. In agent-vs-task situations, agent transitivity can be expressed as follows: if $\pi _b$ is more *capable* than $\pi _a$ and $\pi _a$ solves $\mu$ then $\pi _b$ should also solve $\mu$. That would suggest that $\pi _b$ *dominates* $\pi _a$. This transitivity is only likely to happen if there is a strong correlation between tasks (and hence a high *g* factor). But again, this property (which by definition involves two agents) is understood for a *population* of agents: an agent $\pi _b$ may be very transitive for a population of agents $P_1$ but not for another population $P_2$.

Finally, there is a *potential* view of general intelligence, as the capability of learning any skills and acquiring knowledge in a diversity of domains. In order to evaluate this potentiality, tasks must be defined accordingly, under controlled learning situations and developmental settings. However, a broad battery of tasks can also be devised to evaluate some consolidated skills or knowledge, independently of how these have been acquired. In these conditions, we would explore *actual* capability and generality instead. The metrics we present here are applicable for both potential and actual scenarios, but for the sake of clarity, the experiments in this paper only include consolidated (i.e., actual) performance. The developmental setting presents some extra complexities that we will specifically cover in the discussion, at the end of the paper.

### Beyond domain-dependent variance

**Figure 1:**
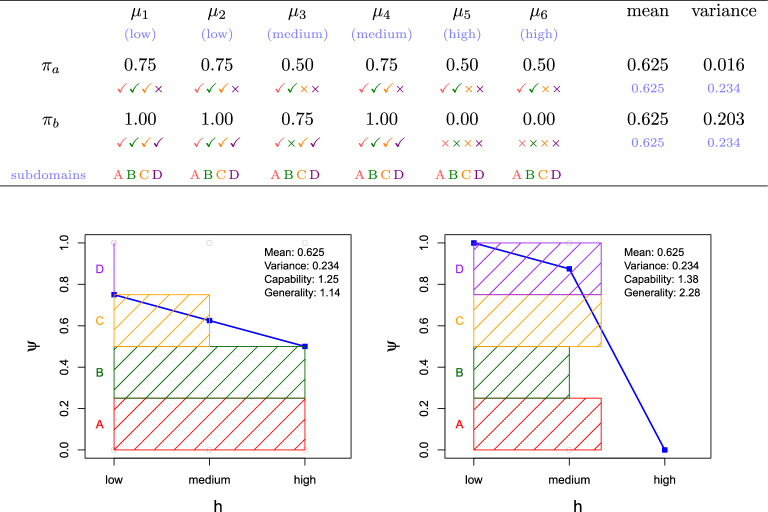
The role of difficulty when Agent Characteristic Curves (ACCs) are derived from a result matrix, and the new metrics represented in the spread vs capacity plot. Top: Response matrix *R* with cells $r _{j,i}$, where rows represent the agents (also referred to as respondents or subjects) and columns represent the tasks (also referred to as items or instances, depending on the aggregation). The difficulty of each task (low, medium or high) is shown in parentheses just below the name of the task. While the subdomains may not be known, if we cluster some questions in a particular way (A B C D), we see that one agent ($\pi _a$) completely neglects subdomain D. Bottom: We show the responses of these two different agents corresponding in terms of difficulty (*h*) on the $x$-axis. Left: $\pi _a$. Right: $\pi _b$. We also highlight how the two agents fare for the four domains in which the items are grouped. Having the same average result (0.625), which agent, $\pi _a$ or $\pi _b$, is more general?.

Consider the example in Fig. 1 (top). The values in black represent the results of two agents $\pi _a$ and $\pi _b$, and six tasks, $\mu _1$ to $\mu _6$. If we just look at their average scores (row-wise means), these two agents perform equally (0.625). In order to identify an *individual* measure of generality we could look at the variances of results for each particular individual (0.016 and 0.203). Under this view, $\pi _a$ could be considered more general, as it gets some score on all the tasks, while $\pi _b$ fails completely at two of them. However, this interpretation relies on three assumptions. First, variance makes sense only if the magnitudes are commensurate. This is almost never the case. Why is 0.75 in task $\mu _2$ commensurate to 0.75 in task $\mu _4$? Second, $\mu _1$ to $\mu _6$ seem to represent six different subdomains. But if the tasks are grouped into different domains, the interpretation could be completely different. Third, and not always fully recognised, tasks are assumed to have similar difficulty. Difficulties are chosen to have informative tests. Otherwise, very easy or very difficult questions would get the same uninformative result from all individuals. However, in uncontrolled situations, (1) tasks may be of a wide range of difficulties, leading to high row variances, and (2) specialisations are not detected if they appear beyond the assumed *subdomains* used to group the tasks.

Let us pay attention to the coloured symbols in the matrix of Fig. 1. We see that each task is composed of four binary items, where success or failure is represented by $\checkmark$ and $\times$ respectively. We also consider a possible assignment of the $6\times 4=24$ items to four domains: A, B, C and D. Now, the variance for the binary items is simply $p(1-p)$ with *p* being the mean of the Bernoulli distribution, equal (0.2349) for both $\pi _a$ and $\pi _b$. The way items are grouped would determine this naive view of generality.

Alternatively, assume we know the difficulties of the items of each task, as shown on the header of the matrix in Fig. 1 (low, medium or high). For easy items, $\pi _b$ is consistently good, with performance sharply falling for more difficult items. However, $\pi _a$ is similarly inconsistent at all difficult levels (in fact, $\pi _a$ is always wrong at problems of domain D). Figure 1 (bottom plots) shows a graphical representation of the 16 items, situated by their difficulty on the $x$-axis, and the aggregated value per difficulty as a blue curve. By splitting the covered area in four regions, we see how this aggregation is achieved with the different subdomains at different difficulties. We now see more clearly how agent $\pi _a$ (left) neglects subdomain *D*.

With this example, we see that the panorama changes completely from considering generality as low row-wise variability (with spurious results depending on the grouping and the difficulties in each domain) to instead considering *generality as solving the range of tasks up to a given task difficulty*.

The starting point for a new understanding of generality, as illustrated on the $x$-axis in Fig. 1 (bottom plots), is a *difficulty metric*, denoted by $\hbar$. In the previous example, difficulties were ordinal (low, medium, high), but we will generally assume that the difficulty metric assigns a real number to each instance. Difficulties can be derived *intrinsically* from the properties of the instance (e.g., size, number of components, noise, distortions, etc.) or the resources that are expected to solve it (e.g., working memory, primitives, etc.). They can also be derived *extrinsically* from the results of other agents (e.g., easy instances are those that are solved by most agents in a population).

### Agent characteristic curves and capability

**Figure 2:**
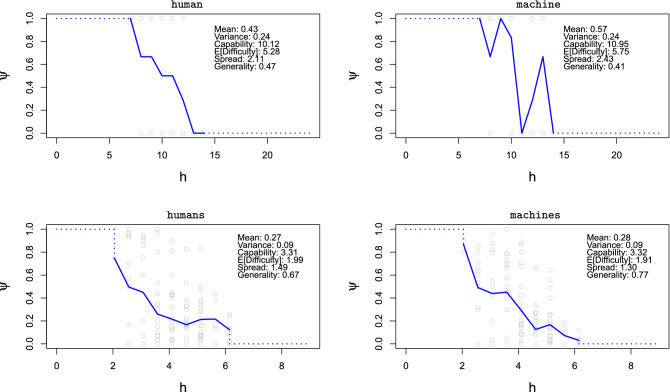
Agent characteristic curves (ACC), showing agent responses in terms of difficulty (*h*). The responses are shown as grey circles. The means of these responses for each difficulty are shown in blue, and connected to form an ACC. The plot shows the values for mean performance and its variance (Bernoulli: $p(1-p)$, when responses are binary), capability (${\Psi } _j$), expected difficulty (${{\mathbb {H}}}_j$), spread ($S _j$) and its reciprocal, generality (${\mathit{\Gamma}}_j$). The more compact the curve is, like a single steep fall, the higher the generality. Note that generality cannot be calculated from curve slopes. Top: a human (left, subject #22) and a machine (right, ‘repdiff’), both on 25 instances of the letter series problems as described in the text. The human has higher generality and lower capability than the machine. Bottom: an aggregation of human population (left) and an aggregation of Q-learning agents (right), both for 140 experiments from a benchmark comparing humans and simple reinforcement learning algorithms^29^. With similar capability, machines are more general.

If we represent the accomplishment (response) of an agent (respondent) over a range of difficulties, the points form an agent characteristic ‘curve’ (ACC). If the agent performs worse as difficulty increases, we typically find decreasing curves, as the two cases in Fig. 1 (bottom plots, curves in blue). For smooth sigmoidal curves, capability could be related to the position where the curve drops (the position on the $x$-axis with highest slope) and generality could be seen as the slope of the curve. In the extreme case, where the results are consistently 1 up to a certain difficulty level and 0 beyond it, we would have a perfect step curve, and generality would be maximum. Figure 2 (top) shows two real ACCs for a letter series problem, including several metrics. We see that the human (left) presents a smoother curve than the machine (right). Actually, the ‘curve’ for the machine is so saw-like that we cannot really compare its slope with the human’s.

The bottom plots in Fig. 2 show a comparison between humans and machines for a different benchmark, a test based on a reinforcement learning environment from first principles^29^. The larger number of items and the fact that we are comparing populations (and repetitions) rather than individuals, makes curves smoother. For this particular set of items, machines are more general than humans.

As we see in these examples, ACCs are rarely smooth and sigmoidal. Basing capability and generality on maximum slope and its position would require model estimates that may not fit the curve well. Instead, we simply define capability as the area under the curve, which differs from this position in cases that deviate from a pure sigmoidal curve. It also differs from average performance; capability would be linearly related to average performance only if the same number of instances existed for each difficulty at regular intervals.

Let us see the definition of capability formally. By $\psi _j(h)$ we denote the average accomplishment or performance of an agent *j* on all the problems of difficulty *h*. We assume performance is dimensionless either because it is a proportion (% correct) or it is normalised by maximum theoretical performance. Then, an agent characteristic curve (ACC) is simply a plot of $\psi _j(h)$ as a function of *h*. Capability is just the area of this curve:

1 $$\begin{aligned} {\Psi } _j \,{\mathop {=}\limits ^{\text {def}}}\,\int _0^\infty \psi _j(h)\, dh \end{aligned}$$

Because $\psi _j(h)$ is dimensionless, ${\Psi } _j$, as an integral over difficulties, has the same units as difficulty. If task difficulties on the $x$-axis are discrete, originally or by binning, and do not form a monotonous curve at all (e.g., Fig. 2 top right), the capability could still be calculated as the area by a trapezoidal method.

### Spread and (normalised) generality

In order to introduce a measure of generality that captures systematic performance for all problems *of low difficulty*, we must look at how *compacted* the curve is on the left—how *steplike* it is. We can quantify how much mass deviates from the left with the first partial moment of the ACC for agent *j*, denoted by $M _j$:

2 $$\begin{aligned} M _j \,{\mathop {=}\limits ^{\text {def}}}\,\int _0^\infty h \cdot \psi _j(h)\, dh \end{aligned}$$

Since $\psi _j$ is not a density function, $M_j$ is not the moment of a probability distribution. We can normalise this moment (dividing by capability) and get the expected difficulty for agent *j* for the successful items (binned by difficulty):

3 $$\begin{aligned} {{\mathbb {H}}}_j \,{\mathop {=}\limits ^{\text {def}}}\,{{\mathbb {E}}}_{h\sim f_j}[h] = \frac{ M _j}{{\Psi } _j} \end{aligned}$$

where $f_j(x) = \psi _j(x) / \Psi _j$. In other words, ${{\mathbb {H}}}_j$ is the expected difficulty conditioned to accomplishment, i.e., average difficulty for all successful item responses. Now, for a distribution that is fully compacted on the left (a single step function), ${{\mathbb {H}}}_j$ should be *half* of the capability. This means that twice the expected difficulty minus capability should be zero. We multiply this difference by capability (to get the moment back again), so that it is independent of location. Finally, it is square rooted, so that the final unit is commensurate with difficulty. The result is known as *spread*:

4 $$\begin{aligned} S _j \,{\mathop {=}\limits ^{\text {def}}}\,\sqrt{\left( 2{{\mathbb {H}}}_j - {\Psi } _j \right) \cdot {\Psi } _j} = \sqrt{2 M _j - {\Psi } _j^2} \end{aligned}$$

If we understand capability as the location on the $x$-axis of an idealised steplike curve, spread would be interpreted as measuring how much (and how far) the mass spreads over the left and right of that location.

Finally, we simply define generality as the reciprocal of spread, i.e.:

5 $$\begin{aligned} {\mathit{\Gamma}} _j \,{\mathop {=}\limits ^{\text {def}}}\,\frac{1}{ S _j} \end{aligned}$$

Note that ${\mathit{\Gamma}} _j$ is just a metric that can be applied to any possible set of points or any function $\psi _j$ mapping difficulties to accomplishment, provided that the difficulty function leads to a finite area under the curve. No parametric model is assumed.

Given these definitions we can better understand what kind of metrics generality and capability are by enumerating these eight properties (§E.2 gives more detail about them):

1. Translation: any positive translation of the points of the curve by *k* units of difficulty implies that capability becomes ${\Psi } _j + k$.
2. Compactness: with equal capability (area under the ACC), any redistribution of the mass to the left (keeping the area constant) increases generality.
3. Maximum generality (${\mathit{\Gamma}} _j=\infty$) and minimum spread: given a fixed capability ${\Psi } _j$, the minimum expected difficulty ${{\mathbb {H}}}_j$ and the maximum generality ${\mathit{\Gamma}} _j$ are obtained with an ACC as a decreasing step on $h={\Psi } _j$.
4. Constant interval: given a constant function with $y=c$ from difficulties 0 to *q*, we have ${\Psi } _j = cq$ and $S _j = q\sqrt{c(1-c)} = \sqrt{{\Psi } _j(q-{\Psi } _j)}$.
5. Minimum generality: given a fixed capability ${\Psi } _j$, the minimum generality ${\mathit{\Gamma}} _j$ for difficulties bounded by *q* is obtained with an ACC as an increasing step on $h=q-{\Psi } _j$, leading to $S _j = \sqrt{2{\Psi } _j(q-{\Psi } _j)}$.
6. Task transitivity: if a completely general agent solves a task then it also solves any other easier task.
7. Agent transitivity: if two agents $\pi _a$ and $\pi _b$ have maximum generality, and agent $\pi _a$ is at least equal to $\pi _b$ in capability, then for every task $\mu$ that $\pi _b$ solves then $\pi _a$ also solves $\mu$.
8. Same units: capability and spread are measured in the same units as task difficulty.

These properties show that (#1) we can increase capability without losing generality, and (#2 and #3) maximum (infinite) generality is obtained when the curve *compacts* all the mass on the left (on the easy instances). This suggests an optimal way of achieving a given capability. When generality is high we have that agent and task transitivity hold (#6 and #7). This means that general agents are more predictable about what they can and cannot do, provided we have a notion of the difficulty of a problem. Considering difficulty going from 0 to *q*, properties #4 and #5 show that squared spreads span in a band that goes from $S ^2_{min}=0$ to $S ^2_{max} = 2{\Psi } _j(q - {\Psi } _j)$, with $S ^2_{cnst}= {\Psi } _j(q - {\Psi } _j)$ in the middle.

**Figure 3:**
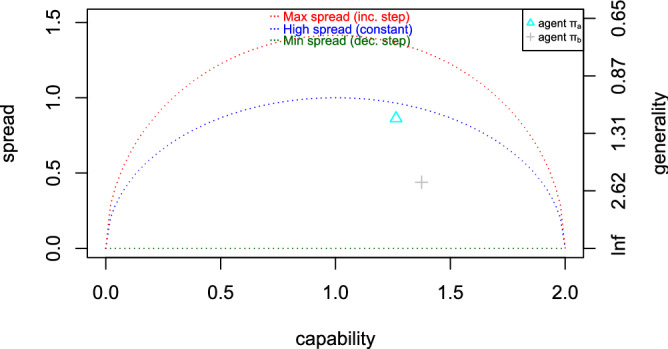
Representation of the two individuals in Fig. 1 in the space of spread versus capability. As spread is reciprocal to absolute generality, the lower the point the more general. The isometrics (dotted lines) represent the maximum normalised generality ($\gamma =1$, flat at the bottom, in green), the isometrics for a constant ACC ($\gamma =0$, in blue) and the isometrics for minimum normalised generality ($\gamma =-1$, top, in red).

These three cases are seen in Fig. 3 for the two individuals in Fig. 1, as the ‘isometrics’ for maximum generality (flat at the bottom, in green), the isometrics for a constant ACC (in blue) and the isometrics for minimum generality (top, in red). These three singular cases suggest the following normalisation of spread:

6 $$\begin{aligned} {s}&\,{\mathop {=}\limits ^{\text {def}}}\,&{\sqrt{\frac{ S ^2 - S ^2_{cnst}}{ S ^2_{max} - S ^2_{cnst}}}} \end{aligned}$$

Again, this normalised spread increases as generality decreases. Finally, we can simply define *normalised generality* as $\gamma \,{\mathop {=}\limits ^{\text {def}}}\,-s$, going from $-1$ (minimum generality) to $+1$ (maximum generality).

The isometrics in Fig. 3 now correspond to maximum normalised generality ($\gamma =1$, in green), a constant ACC ($\gamma =0$, in blue) and minimum normalised generality ($\gamma =-1$, in red). Normalised generality becomes higher the further down from the constant isometric in blue. Note that an agent that is randomly correct in a given percentage of instances that is independent of difficulty would typically have $\gamma =0$, and would be located somewhere on the constant isometric. If it behaves better for easier problems, then it would fall below this blue isometric. So whenever we see most or many of the points below the constant isometric, we can say that the population shows some generality. We will use this normalised generality measure when we want to compare situations with different difficulty scales. This normalisation also allows for a more relative appraisal of generality when it is close to the edges of the considered range of difficulties. More details in §F.5.

## Results

In this section we apply what we call a *generality analysis* (GA) to various agents and tasks. In all cases, we start with a response matrix $R_{M \times N} \in (0,1)$, i.e., a matrix of *M* rows (agents) and *N* columns (items). The first five subsections work with an intrinsic measure of difficulty $\hbar$. The final subsection addresses other situations where we need some transformations or the derivation of a metric of difficulty before performing GA. In addition to illustrating how the metrics can be used to characterise individuals and summarise populations, we analyse several questions: (1) whether the metrics of capability and generality decouple for individuals and groups, (2) whether the results are complementary to those obtained with other techniques such as PCA, factor analysis and IRT, when applicable, and, most importantly, (3) whether the articulation of SLODR in terms of the new metrics holds or not for each of the studied domains.

### Elithorn’s mazes

Elithorn’s Perceptual Mazes^30^ were introduced to study frontal lobe functions, and since then the test has been used mainly to study human problem-solving skills. Previous research has shown that this maze test is highly correlated with several intelligence tests^31^.

A maze shows a lattice with some yellow dots that have to be collected (see Figure S1 in §A.1 ). A subject is successful if her route collects the maximum number of yellow dots. Given the structural nature of the task, several parameters of the maze have been considered to influence task difficulty: the size of the maze, the density of yellow dots and the number of steps needed^32^. We administered 23 test items with a range of difficulties (from 8.88 to 19.41) to 496 participants.

**Figure 4:**
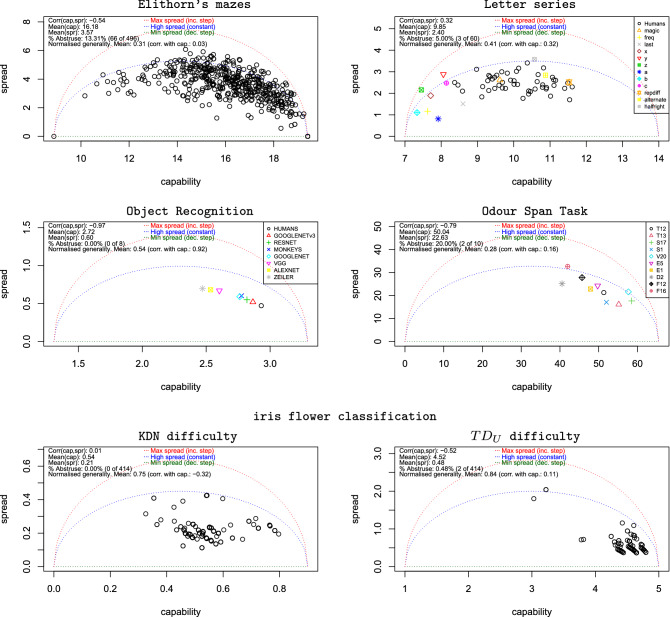
Capability vs spread for the intrinsic difficulties scenarios. The space is characterised by three normalised generality isometrics shown as dotted curves: the maximum spread in red (complete abstrusity), the minimum spread in green (complete generality) and a middle case where results are independent of difficulty (constant ACC). Top left: Elithorn’s mazes. Results for all 496 participants. Top right: letter series for all participants (48 humans and 12 machines). Middle left: object recognition scenario using the intrinsic difficulty derived from the psychophysical parameters). Middle right 10 rats in the Odour Span Task, with difficulty being the number of scents to remember. Bottom: iris classification problem using 419 classifiers from study 7306 in OpenML (after classifiers with accuracy below 0.35 removed). Left, capability vs spread using KDN difficulty ($k=10$). Right: using $TD_U$ difficulty instead.

Figure 4 (top left) shows very different results for both capability and spread, with points ranging relatively freely around and below the blue isometric. The points usually get closer to the red and the green isometric curves the further we go to the right, but we have to see this in terms of the normalised values, as the three isometrics collapse at the extremes. Of course a very capable agent will have very few errors, mostly occurring in the most difficult cases (although some will occur in the easy problems through mistakes). Overall, the mean normalised generality is 0.31, with only 13.31% of ‘abstruse’ subjects (i.e., with normalised generality lower than 0, meaning that they fail more on the easy items). Given the number of task items per individual (23), abstruse agents may follow specialised strategies that work for some subdomains of the problem (e.g., those subjects that solve mazes in a greedy way, or those who prefer to stay in the middle, etc.) but not for others.

The supplementary material (Fig. S3 in §A.1) gives detailed results by subpopulations (gender, education and age). For gender, males are slightly more general (generality changes from 0.29 to 0.32), but the percentage of abstruse individuals is higher for males too (from 11.79% to 14.29%). For education, generality remains almost constant and for age we find that young people are usually more general. We also perform factor analysis, PCA and IRT on this data, and we see that no single factor can explain more than 0.123 of the variance, which means that there is no dominant (general) factor. Note that factor analysis does not allow us to compare the generality of individuals. In contrast, even if no dominant factor is found and average normalised generality is also low (0.31), Fig. 4 (top left) shows that *some* individuals are very general.

Finally, we can analyse whether we observe Spearman’s Law of Diminishing Returns (SLODR). Fig. 4 (top left) shows that the correlation between capability and normalised generality is 0.03. More capability does not imply lower generality (no SLODR). This is confirmed by the two plots at the bottom of Fig. S3 in the supplementary material, splitting subjects into a low-capability group and a high-capability group (using the median as split point).

### Letter series

Let us now consider another test that has been used in the analysis of general intelligence: the letter series. These series were initially introduced by Thurstone in his Primary Mental Abilities theory^33^ in the 1930s and revisited by Simon and Kotovsky in the 1960s to analyse questions about specialisation and difficulty^34^. Also, the letter series has been a popular challenge for computer models^35^.

Instead of the original Thurstone’s sequences, we take 20 prediction and 15 imputation instances, generated in such a way that we have an information-theoretic metric of difficulty (Levin’s Kt). We reuse the results from the C-test^36^ for 48 humans and add the results of 12 artificial systems (see Table S3). More details in §A.2.

On the top right plot of Fig. 4 we see that humans (black circles) usually have higher generality than many of the ad hoc computer solutions (which are usually nearer the blue isometric). Systems that output the same letter (x, y, z, a, b or c) regardless of the series appear on the extreme left of the plot, with low generalities (the only exception is a, but this is caused by a few easy problems having a as solution). A similar thing happens with last. We see that all these naive systems follow the pattern of Fig. 1 (bottom left), where some subdomains are neglected. More capable systems include magic (MagicHaskeller^37^), an inductive programming system not specifically designed for letter series that appears quite central with respect to humans in terms of capability and generality. The two systems based on arithmetic progressions (repdiff and alternate) perform really well, with capability scores of between 11 and 12. However, their generality is lower than most humans, which reveals that these systems are able to solve some difficult series that follow this kind of pattern but not other series that follow different, but simple, patterns. To a lesser extent than the other naive machine models, they are also neglecting some subdomains.

If we analyse the results of the 48 humans only (split in Fig. S11 in §A.2), we see a normalised generality of 0.45, which is quite relevant taking into account that the difficulty has been derived purely from a computational variant of Kolmogorov complexity (Levin’s Kt), being very informative of whether a problem is easy or hard for humans. Indeed, Levin’s Kt has been postulated as a universal metric of difficulty^14^. Also interesting is the correlation between capability and normalised generality, which is 0.36. Again in this case we do not observe any SLODR phenomenon (on the contrary, more capable agents are usually more general).

### Object recognition

Rajalingham et al.^38^ used millions of trials were used to compare the behaviour between primates (humans and rhesus macaques) and deep convolutional artificial neural networks (ANNs) for object classification tasks. The authors of this study found that ANN architectures “significantly diverged from primate behavior”. Interestingly, the study used psychophysical distortions of the images to create variants of the objects (and hence task items), which we can use here to derive an intrinsic metric of difficulty. Hence, we can analyse how ANNs fare against primates in terms of capability and generality.

We first explain the object recognition task. Given the original image of an object (e.g., zebra), not shown, one distorted version is shown to the subject for 100 ms. After removal of this image, two choice images are shown to the left and right (e.g., the original non-distorted zebra image vs a non-distorted dromedary) and the participant has to choose which image corresponds to the object shown initially. This setting was the same for humans, macaques and six ANN architectures. The description of the 240 results for each of the eight agent groups and the way the intrinsic difficulty was derived from four psychophysical attributes can be found in §A.3, as well as the ACCs for the eight groups (Fig. S15).

Figure 4 (middle left) shows a very high correlation between normalised generality and capability (0.92). Humans have the highest generality, followed by GOOGLENETv3, and the same is true for capability. The data provided by the experimental setting has some limitations for the analysis of generality. First, we only have a small range of difficulties for the $x$-axis (see ACCs in Fig. S15). Second, we are not using binary responses but the results for each item is an aggregate, with two different population sizes (1472 anonymous humans and 5 macaque monkeys). Because of this we cannot really say much about SLODR in this case, as higher capabilities here would usually be followed by a steep completion of the curve, leading to high generality as we see for humans. The experimental setting on its own might be causing the high correlation between normalised generality and capability (0.92). Nevertheless, in Fig. S15 we see that humans and monkeys are consistently better at the problems of very low difficulty ($\leqslant 2$), where performance is always greater than 0.6. This does not hold for any of the ANN architectures.

### Odour span task

The Odour Span Task (OST) is a very common task to detect changes in olfactory working memory, for different species, genetic variations or drug effects^39^. The test is taken to an unprecedented number of stimuli (72 odours) by April et al.^40^, suggesting that OST may not be measuring the same processes as in human working memory.

This task has some interesting characteristics for its study under the prism of generality. First, difficulty depends on the number of odours to remember as it is related to memory resources (independently of how this memory is implemented physiologically). Second, although accuracy can be used to analyse the performance of rats, it is more common to use a metric known as “span length”, which is defined as the number of correct responses in a row (minus one), starting from the beginning. The metric is assuming a steplike curve of performance (§A.4).

We have processed the results of experiment 2 by April et al.^40^, focusing on odour memory capacity for 10 rats, where for each trial the rat must tell between an odour previously presented and the new odour. This configuration is basically a new-vs-old task, but it still depends on remembering up to 72 different odours seen before. This experiment was performed in sessions of 36, 48 and 72 stimuli. We have simply averaged the results for the 36, 48 and 72 trial cases to have more stable data and then we have binned the number of stimuli to remember in groups of 10. We use trials up to 71 (binning in groups of ten: 2–11, 12–21, ...62–71), with the mean number of previously seen scents for each bin (5.5, 15.5, ...) as difficulty. The capability and spread are shown in Fig. 4 (middle right).

We interpret the results as follows. Generality is usually low, as most rats behave very close to a constant response curve (dashed blue). This seems to be aligned with the conclusions by April et al.^40^. Here, generality would represent how much the rat complies with the OST hypothesis of memory load acting as a general factor behind the results. As generality is low for most rats, they may be very good for some instances and very bad for some others of the same difficulty. This means that given their capability and the difficulty of the trial (number of odours), we cannot really predict whether the rat is going to succeed or not. More details in §A.4.

### Feature-based classification

We now explore how the capability and generality metrics can be applied to supervised problems, such as classification, a very common setting in machine learning. We have selected ‘iris’, an archetypal dataset in statistics and machine learning introduced by Ronald Fisher in 1936. The dataset has 150 instances of flowers to be classified into three different species (Iris setosa, virginica and versicolor) using four features: the length and the width of the sepals and petals. In our study, we use 419 classifiers from OpenML.

For the choice of intrinsic difficulty, we have a wide variety of options to choose from, using the so-called ‘instance hardness metrics’^41,42^. We will start with KDN, the *k*-disagreeing neighbours, sometimes also known as separability. Intuitively, given an instance, it uses a distance or metric of similarity to find the $k=10$ closest instances, and difficulty is simply derived as the proportion that are of a different class.

Figure 4 (bottom) includes a series of plots. The left plot shows the distribution of capability vs spread for all classifiers. We see no abstruse or very specialised classifiers (above the blue isometric). Mean normalised generality is quite high (0.75). The correlation between normalised generality and capability is now negative. This is explained by a U-shape distribution of the whole set of points, which is quite unusual compared to other scenarios seen in previous subsections.

We also explore another difficulty metric, Tree Depth for Unpruned tree ($TD_U$), also taken from Smith et al.^41^. $TD_U$ measures the depth of the leaf node that classifies an instance (so more conditions are needed to disambiguate it), an estimate of the minimum description length for an instance. In this case, the classifiers are much more compacted on the bottom right corner of the capability vs spread plot Fig. 4 (bottom right).

It is insightful to single out some of the points in the plot and identify the classification techniques behind them. For instance, for metric KDN, the classifier with highest capability (point $\langle 0.797, 0.194 \rangle$) is a neural network, while the one with highest generality (lowest spread, point $\langle 0.525, 0.113\rangle$) is a NBTree (a decision tree using Naive Bayes assignment at the leaves). For metric $TD_U$, the classifier with highest capability (point $\langle 4.80, 0.379\rangle$) is a linear discriminant, while the one with highest generality (point $\langle 4.65, 0.368\rangle$) is a support vector machine. §A.5 shows the curves for these classifiers (Fig. S18) and identifies more classifiers at the extreme values of capability and generality.

### Scenarios with extrinsic difficulties

The examples in the previous subsections start from an intrinsic metric of difficulty, one that derives from the characteristics of the problem. Can we derive generality and capability measures from the results over a set of instances without an intrinsic difficulty? The answer is positive, but there is more than one way to do this, usually involving a transformation of the data, depending on the situation.

Table 1 summarises different types of modalities or transformations leading to generality analysis. We will refer to them by their acronym, derived from the type of approach (‘Approach’ column). The table starts with GA, which requires a response matrix and a metric of difficulty ($\hbar$). All the others take the response matrix and generate an extrinsic measure of difficulty (except for the last one, which is just factor analysis and, as the similar PCA, would not generate difficulties). As we see, some cases do not need to transform the response matrix $R_{M \times N}$, such as Opp and IRT; the output has the same size and type as the original response matrix. In the other three cases, either we have a discretisation into (0,1) values (using the results of a reference agent as a threshold), as in the ARef case, or we have a more drastic change where many new columns are created (*c* for each original column), by going through *c* values and determining whether the cell is above that value. IRT can be applied whenever the response matrix is binary while FA takes continuous values, but in both cases there are strong constraints on the minimal sample size to reach a meaningful result. Note that GA and DRef can be applied to a single individual, and these are the only two cases that avoid population effects. Nevertheless, one can always do the Opp, Aref, Rnk and IRT transformations with a response matrix from one population, and then apply the difficulties to a fresh individual out of that distribution. We see that IRT and FA usually require a large number of items (*M*), but the other transformations do not, which is another advantage of GA (with or without the transformations).

**Table 1:** Several modalities for applying generality analysis depending on various situations and transformations. We show the input (size and types of the response matrix *R* and some other information), the minimum sizes (approximate rules of thumb in some cases or statistical analysis in others^43^), a short description of the approach and the output. The top row (case GA) is the canonical case, as it starts with a difficulty function $\hbar$. All the others (except the last one, included for completeness, and similar for PCA) derive the difficulty function (which jointly with the response matrix can be used as input for the first case, the GA).

Case	Input (agents $\times$ items)	*M*	*N*	Approach	Output
GA	$R_{M \times N} \in (0,1)$, $\hbar$	$\ge 1$	$\ge 5$	Generality Analysis	${\Psi }$ and ${\varGamma }$
Opp	$R_{M \times M} \in (0,1)$ (matches)	$\ge 5$	–	$\hbar \leftarrow$ Opponent’s Avg. Perf.	$R_{M \times M} \in (0,1)$, $\hbar$
ARef	$R_{M \times N} \in {\mathbb {R}}$, *RefAgent*	$\ge 20$	$\ge 20$	$\hbar \leftarrow$ % worse than *RefAgent*	$R_{M \times N}^{\geqslant RefAgent} \in \{0,1\}$, $\hbar$
Rnk	$R_{M \times N} \in {\mathbb {R}}$, *c*	$\ge 20$	$\ge 5$	$\hbar \leftarrow$ *c*-ranks	$R_{M \times cN}^{\geqslant Rank} \in \{0,1\}$, $\hbar$
DRef	$R_{M \times N} \in {\mathbb {R}}$, *RefDist*, *c*	$\ge 1$	$\ge 5$	$\hbar \leftarrow$ *c*-quantiles of *RefDist*	$R_{M \times cN}^{\geqslant Quantile} \in \{0,1\}$, $\hbar$
IRT	$R_{M \times N} \in \{0,1\}$	$\gg 200$	$\ge 20$	$\hbar \leftarrow$ IRT difficulty	$R_{M \times N} \in \{0,1\}$, $\hbar$
FA	$R_{M \times N} \in {\mathbb {R}}$	$\geqslant 10N$	$\ge 5$	Factor analysis	Loadings on factors (*g*)

**Figure 5:**
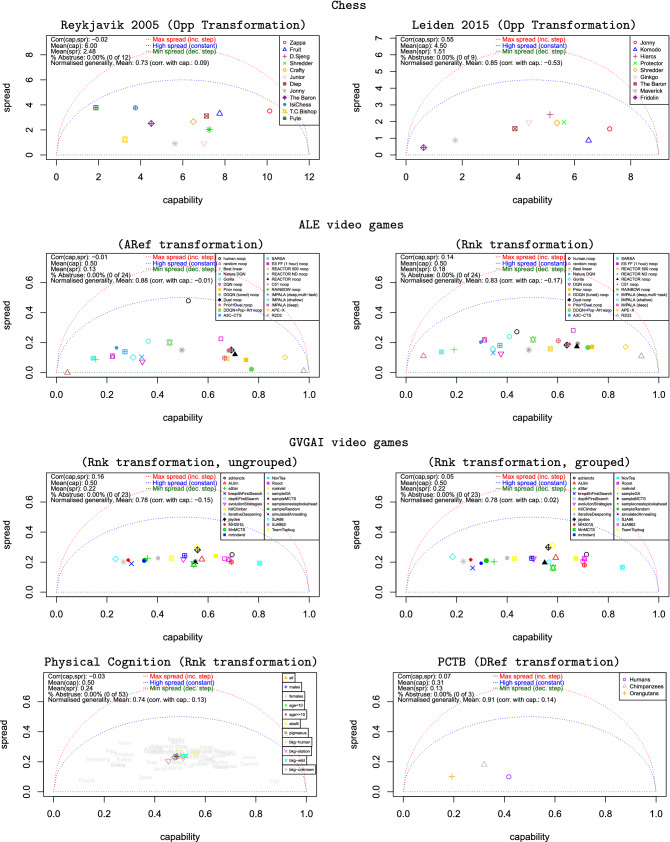
Capability and spread for several scenarios using the extrinsic difficulty transformations as per Table 1. First row: participants in the World Computer Chess Championship using the final score of the opponent as difficulty. Details in §A.6. Left: Reykjavik 2005 with 12 participants. The winner (Zappa) and the last one (Fute) won and lost all matches respectively except the one between them, which was surprisingly a draw. Right: Leiden 2015 with 9 participants. Here, no low-rank participant beat any high-rank participant, and draws were usually between participants with close scores. Accordingly, the average generality is higher in this case. Second row: ALE video games with 23 AI systems and the human reference on 45 games. Details in §A.7. Left: using the ARef transformation over a human reference. Right: using the Rnk transformation. Third row: 23 AI systems on 49 games. Details in §A.8. Left: each game has 5 variants and they are treated separately, as 245 instances. Right: all variants are aggregated together for each game (23 items). In both cases, we use the Rnk transformation. Bottom row left: physical cognition of 53 orangutans over five physical cognition tasks. Details in §A.9. Individuals represented by the names of the orangutans, with different group aggregates shown in colours. We use the Rnk transformation. Bottom row right: three different primate spaces for the Primate Cognition Test Battery^44^. Details in §A.10. We use the DRef transformation.

Figure 5 summarises the results of the cases Opp, ARef, Rnk and DRef with some experimental scenarios, including chess competitions, different video game collections, and physical and social cognition, with humans, other primates and AI systems. A detailed description of each particular scenario is found in §A, and an analysis of the transformations is covered in §F.4.

### Summary of results

Table 2 summarises some key indicators for all the scenarios seen in the paper. The values of mean normalised generality are an indication of how much of the population behaviour is ‘explained’ by the difficulty metric. This is relatively high in all cases, except for Elithorn’s mazes and especially the Odour Span Task, as we already discussed. The correlation between generality and capability shown in the last column of the table indicates whether SLODR holds for these scenarios. Except for iris using KDM difficulty, there is no relevant diminishing return of generality for increasing capability. Thus, the hypothesis, expressed with the new capability and generality metrics, is not supported by the experimental results.

**Table 2:** Summary of the scenarios and subjects seen in the paper. Difficulty modalities as per Table 1. The last two columns show the mean normalised generality and its correlation with capability.

Scenario	Subjects	Difficulty	Mean ($\gamma$)	Corr ($\gamma$,${\Psi }$)
Elithorn’s mazes	Humans	Intrinsic	0.31	0.03
Letter series	Humans and machines	Intrinsic	0.41	0.32
Object recognition	Humans, macaques and machines	Intrinsic	0.54	0.92
Odour span task	Rats	Intrinsic	0.28	0.16
Iris (KDM)	Machines	Intrinsic	0.75	− 0.32
Iris ($TD_U$)	Machines	Intrinsic	0.84	0.11
Chess (Reykjavik)	Machines	Opp	0.73	0.09
Chess (Leiden)	Machines	Opp	0.41	0.32
ALE video games	Machines (+ human reference)	ARef	0.88	− 0.01
ALE video games	Machines and human	Rnk	0.83	− 0.17
GVGAI games (agg.)	Machines	Rnk	0.78	0.02
Physical cognition	Orangutans	Rnk	0.74	0.13
PCTB	Humans, chimpanzees and orangutans	DRef	0.91	0.14

After this summary, we now cover how the newly introduced notion of generality is related to and can be interpreted within the fields of human intelligence (the psychometric perspective), animal cognition (evolutionary perspective) and artificial intelligence (computational perspective). Each subsection below is respectively supported by §B, §C and §D, where related work is covered more thoroughly.

### Psychometric interpretation

IRT models usually identify one to three parameters on the item side, such as difficulty, discrimination and guess, but only one, ability, on the agent side. Difficulties depend on the estimated abilities, and vice versa, so there is a chicken-egg problem that the algorithms break with some assumptions about the distribution of these parameters and the shape of the agent’s curve. In our case, we do not estimate any model. We avoid the chicken-egg problem by starting with a metric of difficulty in the first place. Then the procedure is simpler: we just calculate capability as an aggregate metric (the area) whereas generality depends on the relative position of these points in the $x$-axis. While the approaches are very different, §B.1 gives deeper details about the connection between generality analysis and IRT models, person-fit metrics, Guttman scale and reliability measures.

The clearest and most insightful connection happens when we consider the idealistic case that the probability of a correct response depends on the (complementary) CDF of a normal distribution using the difference between the ability and the instance difficulty. In this case, we prove (see §F.2) that spread is equal to the standard deviation of this normal distribution.

In the analysis of human (and animal) intelligence, the notion of generality is commonly associated with the ‘positive manifold’^6^, where subjects that are good for some tests are expected to be good for the rest. When factor analysis discovers that there is one single factor behind this, it is usually called *g*, for general factor. However, the value of *g* just means how strong this factor is in the population. There is no way of deriving an individual generality from *g*, and for a population with a strong manifold and a high *g*, we could still have a few very specialised agents. Again, factor analysis and our generality analysis are very different, but we can relate them by aggregation. On the one hand, if all the individuals in a population have perfect generality this implies a diagonal response matrix (if items are ordered by difficulty) and the mean correlations will be high, but not perfect. On the other hand, if we had a perfect manifold, this would necessarily mean that all agents have perfect generality. Also, the transformations that are shown in Table 1 can lead to much closer connections between the manifold and generality (this is further illustrated in §B.2). In addition, a full discussion about the SLODR, the related controversy and how this law maps to generality can be found in §B.3. Finally, the connection of generality, collective intelligence and the *c* factor is explored in §B.4.

### Evolutionary interpretation

The focus on comprehensive accounts of general intelligence in animals is usually put on whether the species has a strong *g* (intra-species) and how its results compare with other species through *G* (inter-species). This has led to endless discussions (as covered by Burkart et al.^8^) on whether the correlations might be a produce of something that is not general intelligence, possibly due to dimensionality reduction issues^9^. The confusion has its roots in a lack of clarity in the definitions of general intelligence, which in some cases are linked to results on tasks, while in other cases are linked to processes.

But generality in animal cognition and evolutionary theory also deals with notions of modularity and plasticity. In the literature we find attempts to measure intelligence for various biological systems^45^, quantifying their emergence, self-organisation^46^ and further aspects of collective dynamics^47^ showing this plasticity. In simple organisms this is usually achieved collectively, while in other species this can even be achieved individually. In any case, the key question is whether the group or the animal has a small repertoire of domain-specific functionalities or can cope with a wider range of cognitive tasks^48^. But, as in the human case, the notion of difficulty is not related to this diversity. Again, tests for animals are designed with a narrow difficulty range so there is variance in the results (§C.1 covers this lack of progressive difficulties in comparative cognition).

The connection with difficulty does finally appear when resources are mentioned in terms of cognitive demands, neural tissue, brain size, etc. The more uncertain the distribution of tasks is, the more it pays off to put the resources on the easy tasks. Through generality analysis we can now understand many trade-offs between performance, efficiency and flexibility^27^ as a result of the compactness property (#2). §C.2 and §C.3 give a more detailed analysis of these issues, including simulations with various degrees of pressure for performance and resources.

With the metrics of generality and capability, we can move beyond the crisp divide between domain-specific and domain-general performance^49^ and frame the question of general intelligence in terms of how general a species is according to the range of tasks it solves and their difficulty. ACCs can help analyse this visually whereas the metrics can help analyse this numerically.

### Computational interpretation

Generality has been a controversial topic in artificial intelligence, often blamed as the reason why AI did not meet expectations^11^, and still limiting what it can do^50^. The term artificial general intelligence (AGI) was introduced to emphasise that current AI is very narrow; specialised algorithms perform well for certain pockets of problems, even with superhuman^51^ performance, but cannot generalise to other domains. Instead of a qualitative notion of AGI, we have proposed to understand generality as a gradual feature, which *individuals* could possess in higher or lower degrees. A metric of generality should be one key indicator for understanding machine behaviour in a range of situations^52^, and contribute to the challenge of comparing human, animal and machine cognition, from basic perception^38,53–55^ to cognitive skills^56,57^.

Another part of the controversy originates from the use of two related concepts: generality and generalisation. In machine learning, generalisation^58,59^ is related to bias and distributional shift, but can ignore generality already existing in the train distribution^60^. Under the interpretation of generality we have introduced in this paper, *a general system would simply have a strong bias in favour of easy problems*. While some AI technologies have progressed for both capability and generality (the AlphaGo to MuZero series is a good example^61^), other approaches, such as large language models, are reasonably good at a wide range of very simple tasks^62^.

At a more theoretical level, a notion of intelligence as performance for all possible tasks has become popular, using a distribution containing all computable tasks^3^. But how do we assign probabilities to all possible tasks so that we can calculate expected performance over all of them? Again, the way out is to consider difficulty in the first place, and to give higher probability to those problems that require fewer resources to be solved^14^. This view is completely consistent with the measure of capability and generality introduced here, as we elaborate in §D.1 and §D.2, using theoretical distributions of tasks and algorithmic metrics of difficulty.

## Discussion

Given a battery of tasks, we may ask how many tasks an agent can solve, its overall performance. However, if we want to know how far, on average, an agent can reach in terms of task difficulty, we have to calculate its capability, as defined in this paper. Generality, on the other hand, deals with the *distribution* of the tasks the agent can solve, also in terms of difficulty. Namely, is the agent able to solve a wide variety of tasks up to some level of difficulty? If so, then the agent has high generality. Is the agent able to solve some very difficult tasks but fails catastrophically on trivial tasks? If so, the agent has low generality. These questions are previous to identifying the different domains where specialisation may occur.

When we look at an agent characteristic curve, the notions of capability and generality appear as the two most descriptive indicators to summarise the curve. However, most approaches in the analysis of human and animal intelligence somewhat assume a (decreasing) monotonicity between response and difficulty, and the whole analysis ends up mixing capability and generality. In the end, there is some circularity if we try to analyse general intelligence (and derive the *g* factor) and at the same time we assume that agents are going to show a (decreasing) relation between response and difficulty. This problem is observed, for instance, in the odour span task (OST). It was assumed that a rat would be able to perform perfectly until a certain the number of stimuli, falling sharply to zero afterwards. However, we observe that the individual generalities are low, meaning that a (slight) decline in performance happens well before and continues well after any capability point. This puts into question the assumption of a ‘working memory capacity’ beyond which the curves should sharply decline.

By decoupling generality from capability, there is now *variability in the generality* that has to be explained as well, and can be done at the individual level. We can also analyse where the generality and capability values locate for several individuals. Overall, generality can increase or decrease individually or collectively, through evolution, development or design (as we have shown for the video game benchmarks). If resources are a (selective or designing) pressure, then the compactness property (#2, with equal capability, any redistribution of success to easier, less expensive tasks increases generality) indicates that generality becomes beneficial.

In the experimental scenarios, we have observed how capability and generality relate, with no other assumptions being made except for the choice of a difficulty metric and its range. The scenarios are different as well, but the results suggest that humans tend to be more general than other groups (other animals and present-day AI systems). In complex tasks such as object recognition, the resource pressure usually creates a strong correlation between capability and generality, which is observed for humans, macaque monkeys and deep learning architectures. Consequently, we do not see support for SLODR—rather the opposite—at least when Spearman’s law is re-interpreted with our measures of generality and capability.

Generality analysis differs from other kinds of analysis. The main difference is that the results from factor analysis, PCA or IRT are populational. A very abstruse agent that is only good for a pocket of (hard) problems, if added several times to the population—and this can be done with AI systems today very easily—can change the population so dramatically that the other agents become the special ones. This does not happen for the measures of capability and generality if we use an intrinsic difficulty metric.

When using intrinsic difficulties, there are some methodological issues to consider and recommendations to make. We need to derive difficulty from enumerative computer models, psychophysical distortions, number of elements, reference agents, measures of resources, and other approaches. It is also important that we find a wide “variability in task difficulty”^25^ so that all agents can reach maximum performance for very easy (trivial) problems and drop to minimum performance for very difficult (really hard) problems. By doing so we can ensure no ceiling effects happen, to really have translation invariance.

We have illustrated how this new generality analysis works for a wide range of tasks and subjects, but there are so many other scenarios to explore. For instance, our experiments have illustrated the metrics with consolidated tasks, where actual performance is evaluated, but they could be applied to potential or developmental settings. In order to determine whether a system is able to *acquire* new skills, the tasks should be designed to represent the ability to learn new skills and knowledge, rather than the actual performance once the task has been acquired. Instances of these tasks can be created, and their difficulty estimated, depending on how much exploration and training information is given, and conditioned to other previous skills that are necessary for acquiring the new task. In this context, the difficulty function would turn into a more complex *graph* of dependencies. For instance, succeeding in finding food in an environment depends on recognising food in the first place. This means that a system that is able to do the former but not the latter could not be called a generalist, at least in a developmental sense. A proper extension of this schema for learning tasks (where difficulty is also linked to levels of assistance, such as demonstrations and teaching) or developmental situations (where difficulty depends on what is assumed about some other skills or the relation between the skills that compose several tasks) is a full research programme for the years to come.

There are many other future research opportunities. First, there is much to do to further clarify existing and newly-introduced metrics of difficulty for different kinds of tasks, and to use them in the analysis of results. It may even be possible to develop a universal measure of difficulty for all tasks^20^. Second, by using ad hoc measures of difficulty (even if they are imperfect), we can revisit a myriad of results already collected for humans, non-human animals and AI systems, and see them through the prism of a generality analysis. Third, generality can help rebuild the narratives and conceptions of intelligence beyond a monolithic score—or a multiplicity of scores—that individuals would have in higher or lower degrees. Instead, each individual would be distinguished by characteristic curves, with capability and generality just being its key indicators.

Overall, the resolution of the conflation between generality and capability reframes the question of what “General Intelligence” is and how it can be measured. For the evaluation of humans and other animals, it can become an alternative or complement to populational techniques. In machines, generality can help recover the meaning that the G in “Artificial General Intelligence” was originally meant to have.

## Supplementary Information

Supplementary Information.
