# Supplementary material for: General intelligence disentangled via a generality metric for natural and artificial intelligence
Source: Sci Rep. 2021 Nov 24;11:22822. doi: 10.1038/s41598-021-01997-7 (PMC8613222; doi:10.1038/s41598-021-01997-7)
Supplement: Supplementary file 1 — Supplementary Information. [file 41598_2021_1997_MOESM1_ESM.pdf]

Supplementary Material

Supplementary Material

"General Intelligence Disentangled via
a Generality Metric for Natural and Artificial Intelligence"

José Hernández-Orallo[1,2], Bao Sheng Loe[3], Lucy Cheke[2,4],
Fernando Martínez-Plumed[5,1], Seán Ó hÉigeartaigh[2,6]

[1] VRAIN, Universitat Politècnica de València
[2] Leverhulme Centre for the Future of Intelligence, University of Cambridge
[3] Psychometrics Centre, University of Cambridge
[4] Department of Psychology, University of Cambridge
[5] Joint Research Centre, European Commission
[6] Centre for the Study of Existential Risk, University of Cambridge

In this supplementary material, we first include more details about the experimental results and the case studies. Then we perform a more thorough interpretation of the newly introduced notion of generality from the psychometric perspective (person-fit, IRT, SLODR and the c factor), an evolutionary perspective (g and G factor in animals, cognitive resources, selective pressure) and a computational interpretation (AGI, competitions and benchmarks in AI), properly covering the related work in these areas. Finally, we give further insights into the formal definitions, the properties of generality and the proof of the theoretical results.

A Case studies: details and extended experimental results

The following subsections extend the details about the experimental results shown in the paper.

A.1 Elithorn's mazes

Elithorn's Perceptual Mazes [1] take the form of a V-shape triangle, as shown in Fig. S1, where the coloured dots are superimposed at the intersection of the pathways. The imposed structure of the V-shape maze reduces the number of uncontrollable variables and holds the shape of the task constant. The goal is to collect as many yellow dots using a pathway up to the top. Three conditions must be fulfilled in order to successfully complete the task: (1) the pathway must lie along the lattice line; (2) the pathway can only move in an upward orientation and stay connected at all times; (3) the pathway must pass through the route with the maximum number of yellow dots. Therefore, it is critical that the subjects strategise before they begin working on the task.

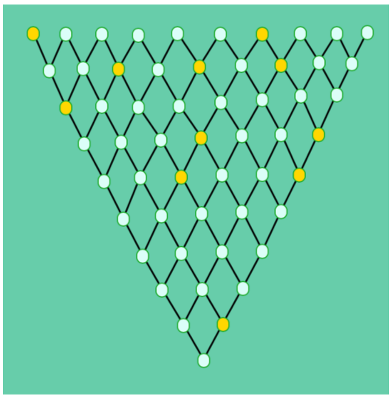

Figure S1: Maze of rank 10 with 30% saturation.

Given the structural nature of the task, several parameters of the maze have been considered to influence task difficulty. Previous research found that the two main physical properties that were most pronounced to influencing the difficulty of the maze were the size of the maze (rank) and the density of the pattern (saturation level) [2, 3]. The assumption is that the maze difficulty typically increases with size, and monotonically with saturation up to about 50 percent, where it begins to decrease monotonically thereafter [3]. In a separate research, Davies & Davies (1965) proposed that a maze would be found more difficult if the number of steps required to pass through the optimal route was greater. Thus, this was considered as a third variable which was used to calculate maze difficulty.

The physical parameters used to calculate maze difficulty led to several authors proposing different mathematical models of difficulty [4, 2], which are then *intrinsic* difficulty metrics, in contrast to extrinsic (i.e.,

Table S1: Intrinsic difficulties for the 23 items.

Item	Difficulty	Item	Difficulty
X247	9.53	X259	16.32
X248	8.88	X260	16.81
X249	11.01	X261	18.05
X250	10.21	X262	19.12
X251	12.16	X263	19.41
X252	11.67	X264	18.26
X253	13.26	X216	11.41
X254	13.54	X217	11.73
X255	14.91	X225	14.10
X256	14.91	X227	14.93
X257	15.77	X233	10.11
X258	15.77		

Table S2: Demographics Information.

	n	%
Gender		
- Male	301	61
- Female	195	39
Education		
- PhD	16	3.2
- Masters	90	18.1
- Undergraduate degree	204	41.1
- Vocational	28	5.6
- High School	137	27.6
- Secondary school	16	3.2
- Primary school	5	1
Ethnicity		
- White	288	58.1
- Asian	145	29.2
- Black	2	0.4
- Mixed ethnic groups	20	4
- Other	41	8.3
Nationality		
- United States	87	17.5
- United Kingdom	32	6.5
- India	107	21.6
- Germany	21	4.2
- Poland	20	4
- Others	229	46.2

psychometric or populational) difficulty. In particular, we will use the method proposed by [2]. Following their notation, 2^R is the total number of possible paths to pass in the maze and $U_{\hat{m}}$ is the number of distinct paths that allow one to achieve the maximum number of dots for a given maze. For example, if the maximum number of dots that is permissible to pass is 3, then $U_{\hat{m}}$ is the number of unique routes through the 3 dots. The saturation level (S) is an expression for maze density. Finally, the number of steps l required to achieve the greatest score for any given maze was included as well. The final version of the difficulty function is:

$$\hbar = \log_{10}\left(2^R \times \frac{S^a \times l^b}{U_{\hat{m}}}\right)$$

We used the recommended parameters $a = 4$ and $b = 4$ in the `mazeGen` R package [5], following [2]. Using this package, we generated 23 items with a range of difficulties. The difficulties are shown in Table S1. We administered them to 530 participants via the Cambridge Psychometrics Centre testing website[1]. The testing website has over 20 different psychological tests that are made publicly available for people who have access to the Internet to complete. They have the option to stop completing the test at anytime. Participants do not receive any form of financial incentives, but they are encouraged to complete by providing immediate feedback after the end of the test. Responses are binary. The respondent either found an optimal path or not. Of the 530 participants who completed the test, only 496 provided their demographics information Table S2, to which we restricted our analysis.

Fig. S2 shows the means of the 496 responses for each item vs difficulty. The Pearson correlation is -0.49, which is reasonable (more difficult items get worse responses). This is sufficiently high to consider the difficulty metric to be fit for our purposes.

Fig. S3 perfoms the generality analysis per subpopulations, corresponding to Fig. 4 (top left) in the main paper.

There are several ways in which we can analyse whether the respondents have a general behaviour. One simple option seems to calculate the mean result per respondent and their variance. Fig. S4 shows that this is completely uninformative in this case, as the variance is determined by the mean for binary outcomes, and the variance of a Bernoulli distribution is $p(1-p)$, being a function of the mean response p. In any case, even if the responses were quantitative, one would not expect respondents to have low variance if a wide range of difficulties is considered, because most would fail at the difficult ones and would succeed at the easy ones. Actually, as it is expected that respondents fail on the difficult items, if there are some of these, very high generality —understood as reciprocal of variance— would be impossible.

A common way of analysing "generality" of a population is through factor analysis, trying to identify a dominant factor that would explain why a result for one item usually entails a similar result for other items.

[1] www.discovermyprofile.com

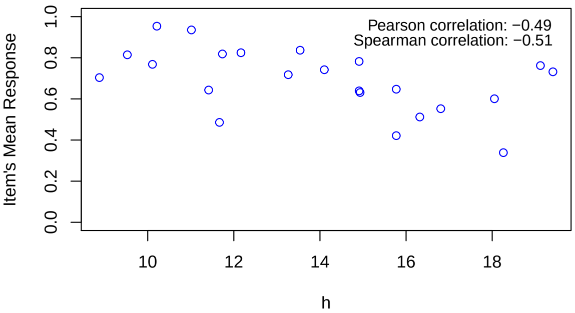

Figure S2: Relation between intrinsic difficulties and the mean responses for the 23 items of the mazes problem.

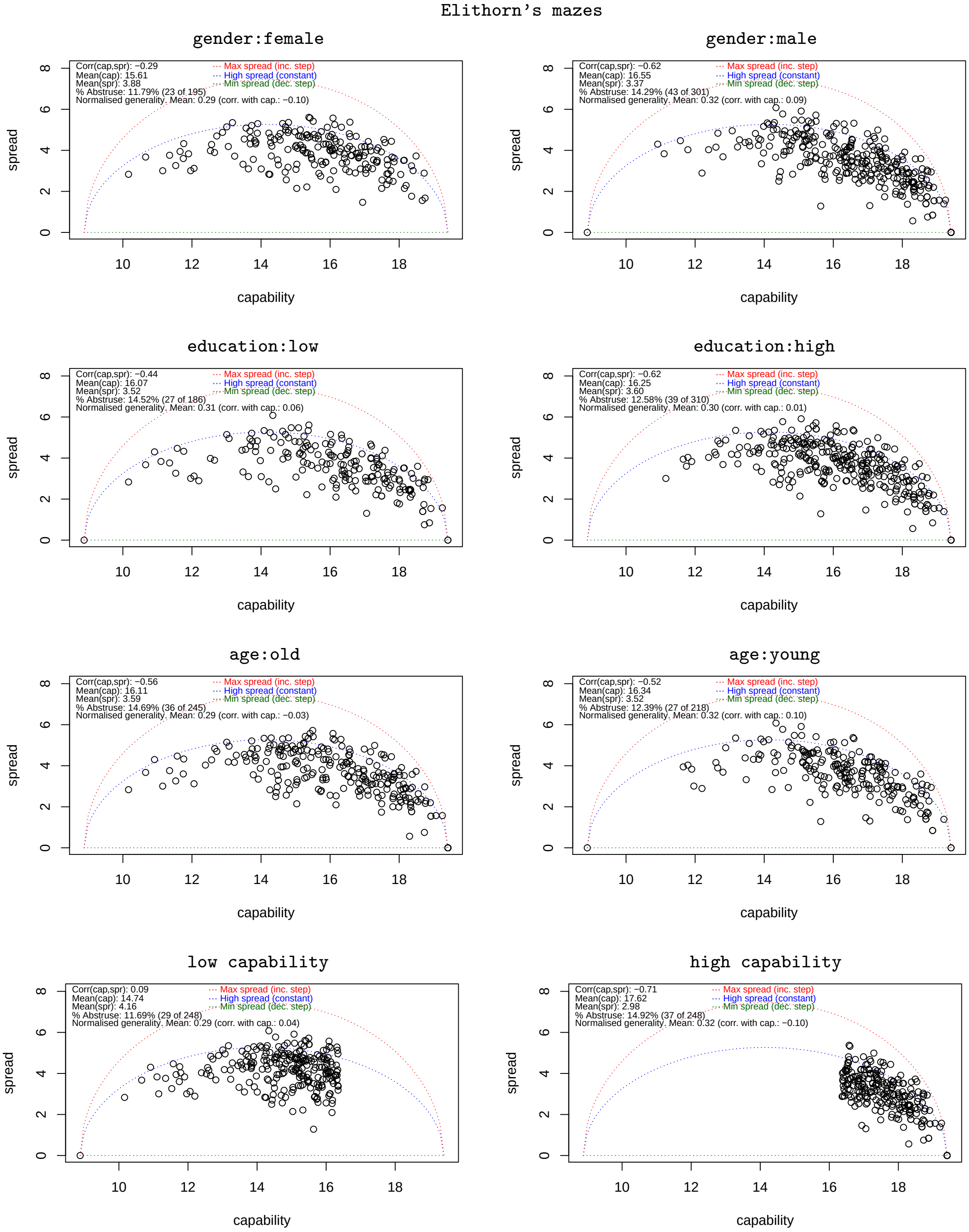

Figure S3: Detailed results for Elithorn's mazes (see Fig. 4 (top left) in the main paper). First row: split by gender. Second: split by education. Third: split by age. Fourth: split by median capability.

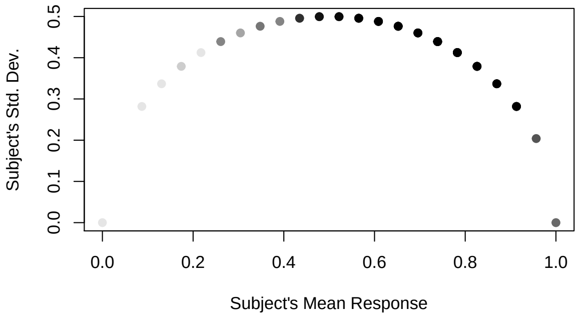

Figure S4: Subjects' means vs subjects' standard deviations of 496 respondents and 23 items each of Elithorn's Mazes. As responses are binary, the plot just shows the standard deviation of the Bernoulli distribution: $\sqrt{p(1-p)}$.

We perform factor analysis using the R package `psych` and the function `fa`. By just limiting to one factor, we get the 23 loadings for the items between 0.123 and 0.518 (average 0.342). This factor, however, only explains a proportion of 0.123 of the variance. The scree plot in Fig. S5 shows that for this population and items a single factor is not enough.

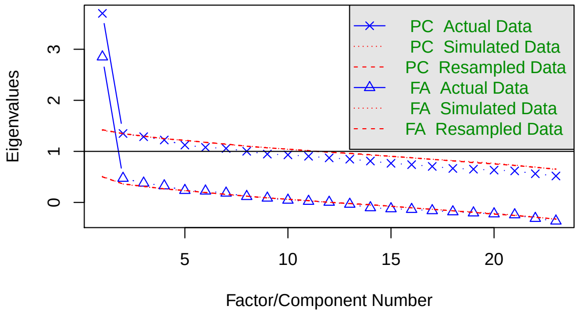

Figure S5: Parallel Analysis Scree plot for principal component analysis (PCA) and factor analysis (FA) of 496 respondents and 23 items each for Elithorn's Perceptual Mazes.

This contrasts with a reasonable degree of generality that we found using the generality analysis (see top left of Fig. 4 in the paper). Factor analysis simply ignores difficulties. Also, factor analysis just tells us whether there is a dominant factor *in the population*, but it cannot inform us on whether some particular respondents are more general than others.

Another common option to analyse this kind of results is to infer IRT models. Using the `mirt` function of the homonymous R package and a 2PL model we get the item response curves, all of them with positive discrimination parameters. The (Pearson) correlation between the estimated difficulties (the positions of the logistic models) and the intrinsic difficulties is 0.39. The (Pearson) correlation between these estimated difficulties and the response means, shown in Fig. S6, is -0.94, close to -1 as expected, which is higher than the corresponding plot with the intrinsic difficulties (Fig. S2). We now see an almost perfect monotonic relation, which basically illustrates that IRT difficulty is almost a monotonic transformation of response means, which makes it hard —if not impossible— to differentiate generality from capability.

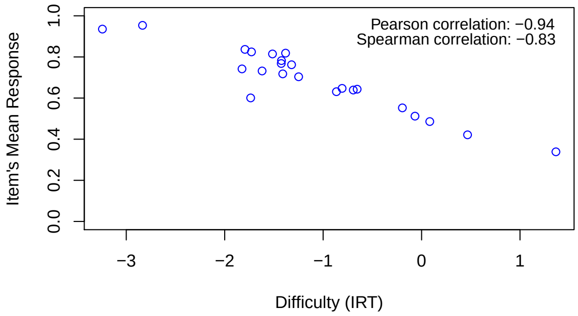

Figure S6: Relation between IRT-estimated difficulties (2PL) and the mean responses for the 23 items.

Nevertheless, we are interested in having a look at the two measures when using IRT difficulties, even if this

analysis is a bit circular (we get the difficulties from the results and then we use them to analyse the results). Fig. S7 shows a higher dispersion than for the intrinsic difficulties and, as expected, we see higher normalised generalities on average, as the 2PL models assume this for ability. Still, it may be useful to compare individual agents with this capability and generality.

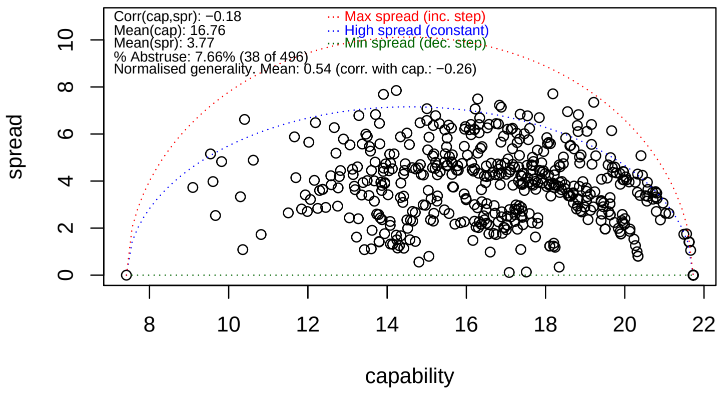

Figure S7: Capability vs spread for the Elithorn's mazes using the difficulties obtained by 2PL IRT models (scaled to the same range to have comparable units). Compare with Fig. 4 (top left).

A.2 Letter series

Thurstone letter series is a common cognitive test that was introduced for his Primary Mental Abilities theory [6, 7]. Given a sequence of letters from the Latin alphabet (where the successor of 'z' is 'a' and the predecessor of 'a' is 'z'), the goal is to predict the next letter in the series. Fig. S8 shows some examples.

$$
\begin{array}{l}
a, b, a, b, a, b, a, b, \ldots \\
a, a, a, b, b, b, c, c, c, d, d, \ldots \\
c, a, d, a, e, a, f, a, \ldots \\
w, x, a, x, y, b, y, z, c, z, a, d, a, b, \ldots \\
m, n, l, n, k, n, j, n, \ldots \\
r, s, c, d, s, t, d, e, t, u, e, f, \ldots
\end{array}
$$

Figure S8: Examples of Thurstone letter series (taken from [8]).

In [9, 10] a sequence generator based on computational principles and algorithmic information theory. The generator was built to ensure stability of the sequence (they should not have exceptions or noise) and its unquestionability (the shortest program that generates the sequence should not be rivalled by another program of similar complexity that gives a different continuation to the series). The generation also calculated difficulty for each sequence x, derived as $Kt(x)$, its Levin complexity [11, 12] over a minimal instruction set computer with 14 instructions. Fig. S9 shows a sample of sequences and their intrinsic difficulties.

$$
\begin{array}{lcll}
h = 7 & : & a, b, c, d, \ldots & Answer : e \\
h = 8 & : & a, a, a, b, b, b, c, \ldots & Answer : c \\
h = 9 & : & a, d, g, j, \ldots & Answer : m \\
h = 10 & : & a, c, b, d, c, e, \ldots & Answer : d \\
h = 11 & : & a, a, b, b, z, a, b, b, \ldots & Answer : y \\
h = 12 & : & a, a, z, c, y, e, x, \ldots & Answer : g \\
h = 13 & : & a, z, b, d, c, e, g, f, \ldots & Answer : h \\
h = 14 & : & c, a, b, d, b, c, c, e, c, d, \ldots & Answer : d
\end{array}
$$

Figure S9: Examples of some of the sequences and their intrinsic difficulties, as generated for the C-test [9, 10].

Reusing this study, we take 35 instances: 20 for prediction as shown in Fig. S9 and 15 for imputation (the gap is in the middle of the sequence) with difficulties ranging from 7 to 14. All these sequences were applied to 48 young humans with ages between 14 and 18 [9, 10].

Fig. S10 shows the means of the 48 human responses for each item vs difficulty. The correlation is −0.77, which is quite strong (more difficult items get worse responses). For our study, we also added new results from

12 artificial systems, as shown in Table S3. The generality analysis was shown in Fig. 4 (top right). We show a human-machine split in Fig. S11.

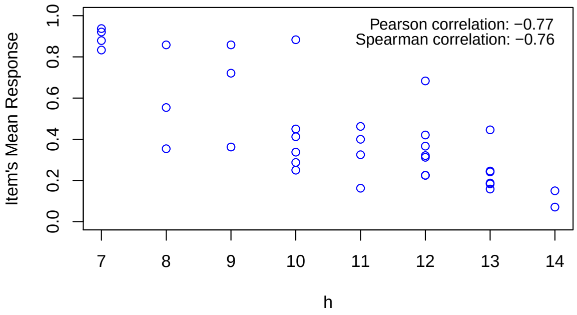

Figure S10: Relation between intrinsic difficulties and the mean responses for the 35 items of the letter series problem.

Table S3: Artificial systems used to solve the letter series.

System	Description
`freq`	Predicts or imputes the most frequent letter.
`last`	Predicts or imputes the letter just before the end or the gap.
`x`, `y`, `z`, `a`, `b`, `c`	Constant models always predicting or imputing the same letter.
`repdiff`	Predicts or imputes according to an arithmetic series.
`alternate`	Predicts or imputes using an arithmetic series using only the even or odd letters depending on whether the gap is at an even or odd location.
`halfright`	A baseline model predicting or imputing correctly on about half of the sequences (0/1 chosen randomly).
`magic`	Using the web version (`nautilus.cs.miyazaki-u.ac.jp/~skata/MagicHaskeller.html`) of MagicHaskeller, an inductive programming system [13, 14, 15].

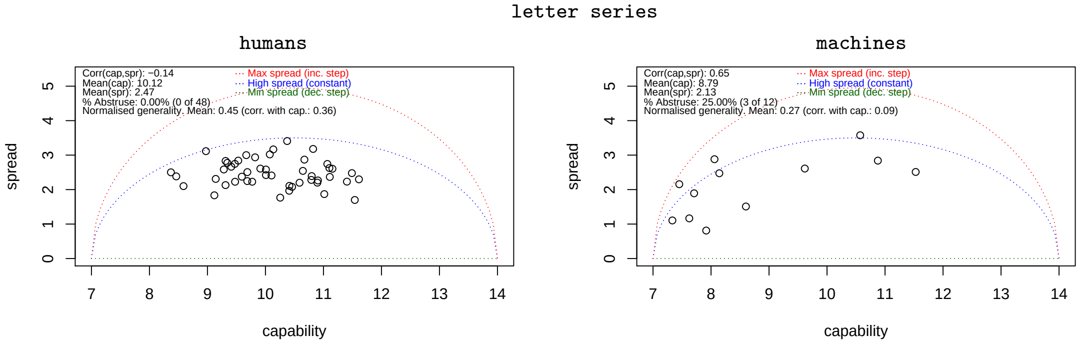

Figure S11: Capability vs spread for the letter series showing human-machine split of Fig. 4 (top right).

We also perform factor analysis on this data, using the same methodology as in the previous case, focusing on the 48 human respondents. As a result, we get the loadings for the 35 items with values between −0.212 and 0.593 (average 0.215). This is very poor, and the first factor only explains a proportion of 0.093 of the variance. The scree plot in Fig. S12 shows that for this population and items a single factor is not enough. Again, this contrasts with a reasonable degree of generality that we found using the generality analysis (Fig. S11, left).

Finally, we also infer IRT models for this case. The Pearson correlation between the estimated difficulties (the positions of the logistic models) and the intrinsic difficulties is 0.02, but the Spearman correlation is 0.50. Then, the correlation between the IRT difficulties and the column means (item's mean response, a proxy for difficulty) is 0.00 (Pearson) and −0.60 (Spearman), as we can see in Fig. S13. While difficulties should correlate negative with responses by construction in IRT, this case is explained by a difficulty value on −150, an outlier difficulty value. Compare with the same plot with intrinsic difficulties (Fig. S2). In this case, we should analyse what the scale of these difficulties mean, the exclusion of outliers and whether we can derive generality from here. We do not explore this option further for this case.

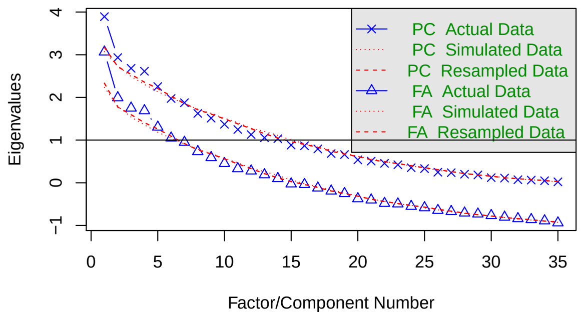

Figure S12: Parallel Analysis Scree plot for principal component analysis (PCA) and factor analysis (FA) of 48 human respondents and 35 letter series.

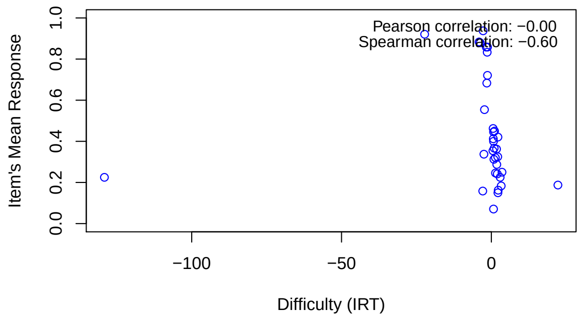

Figure S13: Relation between IRT difficulties (2PL, estimated from the human population) and the mean responses for the 35 letter series.

A.3 Object recognition

For the object recognition scenario we used a sample of the results involving 24 objects with 10 variations each, totalling 240 cases, referred to as "primary test images" and shown in [16, Fig.1A]. Each result in our dataset is the aggregation of each object (e.g., a zebra with a given distortion) being confronted against the other objects (e.g., the non-distorted zebra image vs a non-distorted dromedary, a non-distorted zebra vs a non-distorted guitar, etc.). Because agents have to choose between two images, expected accuracy by chance is 0.5, so we normalised accuracy as $2x - 1$). The agents are six deep convolutional artificial neural networks: ALEXNET, ZEILER, VGG, GOOGLENET, RESNET and GOOGLENETv3 (inception), the monkeys (macaques) and the humans. In total we have a non-binary response matrix of 8 rows and 240 columns.

Fig. S14 shows the (unnormalised) average accuracy of the eight agent groups for the 24 objects in this study. We see that some objects are harder than others, but we will not use this information initially (just the psychophysical attributes).

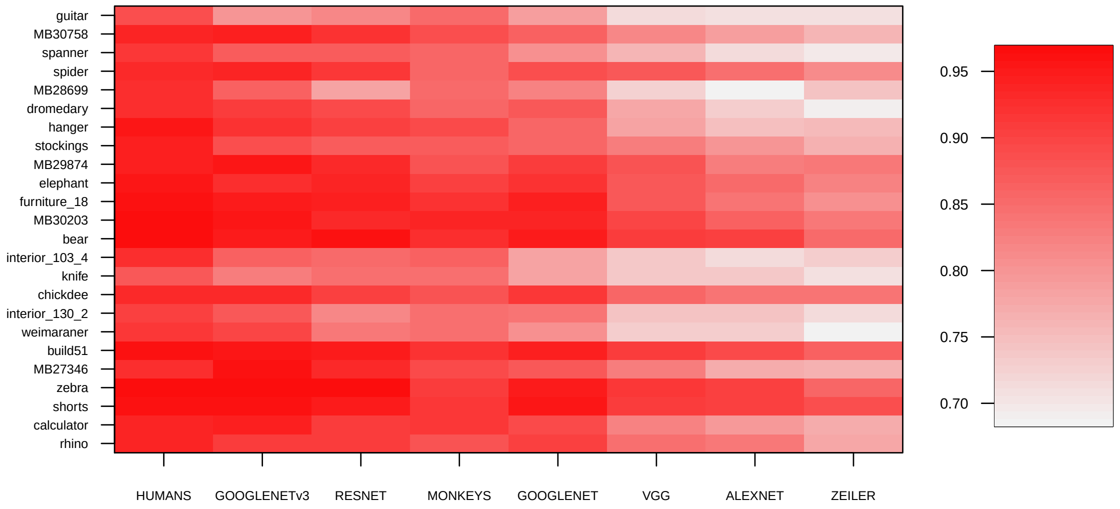

Figure S14: (Unnormalised) accuracies for each the 8 agent groups (2 primate species and 6 ANN architectures) against the 24 objects we use in our study.

The psychophysical distortions are created by modifying four image attributes: *eccentricity*, *size*, *pose* and *contrast*. Not all of them affect the problem monotonically. For instance, *pose* has a minimum of performance at intermediate values, as was shown in [16, Fig.7C]. We derived a formula of difficulty that takes this into account:

$$\hbar \stackrel{\text{def}}{=} s(eccentricity) + s(-size) + s(pose') + s(-contrast)$$

where $s(\cdot)$ performs a min-max scaling and $pose' = -|1 - pose|$, as the minimum distortion is around $pose = 1$. We binned difficulties into 15 equal-width binning, and we removed those bins with less than 4 difficulties, retaining 233 results from the original 240 cases, filtering the response matrix accordingly.

The ACCs for the eight agent groups can be seen in Fig. S15. Humans and monkeys have smoother curves. As the population size is high for humans but small for macaques, this is not clearly explained by sample size, and may be indicative that the effect of the psychophysical distortions is more gradual for primates than ANNs.

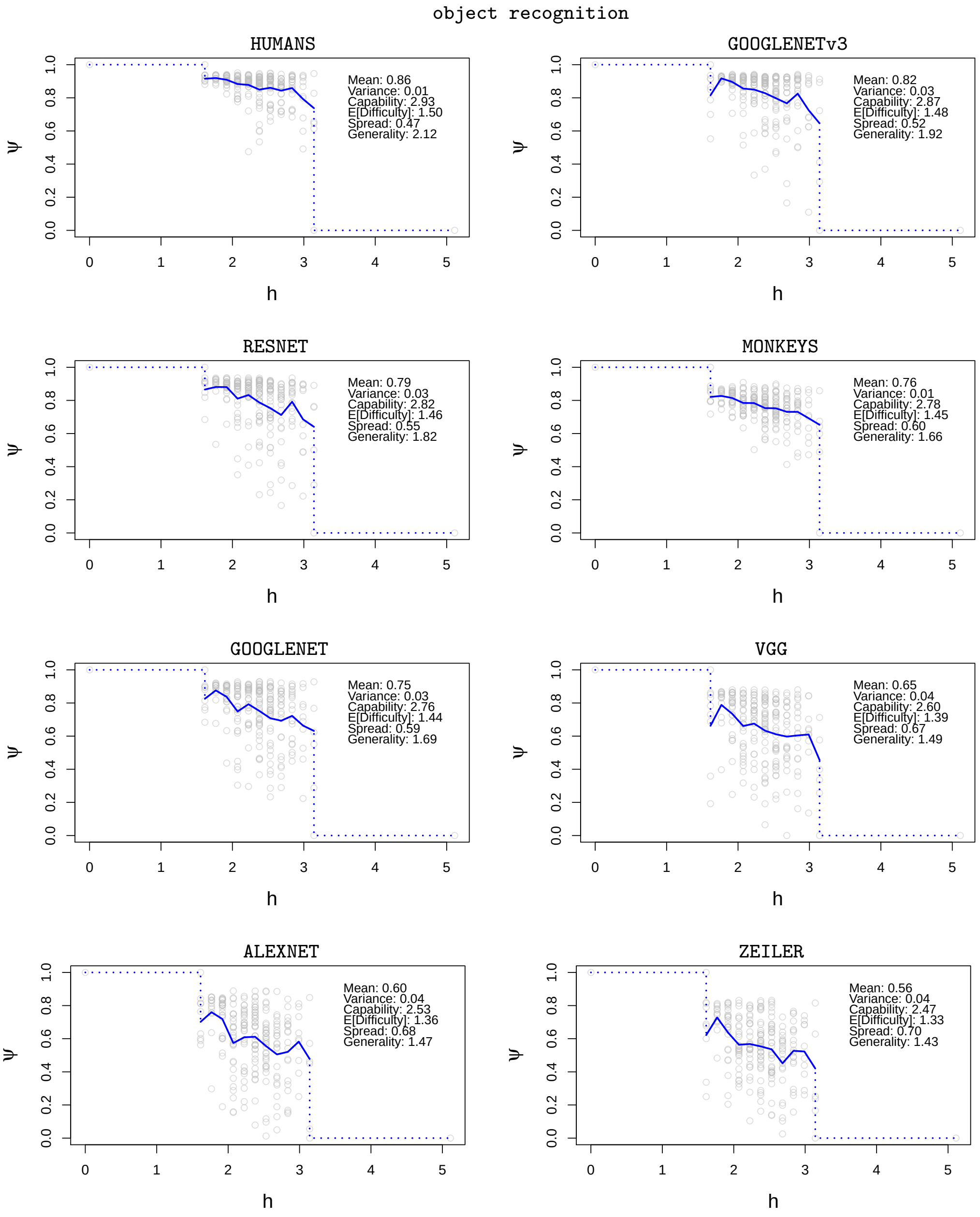

Figure S15: ACCs for the eight agent groups in the object recognition problem. Intrinsic difficulty derived from the psychophysical parameters.

The results of the generality analysis are shown in the main part of the article. In this case, as we only have 8 agent groups, we cannot do factor analysis or IRT. A populational estimation of difficulty is outlined in [16, Fig.2] by analysing the "behavioural signatures" (very much like what we show in Fig. S14) or approximated by the "ones with performance below the 25th percentile". Note that these difficulties are per object, where they

determine that recognising a camel is more difficult than recognising a watch. With the difficulties derived from the psychophysical parameters, we are not using the information about the object, just the level of distortion, regardless of what object has to be classified. With this, we leave a great deal of the difficulty not been included in the metric we are using. This is consistent with the fact that the proportion of variance that is explained by the psychophysical parameters is very small (< 0.1) [16, Fig.7D].

A.4 Odour span task

There were two main reasons for choosing the Odour (or Olfactory) Span Task (OST), and experiment 2 in [17] in particular. The first one is that this task has items with an associated difficulty that is very natural (number of scents to remember). Since capability and spread are in the same scale as the difficulty metric, the interpretation is very intuitive and it even has units: number of scents. Note that many other metrics of performance are *unitless*, such as accuracy. The second reason for choosing this task is that span length is a very interesting metric, and does have units (also number of scents). Actually, if the generality of an agent were maximum, span length would be exactly equal to capability (and exactly equal to longest run), as the agent would be able to perform perfectly until the number of stimuli reaches its capability, falling sharply to zero afterwards.

However, this extreme does not seem to happen for this experiment. Actually, longest run is usually larger than span length, which means that some good streaks are obtained when the rat has already seen many stimuli. Looking back to Fig. 4 (middle right), we can notice that rats have very high capabilities (the average is 50.04 scents), when we think that this really means the number of scents they are able to remember. In particular, five of the ten rats (T12, T13, S17, S1, V20) had no previous exposure to the stimuli before the experiment, so whenever they had to choose between the new scent and the old scent in the experiment, the new scent was totally new. This means that these rats did not have to distinguish between the old scents, just being able to tell if the scent was new, which could be linked to some basic olfactory physiology rather than memory. These rats perform better than the other five. However, the other five rats, which had been exposed to all scents before the task, while worse, still have a capability above 45 scents on average. So this 'novelty' interpretation of the task can only explain part of this exceptional performance. Another partial explanation is combinatorial. For a very large number of stimuli, the probability that an old scent reappears is high, as it has been in the pool for a long time. On average, the probability that any scent that appeared before as new is picked again as old is close to one on expectation (it is a series adding $1/n$ each time to the probability, with n being the number of stimuli seen so far). In particular, scent 1 appears 4.85 times in expectation in an experiment with 72 odours ($\sum_{i=1}^{71} 1/i$). This basically serves as reminders that may be helpful to remember the old scents.

Taking all this into consideration, we can look at some particular examples in Fig. S16. Rat D2 has an almost monotonically decreasing curve, going down slowly from 0 to 65, where the data is discontinued. On the contrary, rat S17 is much flatter, but as it is almost perfect up to difficulty 65 (and the data is continued assuming the best possible case), we get a lower spread and higher generality. Rat F16 has an abstruse behaviour and seems to increase for higher number of stimuli. Finally, rat S1 has a very sharp decrease in performance around difficulty 50. There seems to have reached a saturation point, which would have given this rat a high generality, but the curve also sharply recovers between 55 and 65. Notwithstanding, spread is still the smallest.

The key element to understand this experiment is that we have ceiling issues. We do not really reach a number of stimuli where accuracy really falls to zero. This may be the case for larger numbers of stimuli, but as [17] recognise, that would make the experiment very hard to conduct, as 72 is already a very high number of trials. But it may well that "rats can recognize the relative familiarity of odors in some fashion that is largely independent of the number of stimuli to remember. In other words, within a given session, choices may involve an assessment of the 'newness' or 'oldness' of a stimulus in relation to the other stimuli present on a given trial, with the ultimate choice being made towards the least familiar option". Overall, independently of the ultimate cause, we have shown that generality analysis is able to detect when generality takes place, and those rats that are more or less general.

A.5 Feature-based classification

We started with 473 different machine learning classifiers from study number 7306 from OpenML [18]. We removed all classifiers with accuracy below 0.35, which is roughly what a random classifier would achieve for this problem, since there are three balanced classes. After this filter, we end up with 419 classifiers. Again, as in the previous cases, we do not consider any predefined partition of the instances in subdomains (apart from the three classes). Each machine learning technique can be better or worse for some subgroups (better in some regions, in dense areas, for some classes over others, etc.). The interesting point of our analysis is precisely this; we can derive generality without considering any a priori separation of the examples into subdomains.

Fig. S17 shows the points of the aggregated ACC (without really connecting the points of the 419 classifiers for clarity). The left plot with KDN difficulty shows points that are largely arranged in a steplike manner,

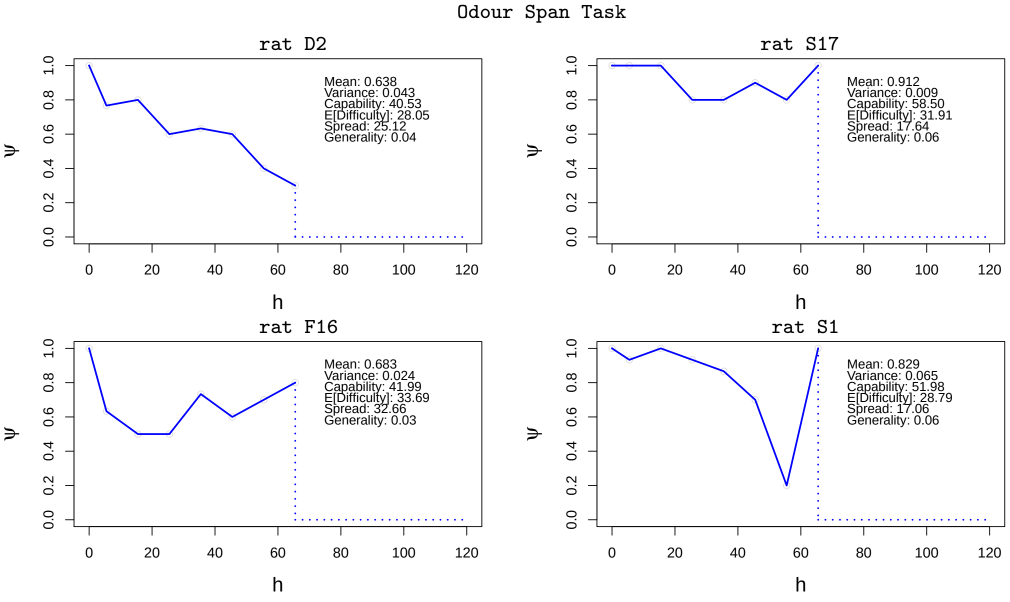

Figure S16: ACCs for four selected rats in the Odour Span Task. As there are only two choices, the accuracy of a random guess should be 0.5, so the values in the y-axis are scaled to $2(r_{j,i} - 0.5)$.

with difficulty 0.4 being the tipping point. The aggregated ACC on the right corresponds to TD_U difficulty. In this case, it is also very steplike, but the inflection point takes place at difficulty 4, with no much happening in difficulties 1 to 3.

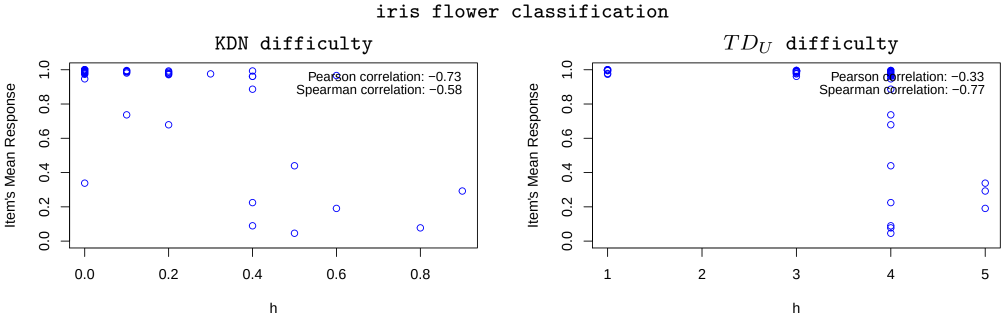

Figure S17: ACCs corresponding to the cases in Fig. 4 (bottom). Left: the intrinsic difficulty metric (KDN in this case) shows how results are high for low difficulties and low for high difficulties. Right: using TD_U difficulty instead.

Fig. S18 shows the classifiers with best and worst capabilities and generalities, from all the classifiers in Fig. 4 (bottom) (also using KDN on the left and TD_U on the right). While the best capabilities and generalities suggest what kind of methods can get good results for one or the other, the worst-case plots are simply anecdotal, produced by bad hyperparameters, as a kind of illustration of what wretched curves look like.

We can delve further into the classifiers shown on Fig. 4 (bottom). Let us identify one technique whose underlying mechanism is simple and well understood, such as logistic regression, a linear model based on the original attributes. Given some non-linearities in iris, this classifier does not reach exceptional values in capability (0.63) for difficulty KDN. However, it turns out to be very general (spread 0.17); as it is based on hyperplanes, it cannot really specialise too much. Similar results happen for difficulty TD_U: capability (4.78) and generality (0.42).

In order to have a more complete understanding of how several techniques behave under different difficulty metrics, we can look at the three best classifiers in terms of capability or generality. For KDN, the best three classifiers in Table S4 are algebraic (linear or non-linear), while the best three in generality in Table S5 are based on decision rules or decision stumps in ensembles. For a difficulty metric based on multivariate distances, high generality is given for those methods based on univariate partitions. The distinction gets more blurred in tables S6 and S7 for difficulty TD_U, but models with high generality are now in this case based on a metric space (except the decision tree ensemble, which samples on the features), while the metric is based on short descriptions. Further analysis should be needed to find consistent patterns between the type of difficulty and the generality observed for related and unrelated techniques.

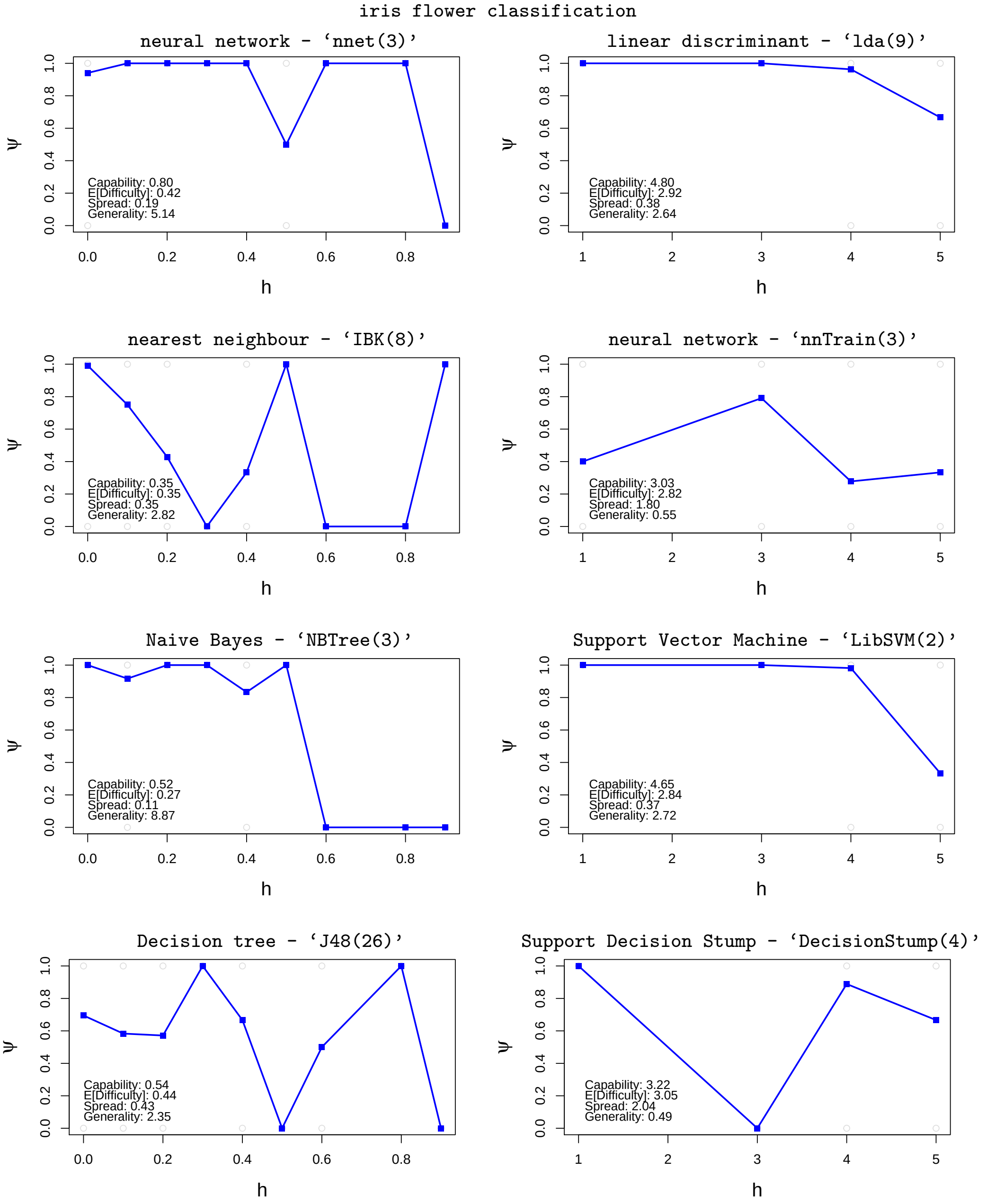

Figure S18: Some ACCs for the iris dataset, showing the most capable, least capable, most general and least general for metric KDN (left) and TD_U (right). Least capable and least general classifiers are usually obtained when methods are run with inappropriate hyperparameters.

Table S4: Capability and spread of the top three classifiers with highest capability for KDN difficulty. Data and configuration as in Fig. 4 (bottom left).

	Technique - algorithm name (parameter configuration)	Capability	Spread
1	Neural Network - ‘classif.nnet(3)’	0.797	0.195
2	Linear Discriminant - ‘classif.lda(9)’	0.783	0.215
3	Quadratic Discriminant - ‘classif.qda(2)’	0.766	0.242

Table S5: Capability and spread of the top three classifiers with highest generality for KDN difficulty. Data and configuration as in Fig. 4 (bottom left).

	Technique - algorithm name (parameter configuration)	Capability	Spread
1	Naive Bayes Tree - ‘weka.NBTree(3)’	0.525	0.113
2	Stump Ensemble - ‘weka.Dagging_DecisionStump(2)’	0.458	0.124
3	Stump Ensemble - ‘weka.AdaBoostM1_DecisionStump(12)’	0.575	0.135

Table S6: Capability and spread of the top three classifiers with highest capability for TD_U difficulty. Data and configuration as in Fig. 4 (bottom right).

	Technique - algorithm name (parameter configuration)	Capability	Spread
1	Linear Discriminant - 'classif.lda(9)'	4.80	0.379
2	Logistic Regression - 'weka.Logistic(5)'	4.78	0.416
3	Voting Feature Intervals - 'weka.VFI(1)'	4.76	0.449

Table S7: Capability and spread of the top three classifiers with highest generality for TD_U difficulty. Data and configuration as in Fig. 4 (bottom right).

	Technique - algorithm name (parameter configuration)	Capability	Spread
1	Support Vector Machine - 'weka.LibSVM(2)'	4.65	0.368
2	Decision Tree Ensemble - 'weka.RandomSubSpace_REPTree(4)'	4.44	0.369
3	Linear Discriminant - 'classif.lda(9)'	4.80	0.379

Overall, we have seen that the difficulty metric has a strong effect on the capability and generality measures, and orders classifiers differently (both in terms of capability and generality), and ultimately differently too from the order given by the model accuracies.

A.6 Chess (with Opp transformation)

The Opp transformation in Table 1 takes place in situations where the difficulty of the task depends on the opponent, as happens in many one-vs-one or team-vs-team competitions. To illustrate this case, we analyse the results of the World Computer Chess Championship (WCCC), usually part of the Computer Olympiad, where several computer chess players compete against each other. The transformation takes the score of the opponent as difficulty with values being 1 (wins), 0.5 (draws) and 0 (losses).

Fig. 5 (top) showed the results of the generality analysis for the 2005 and 2015 editions of the competition. Fig. S19 shows the ACCs of the participants of the 2005 and 2015 editions, taking the score of the opponent as difficulty (if two or more opponents ended up with the same score, they are considered together as "tasks" of the same difficulty). This is why we see values (grey circles) on 1 (wins), 0.5 (draws), 0 (losses), but also some other values. In the end, if we have all possible pairwise matches, we have a difficulty scale that goes between 0 (the opponent has lost all matches) and the total number of participants (the opponent has won all matches). After this transformation, we can apply generality analysis (GA) as usual.

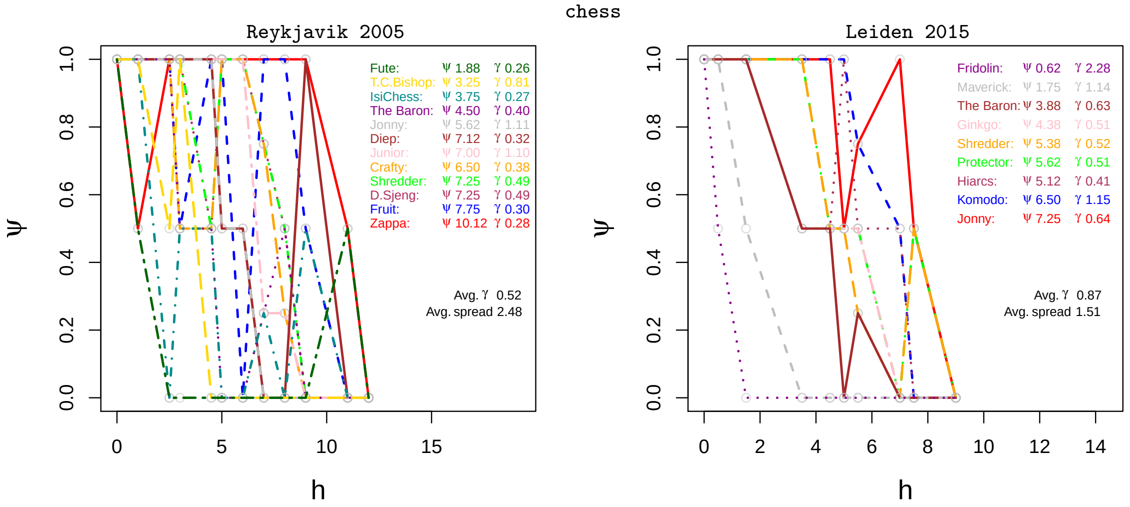

Figure S19: ACCs for all the participants in the World Computer Chess Championship using the final score of the opponent as difficulty. Data from https://www.game-ai-forum.org/icga-tournaments/game.php?id=1. Left: Reykjavik 2005 with 12 participants. The winner (Zappa) and the last one (Fute) won and lost all matches respectively except the one between them, which was surprisingly a draw. Right: Leiden 2015 with 9 participants. Here, no low-rank participant beat any high-rank participant, and draws were usually between participants with close scores. Accordingly, the average generality is higher in this case.

The plots show that both editions show good generality values, with the 2015 edition being actually more general (although in this case the correlation between generality and capability is negative). We can also conclude that the degree of transitivity is high, in light to the generality values. Note that in adversarial settings like

chess, agent transitivity and task transitivity are two sides of the same coin, as tasks are opponents, which are also participants (if a participant A beats B and B beats C, how likely is it that A beats C?).

A.7 ALE (with Aref and Rnk transformations)

ALE [19] is a collection of old Atari video games that has become very popular for the evaluation of general-purpose reinforcement learning algorithms interacting with the game at the raw pixel level (screen shots) [20]. These video games have no difficulty metric and each game has different score magnitudes. In order to see progress in these games, AI researchers have compared their results against humans, using some recorded data about human performance on all games.

This is exactly the ARef case in Table 1, where our *RefAgent* would be humans. We start with a response matrix R with $M = 24$ subjects (23 AI systems plus the human reference) and $N = 45$ games. The transformation of R is straightforward in this case. We simply discretise results to 0 or 1 depending on whether the particular cell in the matrix is greater or lower than the corresponding result for a human for that game. Then, in order to derive a metric of difficulty for each game, we take a populational approach, the percentage of AI systems that are below the human score. The higher this proportion, the more difficult the game is. After this transformation, we can apply generality analysis (GA).

When we do the generality analysis, we can see the measures for the 23 systems and the human reference (human.noop) in Fig. 5 (second row, left). In this case, as the human reference is always equal to or larger than itself, we used the convention of setting a score of 0.5 for humans (they are borderline with the human limit), so we see humans (human.noop) more or less in the middle (and on the constant isometric, as humans are borderline with humans independently of the difficulty of the problem). Overall, for all other agents generality is quite high, and R2D2 (the best AI system for this benchmark) only seems to fail (be worse than the human reference) on one game over the 45 games included here, which is also very difficult.

But can we analyse the data without a human reference? Yes, but the transformation is more convoluted. Following case Rnk in Table 1, we use a value of $c = 100$, getting the result in Fig. 5 (second row, right). We see that the new right plot is very similar to the left plot, albeit using two different transformations. But now, as humans have been considered as any other agent, their place in the plot, and its generality, is more meaningful. Actually, we see that the capability is more or less on average (as in the left plot), but generality is very low in comparison. In comparison with the population of AI agents, humans do very well on some games and poorly on others, being less general than most of the AI agents.

Overall, we can trace the dates when the AI agents were introduced for any of the plots of Fig. 5 (second row). In this way we could analyse the temporal evolution of AI systems in both generality and capability, from the early systems being worse than humans for many games (usually on the left of the plots) to current systems, being better than humans for almost all games (more on the right). Figures S20 and Fig. S21 provide the names of the supplementary files where this evolution is animated.

The animation for the temporal evolution (by month) of AI systems for the ALE benchmark<br>in terms of capability and generality can be found in the supplementary file:<br>`ale.refHUMAN.capability-vs-spread.evolution.months.mp4`

Figure S20: Temporal evolution (by month) of AI systems for the ALE benchmark.

The animation for the temporal evolution (by year) of AI systems for the ALE benchmark<br>in terms of capability and generality can be found in the supplementary file:<br>`ale.refHUMAN.capability-vs-spread.evolution.years.mp4`

Figure S21: Temporal evolution (by year) of AI systems for the ALE benchmark.

The evolution videos show that the improvement has not always been incremental in terms of capability and generality, until reaching the final point (R2D2), better than humans for all but one game, with high generality and capability. However, it is important to have in mind that here the algorithm is retrained for each game. In other words, it is not the same system solving all these games, but the same algorithm (after specific training) solving each of them separately, with no memory or transfer between the games. Of course, humans need some training in each of the games, but the number of required episodes in not comparable, mostly because they do transfer a cross-generalisation. Because of these important differences, significant research in AI is taking place in systems that can transfer and change between games with little retraining.

A.8 GVGAI video games (with Rnk transformation)

In the case of the Atari games above, we see that the positions in terms of generality and capability are similar with the Aref and Rnk approaches, which gives us support for the use of this methodology in cases where we do not have a reference agent, such as GVGAI. General video game AI (GVGAI) is another popular video game competition in AI [21, 22], with a benchmark that comprises a large number of real-time 2D grid games. In this case, we use 23 systems from a competition in which they confronted 49 games [23]. Each game has 5 variations. We will explore the case where we consider each variation as an independent game (so having 245 items) and a grouped version where we aggregate the five variations of each game into one (so having 49 items).

Before doing the transformation and the generality analysis, we performed factor analysis. In the ungrouped case, there were some constant columns, leading to problems in the correlations, but in the grouped case, the mean of the FA loadings for one factor was 0.60 and the accounted variance was 0.43, which suggests the existence of a possible general factor in this population.

Now we perform the Rnk transformation (see Table 1), deriving difficulty as the ranks of the AI systems from which we have collected results, using a generation of new columns with $c = 100$. The capabilities and spreads are shown in Fig. 5 (third row, left ungrouped, right grouped). What we see is that the systems are quite general (which is consistent to what we got from FA), but they distribute in a quite thin flat band, where the agents with middle capabilities are more general (at least according to normalised generality) than those with low or high capability. The results for the ungrouped and grouped cases are very similar, with a little bit more dispersion on the right plot (probably due to more robustness on the estimation). In the right plot we can see more clearly that with about the same capability, MnMCTS is more general than TeamTopBug. Both use different approaches, but the latter may have a tendency to specialisation for some types of games (independently of its difficulty).

A.9 Physical cognition tasks (with Rnk transformation)

We now explore the results from [24], an empirical analysis of orangutans (Pongo abelii and Pongo pygmaeus) on five physical cognition tasks: Box Task, Detour Reaching, TubeTrap Task, Honey Tool Task and Reversal Learning. The study analyses "general cognitive abilities", so it is interesting to see what the generality measure can show in this case.

There are 53 orangutans and 5 items. We start with factor analysis, where the mean of the FA loadings is 0.36 and the accounted proportion of the variance is 0.19. This might be partially explained by the low number of items (5), but in any case we cannot conclude (or rule out) the existence of a general factor.

Let us try generality analysis instead. Again, we use the Rnk transformation with $c = 100$ (this generates 500 columns, giving a sufficient resolution of difficulties). The capabilities and spreads are shown in Fig. 5 (bottom row, left). We see important differences in capability and generality for the 53 orangutans (shown with their names in grey). Some of them, such as Bella, are more general than other more capable ones, such as Julius. The aggregates (shown in coloured symbols) are very similar for different groups (species, age, etc.), where only the background results (bkg-*) deviate from their centre (but their subsamples are smaller too). Finally, we separated the results of low-capability orangutans and high-capability orangutans, with average generalities of 0.73 and 0.75 respectively. Again, no SLODR in the context of GA appears in this case.

A.10 The Primate Cognition Test Battery (with DRef transformation)

Finally, we are going to analyse an increasingly popular battery for the evaluation of a wide range of cognitive capabilities. Initially introduced for primates in [25], it has also been used or adapted for non-primate animals. We will use the original data from [25], which evaluates human infants, chimpanzees and orangutans. We process the results aggregated into six categories: "Space", "Quantities", "Causality", "Social Learning", "Communication" and "Theory of Mind". In this case, we compare populations and not individuals, but interestingly we have the standard deviation of the results for each species in the six categories. So in this case we are going to use a different transformation, DRef, where we reconstruct a distribution of results for humans. In order to do this, we use the mean and standard deviation for each category to derive the quantiles using a beta distribution (more appropriate than a normal distribution as the values are between 0 and 1). With this, we generate $c = 100$ new columns for each category, as we did in the Rnk approach, but in this case looking at the quantiles of the reference distribution rather than the ranks. Also differently, we derive the difficulty as the quantiles of the distribution. In other words, results and difficulties are transformed using the human *distribution* as a reference.

What we see in Fig. 5 (bottom row, right) illustrates that the generality of humans is higher than chimpanzees (and their *normalised* generality also higher than orangutans). Of course we are using humans as a reference here, but the location of points is basically given by non-human apes not being good at the non-physical categories, while humans being consistently good for the six categories. While the data in this case is very

aggregated to allow for a more refined analysis, the picture of Fig. 5 (bottom row, right) is sufficiently simple and clear to understand how these apes compare in terms of capability and generality (for the PCTB).

B Psychometric interpretation: generality, the *g* factor, SLODR and the *c* factor

In this section we will analyse the interpretation of the notion of generality in the context of the science and literature of human intelligence and psychometrics [26]. We will first flesh out the clear connections and inspirations, and then we will explore some other more profound implications.

B.1 Related metrics and models: person-fit, Guttman scales, reliability and variable-θ models

The connection between ability and difficulty is has been frequently vindicated as "the foundation of [...] measures of mental ability" [27]. Accordingly, psychometricians will find the curves and indicators familiar in some ways. The use of two parameters, difficulty for items, and ability for subjects is common in classical test theory and especially in item response theory [28, 29]. Also, plotting the performance, or the probability of correct response, against ability on the x-axis leads to the item characteristic curves. Similarly, plotting this against difficulty on the x-axis leads to subject or person response curves [30, 31]. It is important to note, however, that in IRT, both ability (usually denoted by θ) and difficulty (usually denoted by b in logistic models) are latent factors, which are estimated by making several assumptions: "1) local independence, 2) unidimensionality, and 3) a specified shape for the item characteristic curve" [31]. The shape is determined by a model, which is usually a decreasing monotonic function on $b - \theta$, such as a logistic function. Then the parameters are estimated from a response matrix $r_{j,i}$.

In our case, we are not considering a measurement problem (yet), and we are not (necessarily) plotting latent variables. Difficulty could be a notion derived from the items themselves, and capability —the metric we use instead of the psychometric 'ability'— is not the parameter of any function. Actually, we define capability —and we use a different term on purpose— as an area, and not the location of the steepest point of any curve. For models that are symmetric at $y = 0.5$, such as 1PL or 2PL logistic models, the area equals this location. However, for irregular curves not following a model at all, it is the area what is really meaningful. Also, we are not plotting correct response for each item, but aggregated or expected response per difficulty.

The key question about the assumptions in IRT is that even if some models allow for a discrimination parameter for the items, so that that the correlation between correct response and ability for all items is relaxed (it might even be negative), this is not usually the case for ability. For many models, IRT is actually assuming a strong (negative) correlation between correct response and difficulty for all agents. Note, by the way, that a fully steplike ACC does not maximise (negative) correlation. In this extreme case, correlation is actually -0.866 if the step is in the middle of the range of abilities, but it may even be 0 in the limit if the difficulty is not bounded. The models (including the variable-θ ability models we will mention below) consider that a subject being better at difficult items than easy items is an aberration, mostly because the models and estimations are done in such a way that this is assumed not to happen (or should just show a bad fit to the model).

This has actually led to a myriad of person-fit metrics [32], which is a way of analysing subjects at the individual level. This aims at identifying cases such as "low-ability examinees who copy answers to several difficult items from a much more able neighbor and very high-ability examinees fluent in another language but not yet fluent in English, who misunderstand the wording of several relatively easy questions" [31]. But in the end, all this is about whether the observed curve matches the expected curve. This was not meant to measure generality.

As there are so many person-fit metrics, some of them are relatively similar to Γ, as defined in this paper. Especially relevant are those that compare the person response curve with a Guttman conformal curve, which is a curve that is right for the first r items of lowest difficulty and wrong for the rest (a step function). In this setting, the closest metric seems to be the *norm conformity index* [33], which basically counts how many ranking mismatches there are between a Guttman curve and an observed curve. Another very related metric is the disagreement index [34], where the agreement index (the sum of the results multiplied by the difficulty index for all items) is compared with the score of the Guttman conformal curve with the same *number of correct* responses (NC score). Since all these metrics are ordinal, and convert the difficulty of the items to ranks (index), the correspondence to Γ is only direct when we have a uniform distribution of items per difficulty. In other words, all these metrics take all instances as equally valuable —the NC score is the number of counts, the number of correct responses—, while the agent characteristic curves shown in Fig. S30 sum with the assumption of difficulties being uniformly distributed. So, if there are more items for some difficulty values than others, the count (the NC score) and the area (the capability) would be different. This is intentional, as we are not interested in a capability according to a set of items, but according to different levels of difficulty. Actually, for

many difficulties, the number of items might be infinite. Assuming an uneven number of items per difficulty does not have more support than assuming them uniform. Capability is invariant to this.

Still, because many of these metrics take the step function as a reference, it is important to look at the Guttman scale or, more precisely, the deterministic model [35, 36], which can be considered a precursor of IRT. A deterministic model just captures the item response curve as a step, i.e., the probability of correct response is 0 for values below the ability θ and elsewhere. This model produces agent response curves that are also a step —the Guttman conformal curves— and, hence, they would have infinite generality. Several properties derive when items (and hence agents) follow this model. In particular, task transitivity and agent transitivity are true under this model, as shown in §E.2 (properties #6 and #7).

The Guttman scale assumes monotonicity (higher probability of response for higher ability), but there are many other models (some non-parametric [37, 38] and some parametric [28, 29]) assuming this. The Guttman model has been used in cases where solving one item means all items of lower levels of difficulty have to be solved as well. For instance, in arithmetic, at the lowest level of difficulty one might have addition and then at the next level we can have multiplication. Arguably, one cannot do any multiplications without knowing addition (although there are very simple cases such as multiplications by zero or by one that do not require any addition in the process). In general, the Guttman model does not hold for practical sets of items, and it is mostly used because of its simplicity.

It is important, hence, to say that our notion of generality is not assuming the Guttman model for items (or a non-ordinal version of it) or a conformal Guttman curve, but just measuring how far the expected responses of an agent are from that theoretical situation.

Finally, there is a clear resemblance of the notion of generality with "person reliability", as introduced by Lumsden [39]. The notion of reliability wants to capture "tremor effects", i.e., each person has a variability on its ability θ. Actually, Lumsden models this reliability with a normal distribution and then the agent characteristic curve turns out to be its CDF. For constant-θ IRT models, like the traditional logistic models or the Guttman model, the theoretical agent characteristic curve has the same slope for all respondents. This changes for variable-θ IRT models, where reliability is introduced as an extra parameter (sometimes sacrificing the discrimination parameter, depending on the degrees of freedom).

In general, without considering any particular model, an agent can get constant θ, with no reliability issues at all, and still have a flat curve. Simply, the agent is consistently bad at easy problems, like the two top plots on Fig. S30. It is only when we limit ourselves to some particular models that we can understand the slope of the curve as a reliability. In other words, variable-θ models assume "that the person trait level varies during test administration" [40]. By using expected values and thresholds transforming them into accomplishment values we exclude the reliability component and we focus exclusively on generality. Tremor effects can also be discounted as they should appear for all difficulty levels.

Perhaps because of this confusion between reliability and generality, the agent reliability metrics are not as widespread as the person-fit metrics commonly used for constant-θ IRT models. But we have to be careful about person-fit: "From a constant-θ point of view, person reliability can be considered as a source of misfit or overfit at the individual level. Thus, the imprecise, highly unreliable respondent [...] will produce an almost random pattern that will be regarded as misfitting. At the other extreme, the highly reliable respondent is expected to produce a highly scalable response pattern that fits the stochastic model too well and that will be regarded as overfitting". Here, in contrast, with the individual metric of generality, we are not considering any model to fit. For generality we just examine the distribution of the expected responses in terms of difficulty.

Once the differences between generality and reliability are clarified at the conceptual level, we may be interested in the connections at the formulaic level. For instance, if we generate expected responses according to a normal distribution (like the middle right and the two bottom plots in Fig. S30), with a standard deviation σ we have the following[2]:

Proposition 1. *Assuming a normal distribution on capability, with standard deviation σ, the slope of the ACC will be $-\frac{1}{\sigma\sqrt{2\pi}}$.*

Less trivially, we can show the following lemma and proposition:

Lemma 2. *Assuming a normal distribution on capability, with mean μ and standard deviation σ, such that the location is sufficiently beyond 0 to have negligible mass below 0 (i.e., $\frac{\mu}{\sigma} \gg 0$), we have that $M_j = \frac{\sigma^2+\mu^2}{2}$.*

Proposition 3. *With the same assumptions as lemma 2, we have that spread $S_j = \sigma$ and $\Gamma = \frac{1}{\sigma}$.*

The definition of person reliability was just $1/\sigma$ [40], so we see the equivalence between reliability and generality if the agent had an ACC that were complementary of a normal CDF. However, in our case we do not understand σ as the standard deviation of capability or its measurement and there is no special reason why this should be normal. A different interpretation appears if we consider the estimation of difficulty to have an

[2]Proofs in §F.

error that is normally distributed, with a variance that is constant for all difficulties. We will come back to this when we discuss possible ways of estimating the difficulty function.

B.2 From individual generality to populational generality: manifolds and the g factor

As we mentioned in the paper, Charles Spearman found an important phenomenon; when he analysed a set of different tests taken by the same population, and calculated the correlations between tasks[3], he found a positive average correlation ($\bar{\rho} \gg 0$). A person obtaining good results on a test usually obtained good results on the others. This phenomenon was known as the 'positive manifold' [41, 42], and was stronger the more culture-fair and abstract the tests were. The correlation does not emanate from the tests only, but depends on the agents in the population. Despite this dependence on the population, the positive manifold has appeared repeatedly for different human populations and different sets of tests, provided they are not too linked to particular cultural or educational backgrounds. Spearman introduced a rudimentary factor analysis to extract a dominant *latent factor*, which he called the g factor, explaining a significant part of the subjects' variance. He called it the g factor. Since then, this factor has been found systematically in different populations [43, 44] and has predictive value about many facets of human life, from academic performance to (lack of) religiosity. The controversy appears when g is associated with general intelligence, and is said to underlie all other factors and facets of intelligence. In other words, it is not g which has been called into question, but its interpretation.

Note that the theory behind g allows psychometricians to estimate how much of this factor an individual has, the g score, giving us a latent factor that can characterise and distinguish individuals. But this score is not generality, but (general) ability. For two different people with the same g score, we could have that one person achieves good results for many cognitive tests consistently but another person may get a more uneven performance for the same set. In other words, the predictability of g scores is analysed globally, but still some individuals may be less predictable than others. One possible reason may be reliability[4], but another reason is simply that some individuals are less general than others. In the end, g was the result of observing a general factor emerging from human performance on a range of tests. But where does this general factor come from in the first place? Is it a necessary result if the individuals are really general? This new question is what we try to explore below.

Let us first analyse the situation where the positive manifold is extreme, with a mean correlation of 1. This means that all columns in the response matrix $r_{j,i}$ have a correlation of 1. Now let us assume that all columns are normalised (same mean and standard deviation). The only possible situation for this to happen is that all columns are equal, $\forall j, i \; r_{j,i} = r_{j,1}$. So clearly, for each individual we have zero variance in the tests. But this zero variance between numeric values for a test does not mean infinite generality (which must be calculated from an ACC). Consequently, we cannot relate this to generality as we do not know the instances in each test and their difficulty. But let us assume that we convert each response in the response matrix into a step ACC with capability at the $r_{j,i}$, or in other words, we assume that $\psi_j(h) = 1$ iff $r_{j,i} = r_{j,1} \geqslant h$, and 0 otherwise, and merge them into a single ACC for each agent j as all of them are equal. Clearly we have that $\Psi_j = r_{j,1}$ as all of them are steps by definition, also having $\mathit{\Gamma}_j = \infty$. In brief, *only under this "thresholding" approach that constructs step ACCs for a range of tasks*, a mean correlation of 1 would imply infinite generality. Actually, this transformation approach is analysed in §F.4, where we get an interesting and straightforward connection between generality and row variance in the general case (no extreme manifold).

Secondly, let us now analyse the situation where all agents have maximum generality. Without loss of generality, we can consider that the rows of the response matrix $R_{M\times N}$ are ordered by increasing capability (the columns may also be ordered by increasing difficulty but this is irrelevant here). The values for each and every column in the response matrix $r_{j,i}$ would be of the form 0^p1^q, with $p + q = M$, i.e., the item response curves would follow a Guttman model. If $p > 0$ and $q > 0$ the correlations will be well defined and will be strictly greater than 0 and there will be a positive manifold. Depending on the distributions of capabilities and difficulties the magnitude of the average correlations will vary. For instance, it is easy to see that if we consider a normal distribution of difficulties and an equal normal distribution of capabilities, the mean correlations will be around 0.47, which is a very important positive manifold. In this situation, we see that individual generality implies a positive manifold. We do not even need to do factor analysis to check whether individual generality extends as a populational generality. As Guttman points out, a notion of *populational* generality can just be defined "as having all correlations positive or zero", without the need of "a common factor" [45].

Spearman, and most of the literature after him, analysed the positive manifold for tests instead of items. Tests group a number of items that are considered to be related (e.g., a maths test) and include a range of

[3] Pairwise correlation $\rho_{r,s}$ is calculated for each pair of tasks μ_r and μ_s, columnwise, on a result matrix such as the one shown in Fig. 1 (top) —but usually with many more rows. Then, all pairwise correlations are averaged into $\bar{\rho}$.

[4] With *g=1* we can still have that each agent fails a different percentage of the times, but in a completely random way. Actually, by taking a perfect agent and introducing different levels of systematic noise to form a population, one would get perfect g. This is not generality.

difficulties so that we get diversity of results for the test according to the population it is going to be applied to. So let us consider that items or tasks μ_i are grouped into tests τ_k. Now we can construct a new response matrix where columns k are tests and rows are agents j. We can analyse that by aggregating items into tests, mean correlations may get much higher under different scenarios.

For instance, let us consider both item difficulties and capabilities following the same normal distribution (sufficiently far from 0 so that there is negligible mass below 0). In this case, we have that if we group the items randomly, we can get mean Pearson correlations above 0.99. If the new groups preserve the item difficulty distributions, for each agent π_j we will have exactly the same results for all tests on expectation. As the agents have different capabilities, we will have a mean Spearman correlation equal to 1 and, if the distributions are normal, a very high mean Pearson correlation.

Other similar connections can be obtained with some other distributions, assuming that each test preserves a range of difficulties such that it ensures the differences in capabilities to be represented per each test. This is actually a very natural condition for a test to be informative (if all respondents got similar values for the test, then the test would not very informative). As a result, if this variance is preserved, the only strong sufficient condition for a high manifold to appear is individual generality[5].

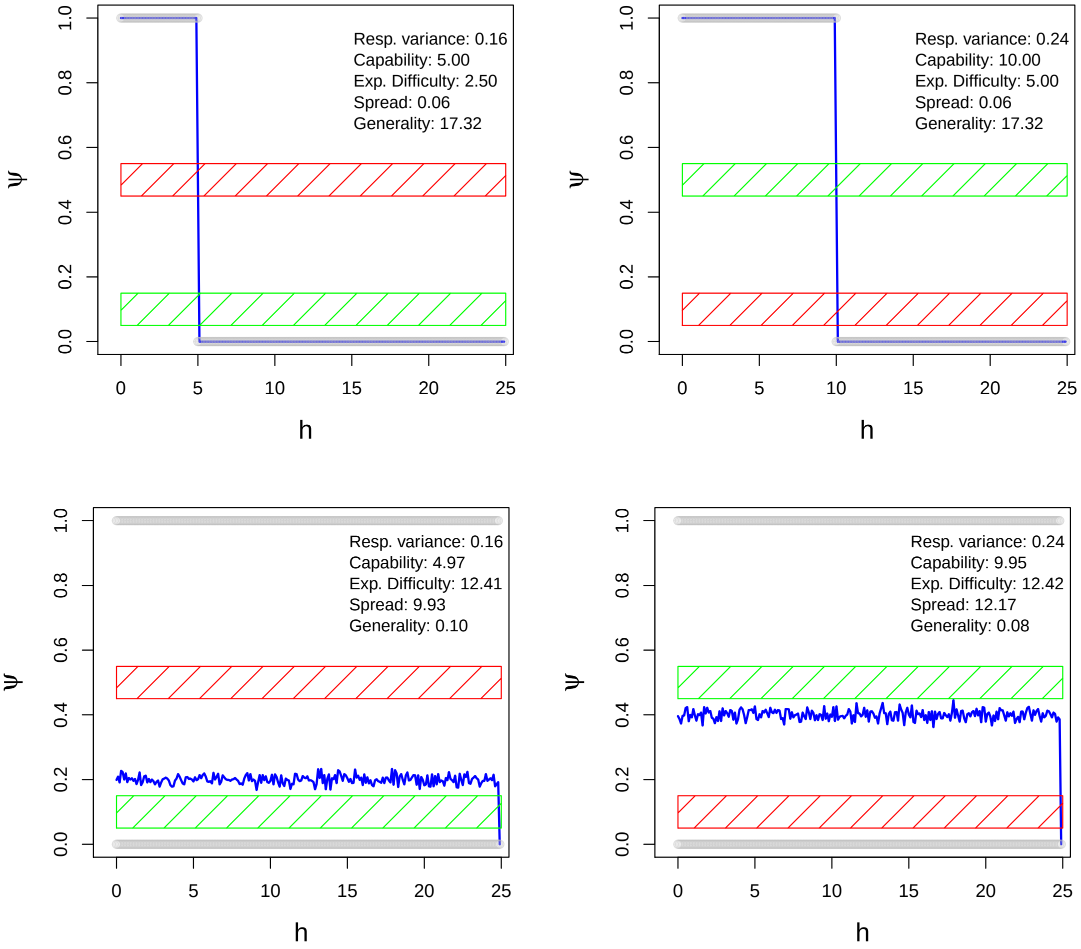

Figure S22: Four agent characteristic curves, with the bands showing two possible tests (red and green) grouping subsets of tasks. Top: We see two maximally general agents. Independently of how the groups are made for the two tests, provided the same range of difficulties is covered, the curves for each subset would be the same and so the effect on the populational generality. Bottom: groups can be made in such a way that the green test gets all positives for the bottom left plot but all negatives for the bottom right plot, and the opposite for the red test. As a result, the manifold might even be negative. Compare with Fig. 1 (bottom).

[5]This sufficiency condition does not exclude that populational generality could have been obtained by other means, with all agents with different capabilities but flat ACCs, as the one on Fig. S30 (top left). This situation would actually require fewer conditions on the distribution of difficulties (actually difficulties would not play a role for these curves up to the point where the flat curve stops) but will necessarily require a random sample per difficulty (one could even get negative manifolds if instances are chosen on purpose to do so). Ultimately, dominance between ACCs (not only different capabilities) would be a more refined condition for this.

Only the maximally general ACCs can ensure that for every possible partition or sampling of instances, provided the range/distribution of difficulties is kept, the manifold is created, since the capabilities are preserved for each subtest. This is illustrated at the top of Fig. S22 in contrast with the bottom of the same figure. If individuals have low generality, choosing sets where a difficulty range is preserved is less important, and the positive manifold could still appear if the tests are not splitting the items by pockets of speciality. In other cases, the manifold might even be negative. On the other hand, if individuals have high generality, any partition of items into tests provided the range of difficulties is preserved would lead to high positive manifolds. In any case, negative manifolds would never appear.

Negative manifolds are very rare in the literature of human intelligence. Also, having sets of cognitive items for which difficulty does not play a role seems very unnatural. But still, the evidence might be compatible with some moderate degrees of generality or some individuals being more general than others. The plausibility (or necessity) of a particular scenario in light of a positive manifold will depend on a series of assumptions. Of course, the sufficiency direction is clear: if we are able to measure generality of the individuals in a population and we know how tests are formed, we can predict the manifold.

B.3 Spearman's Law of Diminishing Returns (SLODR) and individual generality

There is another source of evidence that can help us with the analysis of the plausibility of individual generality in light of a positive manifold. This evidence was also first gathered by Spearman. He calculated the correlations and g factors for the results of two different groups over the same tests: a group of humans A with normal abilities and a group B with low abilities. The mean correlation for group A was 0.47 while the mean correlation for group B was 0.78. The *proportion of the variance* explained by g for the low-ability group (scoring worse) was much higher than for the normal-ability group (scoring better). The more intelligent a population is, the less variability is explained by g. Spearman associated this with a possible saturation of g for most able groups, such as the *law of diminishing returns* in economics, and since then it became known as Spearman's Law of Diminishing Returns (SLODR). The finding has been replicated in many different experimental settings since then [46, 47, 48].

Spearman argued the items that were only solved by the most able individuals required the combination of many skills, and g would play a smaller role. Several other explanations have been introduced, such as [46] and [47]. These explanations have been contested but the very experimental evidence itself has been put into question. One explanation describes the phenomenon as an artefact of the way items are selected, choosing narrow ranges of difficulty. Basically, there are no sufficiently challenging items, and very able individuals do not require to use general intelligence, as they can solve the problems more mechanically. Using the same tests for both groups, and a ceiling effect given by an insufficient range of difficulties would create the observed effect. Relatedly, Jensen [43, p. 587] discovered that the most able group showed lower variance than the less able group. Finally, when the more able group was asked to solve problems of higher difficulty, the SLODR vanished, and the more able group even showed higher correlations [49].

This observation is more consistent with individuals having generality, such that if the distribution of difficulties of items is not adjusted for the two subpopulations (or sufficiently wide to accommodate both), the items would be on the left of the step ACCs for many individuals of the more able group, so the correlations of the most able group would be smaller. Note that this would not appear for flat ACCs (with very low individual generality).

In other words, the SLODR, without adjusting the difficulties, would not appear if the individuals were not general at all. However, it appears if the individuals are highly general. And it is also easy to see that if we adjust the difficulties, so that the distributions are the same for both groups (and the relative distributions of abilities are the same), then we would have exactly the same manifold, so no diminishing or increasing returns.

Indeed, there is a pressure about resources when trying to achieve capability. This may make the ACCs more compact for higher capability, leading to more individual generality for the more able group. That would entail an augmenting return, as postulated with the so-called Universal Law of Augmenting Returns (ULOAR) [50]. We will return to these issues under an evolutionary framework (pressure of resources) and also under a computational framework, by looking at the invariance theorem and the stability of difficulty.

B.4 Individual generality, collective intelligence and the c factor

Finally, let us comment very briefly about collective intelligence. Collective intelligence appears in the natural world in many ways, as groups of individuals of the same species, the whole species taken as a whole and even in ecosystems with complex interactions between several species. Here we just limit our analysis to the view of collective intelligence as teams that have to work together to solve tasks, usually in quite controlled conditions [51, 52]. While maximum generality is optimal for an individual, it is not for groups [53]. If all the individuals in a group have maximum generality, the one with highest ability will dominate the rest. By agent transitivity (property #7), everything any other agent solves will be solved by the dominant agent. Consequently, and the

result of the group will be at most the result of the best agent in the group. With more specialised agents, there could be more options to exceed the results of the most capable individual. Of course, this depends on many assumptions about the dynamics of the groups, with the exact outcomes easier to derive when groups just combine their capabilities by voting or weighted voting (if confidence is used) [54, 55, 56]. In more realistic scenarios, redundancy may be needed in a group, and hence more generality or more overlap in the abilities of the components may be beneficial.

One straightforward way of considering the aggregation of several agents is to consider that each agent is answering a proportion of the questions, chosen randomly. In the particular case of having N agents with step ACCs (maximum generality) then we have the conditions of lemma 19 and we have average capability and spread equal to the standard deviation of the original capabilities. Randomly choosing from general agents with different capabilities basically creates a non-general composite agent.

Interestingly, the aggregation of several curves for which we do not know the shape could be understood as a normal distribution on the reliability of the capability, transformed into a sigmoidal cumulative density function for the ACC. Consequently, findings such as the c factor [51], could be re-analysed by looking at the individual generalities first, rather than looking at the individual g scores (or IQ scores).

C Evolutionary interpretation: generality and general intelligence in the animal kingdom

The study of intelligence in animals (including humans) usually distinguishes between domain-general and domain-specific kinds of cognition. Much debate has been held on the presence of degrees these have in humans and other non-human animals. This ultimately relates to discussions around modular views of the mind [57] versus developmental domain-general learning [58]. It is also common to analyse whether social species are associated with more domain-general cognition, and the so-called social hypothesis (see, e.g., [59, 60, 61, 62]).

The definitions of what is general and what is specialised also vary in the literature, but it is usually understood as coping with a wide range of cognitive tasks, or flexibility for changing cognitive demands in an unpredictable environment [63]. Note that this view is similar to the notion of generality we are discussing in this paper, except for the explicit use of difficulty. In our case, we say that an animal or a species is cognitively general if it is able to perform equally well on a wide range of problems up to a limited difficulty. This contrasts with specialised animals or species that display a hardwired fixed repertoire of domain-specific functionalities where they excel, but are unable to cope with even the simplest tasks beyond the repertoire.

C.1 The g and G factors and intelligence convergence in animal cognition

A data-driven approach to the issue of general intelligence in animal cognition has usually been conducted with population analyses performed on several non-human species [64]. Burkart et al. [65] provides the most comprehensive review to date of the study of the correlation manifold in non-human animals, both intra-species (denoted by g) and inter-species (denoted by G). The main conclusion is that "there is increasing evidence for g in nonhuman animals, particularly in mice and primates [...] At the interspecific level [...], studies of primates and birds provide a robust pattern consistent with G" [65]. Although some methodological caveats exist [66], the evidence is understood as these factors being stronger if they are able (on their own) to explain a high proportion of the variance of results for a battery of tests in a population (individuals in a species for g, or species averages in a multi-species analysis for G).

If we represent the performance of several individuals or species for several domains, as shown in Fig. S23, the evidence for g would be more in alignment with the plot in the middle, which shows that when one individual displays some performance in one domain the individual tends to display similar performance in the other domains. This is much in alignment with the early notions of general intelligence in humans and the positive manifold, as per our early motivation around Fig. 1 (bottom, especially if we rotate the plots) and what we have just discussed about Fig. S22.

So we are in a very similar situation to the human case. We cannot directly derive individual generality from these findings unless we postulate further assumptions, especially in terms of the difficulties used for the items in the domains. Of course, items are wisely chosen with the right difficulty such that there is variability of results to explain. This variability is basically what is been looked after (a factor that explains a great proportion of the variance). For instance, if we take Fig. S23 (left) and add more items of low difficulty to domain D_2 and D_4, we would simply even the results, as there would be more correct responses for all individuals in these domains. The contrary is also true: there are spurious ways, by adding further easy or hard instances of particular domains, to go from the middle plot to something that resembles the leftmost plot. This is one of the key reasons why ignoring difficulty and considering low variability (as seen in middle plot) as an indication of generality is wrong, especially when we think of the spurious ways of achieving it. Despite all these problems, it is not customary to perform a systematic analysis of difficulty (for instance, using cognitive demands for each item, or applying

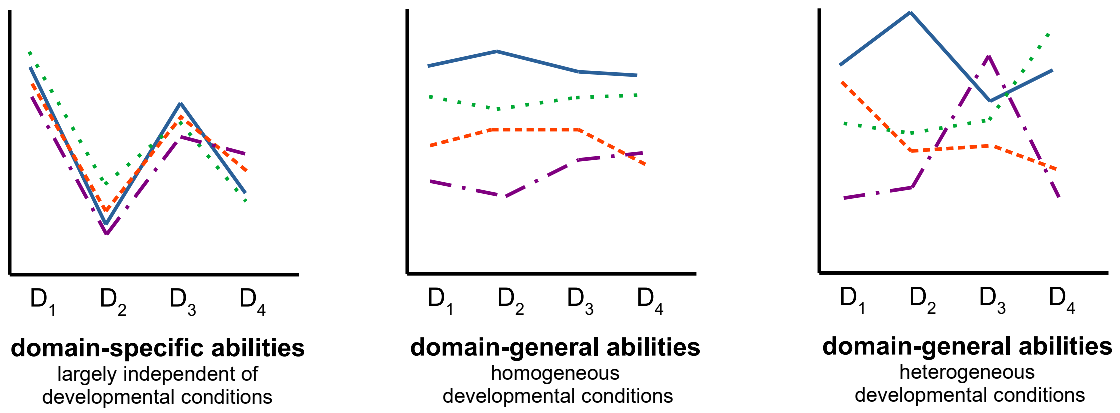

Figure S23: Three different possibilities for four individuals of a single species for four different domains D_1, D_2, D_3 and D_4. Left: The individuals behave better for some domains than others with very small differences between the individuals for each particular domain. Middle: The individuals behave equally well for all domains, but some individuals show higher performance than others, also in a consistent way. Right: At the species level, there seems to be no difference between domains, but individuals perform differently for some domains, either by individual differences or by "heterogeneous developmental conditions". [Adapted from [65, Fig.1].]

IRT). Also, in the first place, the identification of domains (such that they are actually diverse) is one of the fundamental methodological issues in the analysis of general intelligence in animals. "The issue of task selection is thus closely linked to the identification of domains in animal cognition, which in fact is part of the empirical question that needs to be addressed in intelligence research in animals in general, by using batteries as diverse as possible and statistical procedures that are a priori agnostic to the underlying factor structure" [65]. This is linked to the problem of dimensionality reduction, which has been highlighted by [66] as possibly being the cause of spurious results.

C.2 Cognitive resources and generality

In animal cognition, the references to resources (cognitive demands) required for the tasks *in several domains* are usually part of the discussions. Burkart et al. [65], for instance, set the question around how much extra neural tissue is needed, taking into account that domain-specific cognitive adaptations may require much less additional expensive brain tissue [67] than domain-general cognitive ability, which is also less directly linked to fitness-relevant benefits. They face "the puzzle that domain-general cognitive ability apparently evolved in at least some lineages, or perhaps even in birds and mammals in general, even though its evolution has had to overcome more obstacles compared to the emergence of domain-specific cognitive adaptations" [65]. One possible theory that explains this puzzle is the *cognitive buffer hypothesis* [68], which states that this extra effort in domain-general cognitive processes in larger brains buffers animals against environmental variation, and pays off for a wider range of behavioural patterns given by innovation, learning and, most especially, cultural transmission [25, 67, 69, 70].

Evolution usually finds a trade-off between specialised functions and more general capabilities, according to the effort that has to be put in terms of evolutionary innovations and energy consumption of bigger brains on one hand and how expectable and regular the tasks that are faced by the species are in their environments. In particular, Del Giudice and Crespi [71] include a series of trade-offs over four key properties: performance, robustness, efficiency and flexibility. Clearly, performance, efficiency and flexibility are closely related to capability, difficulty and generality —although the connections are qualitative rather than quantitative, at the measurement level. The trade-offs between efficiency vs flexibility and performance vs. flexibility are both associated with the "generalist-specialist trade-off" . We can see this trade-off in Fig. S24, where we compare the gains and the efforts of a domain-general cognitive enhancement versus a domain-specific cognitive enhancement.

Of course, how meaningful these numbers are depend on how well we can estimate the effort for general solutions versus specialised solutions and how likely the specific tasks are versus all other tasks. Actually, Fig. S24 assumes that all tasks are equally likely (or, more precisely, all difficulties are equally likely). When some specific behaviours or domains are very likely in the environment of the species, then specialisation will of course pay off. It is when there is environmental unpredictability in that many tasks are similarly likely, that the pressure for more general intelligence takes us to the kind of increase like the violet band in the figure rather than the orange one. Actually, in an environment where most tasks change in a few generations we would

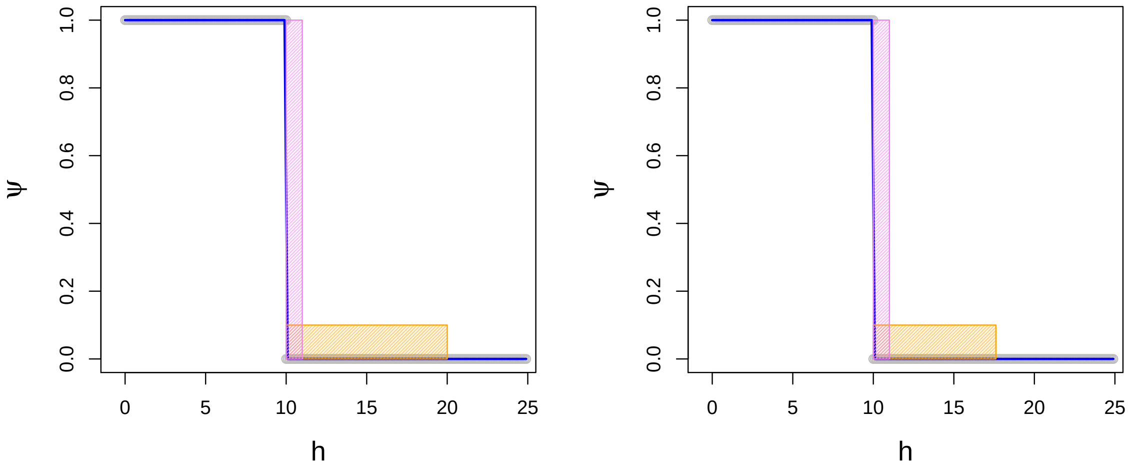

Figure S24: Using ACCs to represent two different ways in which the capability of a species can be enhanced, with a domain-general cognitive enhancement (vertical violet rectangle) or a domain-specific cognitive enhancement (horizontal orange rectangle). Left: both rectangles cover the same area (1×1 and 0.1×10), and hence increase capability in a similar amount, but the orange one has a higher expected difficulty, and hence effort ($M_j = \int_{10}^{11} h \cdot 1\, dh = 10.5$ vs $M_j = \int_{10}^{20} h \cdot 0.1\, dh = 15$, according to Eq. 2). Right: both rectangles imply the same extra effort ($M_j = \int_{10}^{11} h \cdot 1\, dh = 10.5$ vs $M_j = \int_{10}^{17.6} h \cdot 0.1\, dh \approx 10.5$, according to Eq. 2), but the specialised one (orange rectangle) now has a smaller area, and hence less increase in capability than the general one (violet rectangle).

have an ACC closer to the maximally compacted one and maximum generality (as given by the compactness property, #2), as this would be resource-optimal in order to obtain maximum capability (and maximum success if tasks are so unpredictable). Of course, many tasks requiring cognition, such as navigating and eating —but not foraging or hunting— might still be linked to a few particular specialised skills, as they are more constant in the evolutionary history of many species[6].

How difficult a domain-specific functionality is or how much effort it requires can be analysed in different ways. One first way is to look at the energy effort, by examining the involved neurological modules that are dedicated for that functionality, and map this with energy consumption. A second approach is to estimate evolutionary effort by looking at changes in DNA that make the functionality possible (from an ancestor that did not have it), contrasting with the ecological pressures and other similar functionalities. A third pathway is to identify these tasks and make them be learnt by systems that do have general capabilities, and estimate their difficulty from them. In this case, extreme care has to be made for many confounding factors. Finally, a fourth possibility is to determine the difficulty of tasks intrinsically (e.g., working memory requirements, pattern complexity, etc.).

C.3 Looking at evolutionary selective pressure through observable scores: capability and generality

Some less general species are able to solve very complex problems by specialisation that other more general species cannot do. We can finally look a this in terms of the two observable indexes we have introduced here: capability and generality. Plotting generality and capability against the level of social interaction (intra-specific and by diversity of predators), cultural inheritance, neural tissue mass, etc., with octopuses, hyenas, koalas, raccoons, primates and corvids, among other species, is expected to scatter points on very different locations. As a result, this could also help us see whether these traits are related, or whether there might be one-directional causalities. Both capability and generality are observable variables, the first is aggregated performance (the area under the ACC curve, eq. 1) and the second is a metric of how compact this performance is (how steplike it looks over difficulty, eq. 5).

Fig. S25 shows a simulation where 200 individuals are generated with random results on 200 items, and we see different selective pressures on the capability and the cognitive effort on the individuals: no pressure (top left), pressure on capability (top right), pressure on compactness (bottom left) and both (bottom right). As

[6] Judith Burkart (PC) suggested that a way of analysing adaptability could consist of giving the subjects control on a choice of tasks of different difficulty, with this information of that difficulty.

we see, the correlation between capability and generality increases when there is pressure on minimising effort (while keeping or maximising capability).

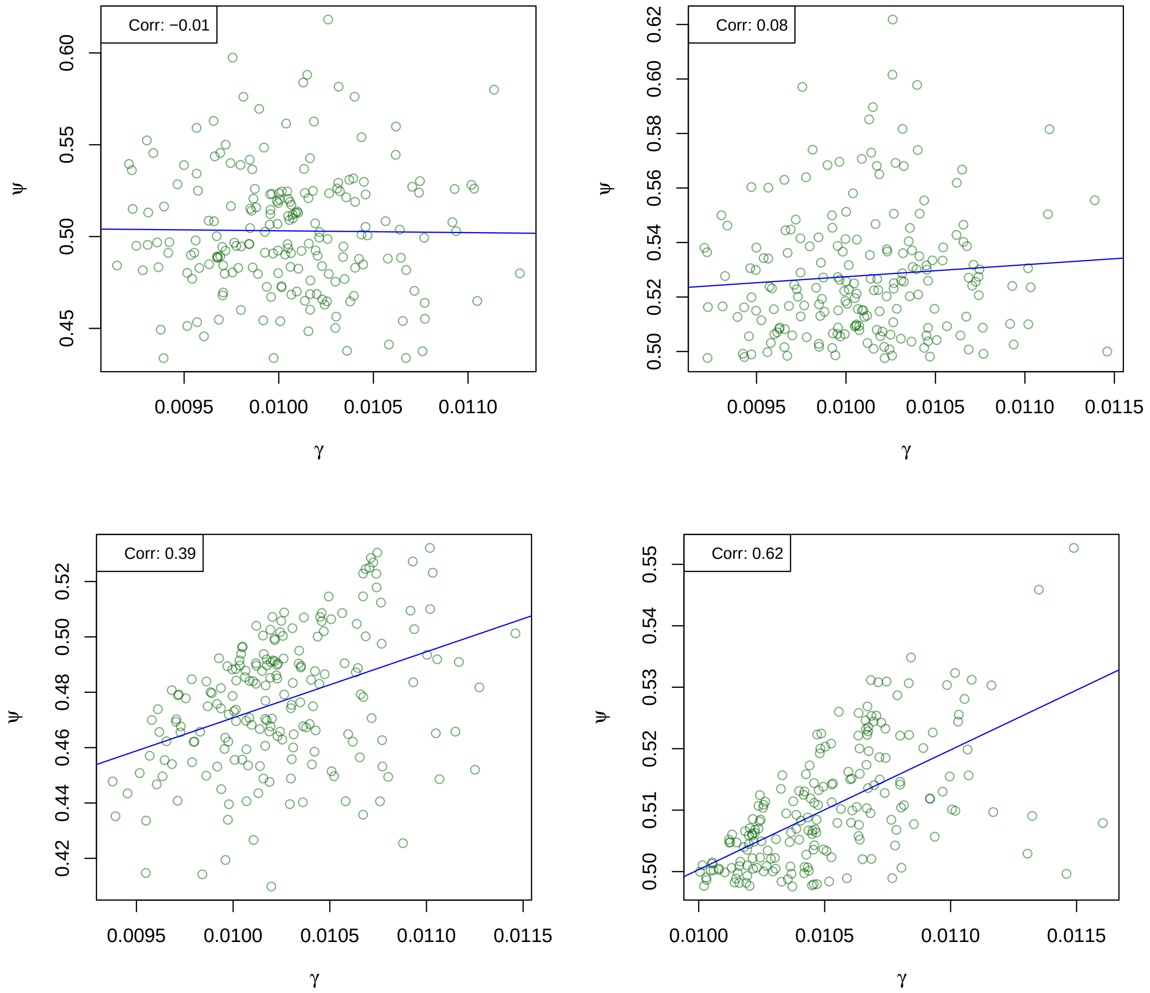

Figure S25: Distribution of capabilities and generalities of a simulation where 200 individuals are generated on random results on 200 items. The top left plot shows the original case with no selective pressure. This is not very interesting as all the ACCs are flat. The top right plot performs a selection per capability, where only those individuals with capability greater than or equal to 50% survive. The bottom left shows a selection by effort, where only those individuals that require less than 100% *over* the minimum possible effort for their capability (a maximally compacted ACC) survive. Finally the bottom right combines both selections at the same time.

Fig. S26 shows a similar simulation where, in addition, items have a uniform range of difficulty and success for the tasks is randomly proportional to the difficulty. In both figures the correlations can get very high since the pressure goes in the same direction: more capability and less effort. This is simply the result of the compactness property (#3). But again, it is important to notice that as generality and capability become more correlated (especially in humans) there is a tendency in confounding them, ending up talking about *general intelligence*, without knowing clearly whether the emphasis is on generality or capability.

The theory of general intelligence, the positive manifold and the g and G factors have all (in different degrees) raised bitter controversies. Setting aside the interpretation issues, one of the major arguments against these theories is that they might be considered statistical artefacts, produced as the result of making some choices on the items and test batteries. Some of these choices are critical: they should fit the population of individuals —not too easy, not too difficult, so there is variance to explain. In a very insightful way, Woodley of Menie et al. [72] break the species groupings by considering humans and chimpanzees together into a single population and then correct for these "ceiling or floor effects", by reducing the number of tests to those that have higher coefficients of variance. Fig. S27 shows the correlation of scores (d) and g loadings on the y-axis against different values of the variance produced by progressively selecting the tests with higher variance. Although not mentioned in [72], this analysis is of course closely related to the SLODR (and the alternative ULOAR

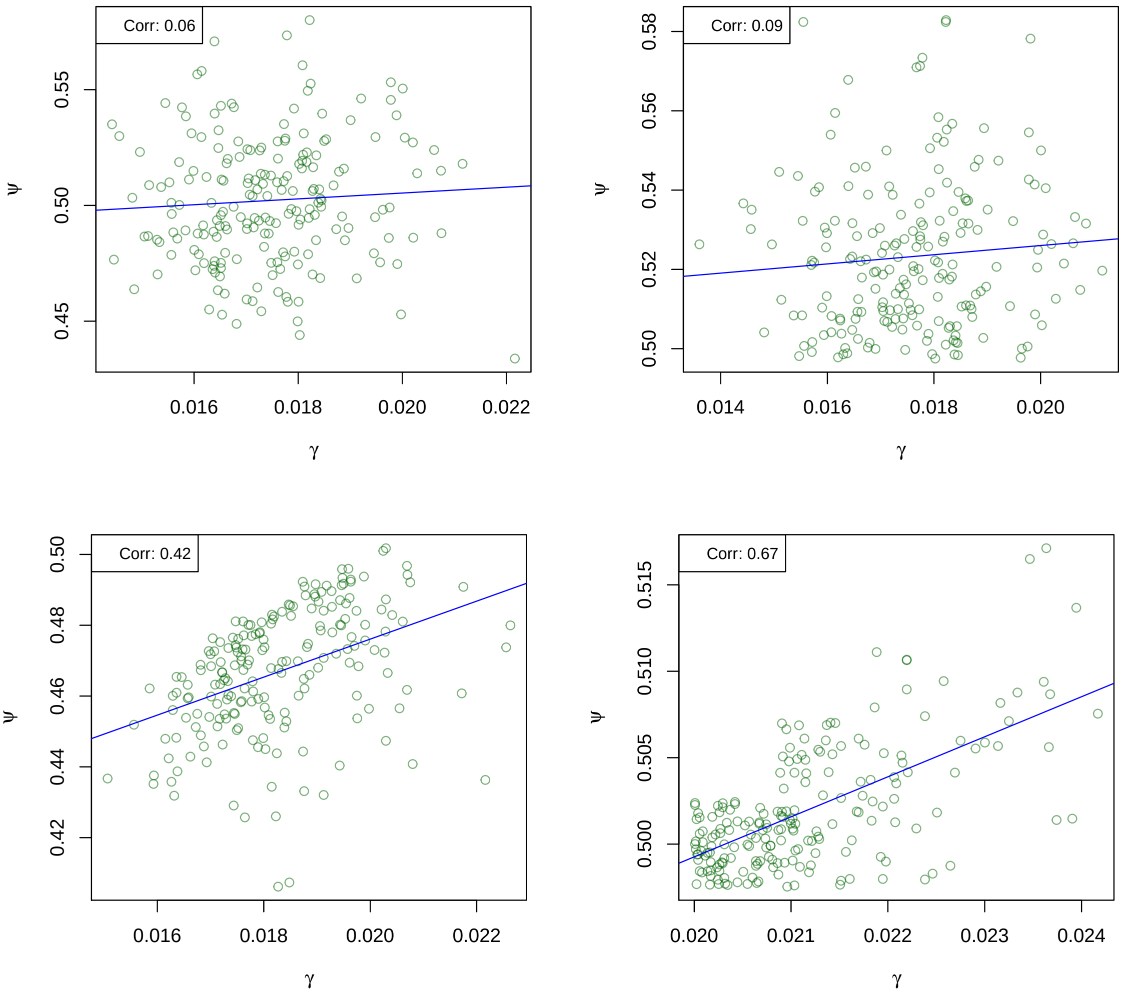

Figure S26: Same as Fig. S25 but with examples of different difficulties (ACCs are triangular originally). Top left: no selection. Top right: selection by capability. Bottom Left: selection by effort. Bottom Right: selection by effort and capability. For the two bottom plots, maximum effort set to 25% over the minimum possible effort (a maximally compacted ACC).

hypothesis) discussed in the previous section, where by adjusting the variance we can get that g and scores can grow together, as we see in Fig. S27.

By looking at generality, as an individual observable measure, we can simplify the analysis in many ways. First, the measure does not depend on a population of individuals, so we do not need to determine what is a sensible group or even consider them into species for the analysis. Second, because we can apply these metrics for a single individual, we do not need to obtain results for a large and unbiased sample of one species, which is usually very costly or problematic. Third, generality is algebraically independent from capability, and the correlation must come then from evolutionary or other kind of efficiency pressures applied to the individual or the species. For instance, actual plots like Figures S25 and S26 can be used as an alternative to Fig. S27, and done for individuals of many species together.

Another kind of criticism around the study of general intelligence is about whether "the positive manifold provides little or no constraint on the possible architectures of cognition" [73]. General intelligence may then originate from primary specialised modules being boosted by more general secondary modules (or evolved in this more compressed/abstract way for the economy of the brain), by a wide range of specialised modules that are switched on or off depending on the task at hand or by a truly general system helped by particular biases according to what environment demands are most frequent for a species. All this diversity of explanations could be extended to generality, as a high value of Γ can be obtained in many ways (but not as many as g, as we discussed in the previous section). As we will see in the following section, looking at individuals that have gone through no selective pressure, or a different engineering one (i.e., AI systems), can give us a wider theoretical and empirical scenario to exclude some interpretations of the existing findings. This can help calibrate new

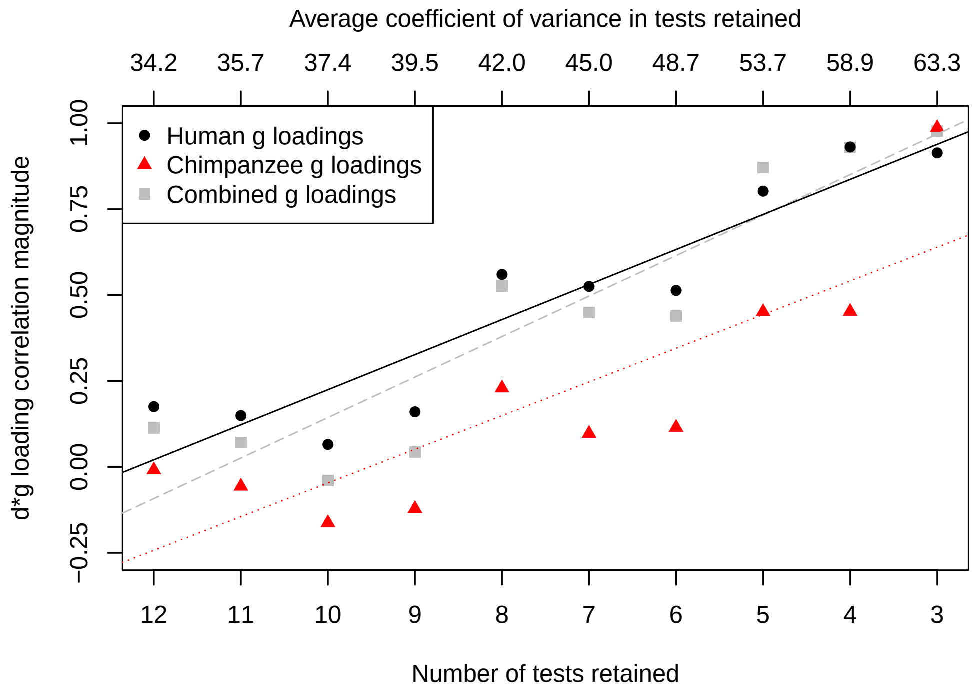

Figure S27: Correlations between task g loadings and the scores d on the y-axis as a function of the average coefficient of variance in the tests retained, choosing them by removing those with smallest variance first. Trends shown for chimpanzees, humans and a combined population. [Adapted from [72].]

research to come, which is looking at the values of Γ and Ψ in the animal kingdom.

D Computational interpretation: generality and artificial (general) intelligence

The debate along the spectrum between general intelligence and specialised (or narrow) intelligence has also pervaded artificial intelligence since its inception. The very early attempts were directed towards a General Problem Solver [74] and the goal of "generality" [75]. In the following decades, many of these programs failed to fully realise the complexity of intelligence, while other more narrow applications started to be successful.

In 1978, John McCarthy published a new version of his 1971 Turing Award Lecture on "Generality in Artificial Intelligence" [76], recognising that one of the major problems was that, if behaviour was represented by programs, these programs could only cover a finite set of domains or problems.

Of course, these were the times were machine learning was not a dominant paradigm in artificial intelligence. Nowadays, the use of machine learning techniques, coupled with sufficient data, allows systems to be adapted to different domains, using the same algorithm, which *generalises* the data. Generalisation is an intrinsic —if not definitional— part of learning. Learning is hence the way in which AI systems (and human and non-human animals) can adapt to unseen situations. In other words, when considering a large and diverse number of tasks, coding particular solutions for all of them is infeasible, and hence learning becomes the solution.

Consequently, it may seem that (machine) learning systems are then general by definition: give a learning system sufficient examples and it will learn any possible task. The goal of machine learning, and AI, would be to define this universal machine learning system. While this idea is still behind some of the narratives in machine learning and artificial intelligence, there is an important objection to this universal generality: *efficiency*. Some systems can potentially learn any function, given a sufficiently large number of examples. The question is how many examples, how much time and how large the model might be. The *answer* to —or *cause* of— this problem is known as *bias*. By embedding a particular bias for a learning algorithm, one can accelerate learning for some problems while making it harder for some other problems.

There are many ways of explicitly or implicitly introducing strong bias to a learning algorithm: specialised architectures, hyper-parameters, background knowledge, and the very algorithm itself. By using these particular biases, we can have AI systems that can solve particular pockets of problems: speech recognition, machine translation, robot navigation, medical diagnosis, face recognition, etc. Interestingly, by a shrewd use of more and more computing power, some of these algorithms are requiring less physical time (and occasionally fewer

examples) to learn these tasks, approaching, at least in some areas, the flexibility of some animals.

Still, there is a view that artificial intelligence does not produce general systems [77, 78]. Some prominent positions even deny the possibility of general intelligence at all, as Yann LeCun puts it: "There is no such thing as AGI. Intelligence is always specialized" [79]. While the same deep reinforcement learning can learn to play Go or Chess by just changing the rules [80], the *same* algorithm cannot learn to navigate a room and play poker [81, 82]. Of course, there are algorithms that can learn to navigate a room and have similar principles (and even shared modules underneath) to those playing poker. In either case there is a great amount of hyperparameter tuning, input and output transformation, and other changes to the architectures and the optimisation operators to make them work for a different domain. However, recent progress in deep reinforcement learning and transformers is producing systems where some sort of generality is undeniable [83, 84].

The area known as Artificial General Intelligence [85], where the *same* system should be able to solve a range of problems, is still seen as a counterpoint to a bevy of systems that are successful for more narrow domains, even if they are fuelled by machine learning, and built upon general principles looking for abstract representations.

Unfortunately, to the dismay of some members of the AGI community, the term AGI is now commonly used as synonym of ill-defined buzzwords such as human-level machine intelligence, human-level artificial intelligence or even superintelligence, without a proper analysis of what the 'G' in AGI actually means, and how it can be distinguished from mainstream AI [86].

D.1 Generality and all possible tasks

The reduction of AGI to anthropocentric views of intelligence has an intuitive appeal. We are interested in those tasks humans can solve. But which are these tasks? Or, more conspicuously, what are the tasks that humans —the hominids characterised by their general intelligence— cannot do? We can analyse this question and put the notion of generality to its limits by considering *all possible tasks*. One possible way of doing this is by defining the set of all computable tasks, with tasks being framed in a testing scenario, where agents can learn from experience. In other words, we can consider all possible learning tasks (see [87, 88, 89, 10, 90, 91] for different ways of doing this). Apart from the particular formulation and setting, the most relevant issue comes when we realise that as we now have a set of infinite tasks, we need a distribution over them, giving more weight to some over others.

Let us start with Solomonoff, who defined all possible sequential prediction tasks and an associated distribution, the algorithmic probability [87, 88]. The set of tasks is just defined by the problem of estimating the next bits of all the sequences that can be produced by a universal Turing machine UTM. While all sequences can be generated, their distribution (the algorithmic probability) depends on the reference UTM. In a way, this was an elegant way of representing the notion of bias in machine learning. Depending on the chosen UTM, some concepts will be easier to learn than others. Still, the great contribution by Solomonoff was that he showed that the same algorithm can be used for all UTMs (biases), and convergence can be obtained. A universal learning algorithm exists, it always works, but it will work more or less efficiently depending on the chosen bias, the reference UTM. In other words, each UTM assumes a prior about the world, and observations whose underlying pattern is simpler for the chosen UTM (smaller Kolmogorov complexity) are more likely than those observations with more complex patterns. Solomonoff integrates Occam's razor and Epicurus, as his theory considers the combination of all theories that are compatible with the evidence, weighted by their Kolmogorov complexity.

On the other extreme for the choice of a distribution we find the assumption that every possible problem's output is equally likely. In a sequential prediction problem this would be expecting all sequences to be equally likely or, in classification problems, to consider all combinations of inputs and outputs equally likely. This is referred to as "block uniformity" [92], a broader type of distribution than the uniform distribution. Block uniformity is one of the conditions for the famous no-free-lunch (NFL) theorems [93, 89, 94]. They show that, under these conditions, no learning method can be better than any other on average. A general-purpose learning system and hence the very notion of 'general intelligence' would be simply impossible [95]. Moreover, every agent would solve exactly the same number of tasks, so there would not be any variability in capability, effort and of course generality.

The NFL theorems are very relevant, because our observation that learning systems exist and work (in animals and computers) can only happen if the assumption is not true. This is the important corollary of the theorems. Apart from a pragmatic or ad absurdum rationale, there are more epistemological ones: choosing all perceptions as equally likely is difficult to reconcile with a world with physical laws and other agents around (plants, animals, conspecifics) that do not behave randomly. Actually, if we consider all these subsystems computable, Solomonoff's view is more natural, as the output of a UTM fed with random bits is not random. In other words, what we perceive, our world, is filtered through many machines —laws, mechanisms and agents—, making those patterns that are produced from systems with limited resources more likely.

From this view of all possible tasks, one can define a (universal) distribution according to the complexity of the generator of tasks, such as the complexity of the task description. However, one can also define the

distribution by looking at the complexity of the solution for the task, which can be seen as its *difficulty*. This way of weighting solutions by their difficulty is common in psychometrics, but was first introduced in the context of all (sequential) tasks in [10], recently extended to bidimensional grids [96]. When one goes from sequential tasks to interactive tasks (such as reinforcement learning [97, 98]), the difference between the smallest program that generates a task and the smallest program that solves the task becomes illuminating. Setting the distribution according to the former led to the notion of *universal intelligence* [99]. Setting the distribution according to the latter led to the notion of *policy-general intelligence*, assuming a uniform distribution over *solutions* for each task difficulty [100, 101, 91]. We can see some of these choices in Fig. S28.

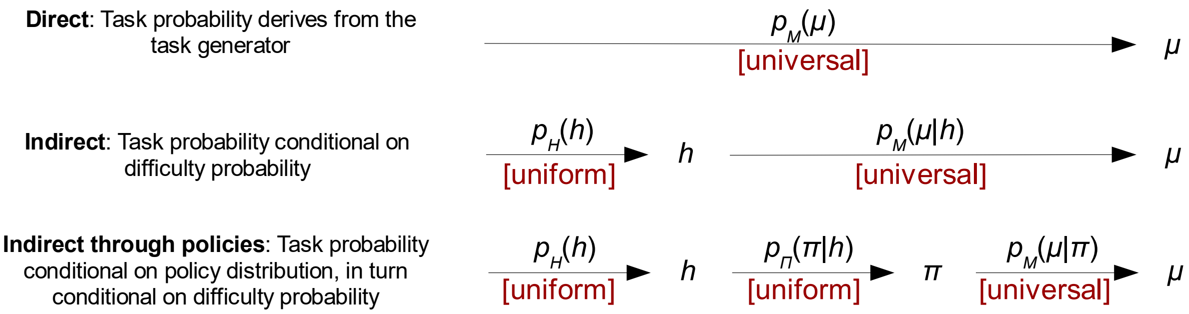

Figure S28: Different ways of generating tasks (or deriving their distribution). Top: the probability of a task is given by its generator. If the number of tasks is infinite, but countable, a uniform distribution is not a viable option, and a universal distribution must be used instead, making this equal to *universal intelligence* [99]. Middle: we first define a distribution of difficulties and then we define tasks according to that difficulty. In cases where the difficulty of a task can be derived from the definition of the task, this is a good option, as in [9, 10]. Bottom: again, we first define a distribution of difficulties and then we derive solutions matching that difficulty. Finally, tasks are generated according to the solution. This is actually an option when the *definition* of a task does not say much about the difficulty of the solution, such as interactive tasks, as used in [100, 101, 91]. Note that for the two bottom rows in the figure, if the difficulty distribution is uniform, the expected success on a random task drawn from the distribution is equal to the area under the ACC, which is capability, as for eq. 1. [Adapted from [91, Fig.9.7].]

D.2 The choice of diversity and difficulty

The important thing about a theoretical account of all possible tasks, and especially if we know how we generate them, is that we can control for two things that are crucial for generality: the diversity and the difficulty of the tasks. If we look at diversity first, the schema on the top of Fig. S28 makes it very hard to ensure that the set of tasks is going to be diverse, as we generate tasks according to a distribution on their definition, but not about their solutions. Besides, if the choice is a universal distribution as in [99], then the distribution is dominated by a few tasks, which cope most of its probability mass [102, 103]. For the schema in the middle of Fig. S28, we have at least some range of difficulties but, still, that does not ensure that the solutions might not all end up being of the same kind. Finally, it is the choice at the bottom of Fig. S28 that ensures diversity by the most entropic choice of a distribution per each difficulty (assuming the number of solutions per difficulty is finite). This choice is the uniform distribution.

For instance, Fig. S29 shows an ACC where instances have been generated according to the bottom schema in Fig. S28. If we consider all difficulties as equally likely, and assume the curve is 1 for $h < 7$ and 0 for $h > 14$, then we have the ACC shown in the figure, with capability $\Psi = 9.86$ and generality $\mathit{\Gamma} = 0.39$.

A theoretical view also allows us to consider different options for difficulty. Having all tasks sliced by difficulty provides us with a way to understand the success of an individual in relation to the resources used. For instance, we can consider *difficulty as the complexity of the simplest solution*. In order to do this, we assume responses are binary or are binarised using a threshold, in successful or unsuccessful results, represented by A^{π}_{μ}, being 1 if π solves μ and 0 otherwise. There are few interesting consequences of this interpretation of difficulty. First, we have that for every agent, there is a difficulty from which its ACC is always zero, so the area is always finite[7]. Second, we can precisely determine how many solutions of a given difficulty there might be. For instance, we can calculate the resources according to different situations:

- We can consider difficulty as the length of the solution with lowest Kolmogorov complexity, i.e., $\hbar(\mu) \stackrel{\text{def}}{=} \min_{\pi : A^{\pi}_{\mu}=1} L(\pi)$ where $L(\pi)$ is the length of the solution π. Then the number of solutions for a given

[7]Basically that difficulty is determined by the maximum resources of the agent, because if it could be solved with fewer resources, the difficulty should be lower.

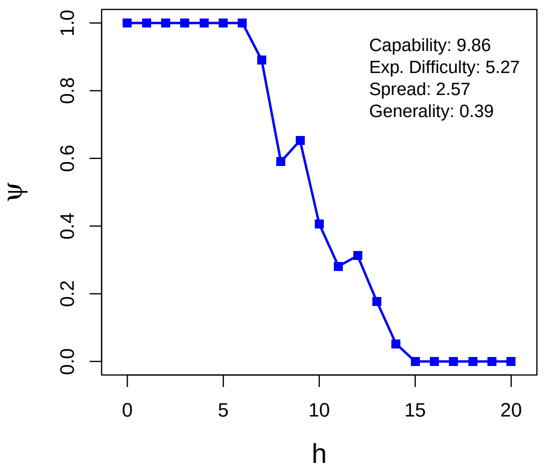

Figure S29: Average human results on exercises of different difficulty (h) in the C-test [9, 10], with the derived metrics shown on the plot.

difficulty h would be 2^h. In this situation, we can derive from the compactness property (#2) that the optimal curve is again one with $\Gamma = \infty$. To achieve capability Ψ, a non-learning system having predefined solutions for a large number of tasks would require a minimum of $\sum_{h=0}^{\Psi} h2^h = (\Psi - 1)2^{\Psi+1} + 2$ bits, plus the necessary code or neural wiring for making the switch among the $2^{\Psi+1} - 1$ solutions (assuming the solutions have nothing in common, because exhaustiveness here makes it difficult to compress this into a more hierarchical or reusable architecture). According to this situation, we can see that the "size" of the "brain" would grow more than exponentially. On the contrary, if instead of predefined solutions, we consider a learning system, the size would be reduced as much as we would like, but we would need to consider the availability of data and the learning effort instead.

- We can understand difficulty with Levin's Kt complexity, as advocated for in [9, 10, 104, 105, 91] because of its connection with Levin's optimal universal search [11, 12]. In this case, we define $LS(\pi, \mu) \stackrel{\text{def}}{=} L(\pi) + \log S(\pi, \mu)$ where L is the length of the solution π and S the computational steps μ uses to solve the task π. Difficulty would be $\hbar(\mu) \stackrel{\text{def}}{=} \min_{\pi: A_\mu^\pi = 1} LS(\pi, \mu)$. With this, we could still consider that the number of solutions for a given difficulty would be less than (but still approximately or linear with) 2^h. The result for a non-learning system would be then similar, but now we would have to take into account the time to determine which problem we are facing, which must choose between $2^{\Psi+1} - 1$ solutions. On the contrary, the resources needed for a learning system using this schema would just simply be the expected difficulty $\mathbb{H} = \Psi/2$. This is measured in the logarithm of computational steps[8], so the expected computational steps using a universal search would be $2^{\Psi/2}$.

From the above, we see the difference between a system with a predefined repertoire of solutions and a system that learns those solutions[9]. Even if the above ignores the training examples or interaction needed to learn the concepts, we see that there might be a trade-off between pre-wired and learned solutions, depending on the size limitations and the speed of the system.

The cases above are important to clarify the distinction between nature-vs-nurture and general-vs-specialised. Whereas we have the tendency to associate inherited functions with specialised functions, this does not have to be the case a priori, according to the definition of generality we are considering here. This may be a consequence depending on what resources are most relevant. Note that in the two analyses above, we derive the minimum resources following the compactness property (#2). Assuming all difficulties equally likely, one should focus on those policies that require fewer resources. Of course, if some particular pockets of problems of high difficulty are more likely than many problems of low difficulty then there is a rationale to cover those pockets specifically, so having less generality.

In all these cases we are using a distribution of tasks that is not based on a particular species or environment —they are not the tasks a human or animal would find in their lifetime. Accordingly, these distributions can be criticised as arbitrary. However, it is not true that all humans (and much less all animals) face the same fixed set of tasks. Precisely because of this, many psychometric tests include very abstract tasks, in an effort to be independent of particular human groups, and some (like Raven's matrices) may even look very unrelated

[8]These would be the *witts*, as used in property 8 in §E.2.

[9]Note that this is not related (and also looks apparently opposite) to the distinction between learning tasks and knowing tasks in [106].

to the natural (ancient or modern) environments humans face. However, it is well known that IQ tests lack measurement invariance when applied to other groups (e.g., people with some disabilities, children, etc.), non-human animals and, most especially, computers. In the latter case, it is not that they are particularly unfair for computers, but that AI systems can specialise for these tasks [107, 108, 109, 110]. In a way, we can get generality inside the test, but inability to extrapolate beyond the test. Restricting testing to a particular kind of tasks facilitates systems that specialise on them, and this is particularly exploited in AI.

Hence the relevance of diversity, and this idea of using all tasks with which we started. But, how much will the task distribution depend on the representational language or mechanisms used to derive the set of tasks? The invariance theorem, independently introduced by Solomonoff, Kolmogorov and Chaitin (see, e.g., [111]) says that any universal representational mechanism (language) can code any program as efficiently (in size) as any other up to a constant that is bounded by (but generally smaller than) the sizes of the definitions of both languages. This makes the concept of Kolmogorov complexity machine-independent, at least to an additive constant factor.

However, the definition of "universal intelligence" [99] has been criticised by this dependence on the reference machine, which is actually leading to different definitions according to what UTM is used to generate the universal distribution [102, 103, 91]. The main reason is that the invariance theorem appears in the exponent of the distribution ($2^{-K(x)}$), and the additive constant becomes an exponential one. In contrast, the two versions on the bottom of Fig. S28 put back the invariance theorem as an additive constant on the scale of difficulty. This means that the scale upon which all other measures are derived is relatively more stable. For instance, given the spread for an individual using a notion of difficulty on a reference machine, then this spread will be at most increased by a constant that does not depend on the individual. Also, as capability grows, the invariance theorem starts having more relevance. This can also be seen in the opposite way: systems with very limited resources (or capabilities) will be more dependent on the reference machine.

Still, using two different reference machines might lead to very different difficulties for the x-axis in the ACC and hence different capability and generality scores, which is of course what underlies many discussions about whether tests are biased against or in favour of a group. But there are many "bias equalisers", especially in testing, that can be used to determine capability and generality more independently [112, 113, 114]: 1) introducing a testing apparatus that is novel for all subjects, 2) analysing differences after ensuring that individuals are raised in or adapted to the same core knowledge, 3) present problems that have to be solved by combining or using a set of constructs or elements that are abstract and new. These procedures are common in animal cognition and human intelligence testing, but not that much in AI research [115, 116, 117].

In practice, we do not need to consider all possible tasks to derive metrics of generality in AI. We can do this for any test battery or benchmark for which we are interested in deriving the generality of a particular AI algorithm or agent, be it in machine learning, planning or machine translation. In order to start we only need a metric of difficulty. It does not have to be a universal metric, as described above, but a customised one instead. It can be derived in many ways:

- Anthropocentric difficulty: we can use human performance as a reference for the difficulty of a set of tasks. This can be obtained as an indicator that is inversely related to the success of average humans in each task.

- Populational difficulty: this can be derived by using a population of AI techniques for the range of problems. For instance, [118] apply IRT to derive the difficulty of machine learning instances. This idea can be applied to datasets and other kinds of problems in AI (e.g., the ALE benchmarks, [119]).

- Intrinsic difficulty: any meaningful characterisation of difficulty can be used here, as we have done in several cases in this paper. For instance, the difficulty of a planning problem can be based on a series of features about the problem:, such as the number of components, its structure, the degree of noise, etc. Note that difficulty is different from computational complexity, but time complexity may be an important factor.

- Integral difficulty: some other notions of difficulty can integrate space resources, computational time, energy consumption, data required, etc., [120] especially when including very different tasks. For instance, [121] aligns difficulty with the number of trees used by a random forest classifier, providing a very clean mapping to resources and effort.

- Opponent difficulty: in those cases where other agents compete or cooperate, we can use the capability of the opponents (or a measure inversely related to the capability of cooperators). Note that this makes this option populational as well.

Note that we are working with a notion of difficulty for an expected response above acceptability ($\mathbb{E}[r]_{j,i} \geqslant 1-\epsilon$), as defined in §E. So, for all of the above approaches to difficulty, one can vary the threshold ϵ, as a way of

generating variants of the same instance or task, from those with difficulty 0 ($\epsilon = 1$) to those with difficulty ∞ ($\epsilon = 0$, assuming no agent is 100% perfect).

The estimation of difficulty will usually have some associated error. If we assume this error to be constant independently of the magnitude of the difficulty, then we can have a similar result as for proposition 3, and then the theoretical ACC will be sigmoidal in shape (complementary to the error function). In the case of a view of difficulty in terms of simplest description, the error can be linked to the constants of the invariance theorem.

In general, whenever an evaluation procedure is established in AI, there is a selection of tasks from a certain domain and for a particular range of difficulties. For instance, one rarely finds Hofstadter's "Gödel, Escher, Bach" [122] as an instance for a machine translation benchmark. It is too hard to be discriminative for AI. Usually, the benchmark tasks are selected to cover an application area (usually of scientific or industrial interest) and the difficulty of the items is chosen such that they are neither too easy nor too difficult for the state-of-the-art algorithms. This is natural, but this is implicitly assuming a type of ACC nobody checks in the first place, and a very malleable notion of difficulty, adapted to the situation. This also makes the analysis of progress in AI hard to assess, as the tasks in the domain and their difficulty are changing, like a moving target.

D.3 Generality in competitions and benchmarks in AI

The options for difficulty seen above can be applied to an increasing range of AI competitions and benchmarks [123], especially those that are aiming at more general-purpose AI. Some of these are the general game playing AAAI Competition [124, 125], the reinforcement learning competition [126, 127] (which featured the 'polyathlon', with several domains), the genetic programming benchmarks [128, 129], the general video game competition [130, 21], and the arcade learning environment (ALE) [19, 130], a collection of Atari 2600 video games, which "has incentivized the AI community to build more generally competent agents" [131]. It is important to note that the introduction of new platforms and benchmarks where hundreds of tasks can be potentially be implemented [132, 133] is not usually accompanied with a verification of whether the agents that have highest performance are also more general (exceptions, [134, 135, 136]). Recognising that the diversity and difficulty of the tasks must be explicitly determined is one important outcome of our analysis so far, and one a metric of generality in these terms would help to flesh out.

The GVGAI competition [21, 23, 22], whose results we used in our experiments, aimed at general game playing. Even if the competition aims at *general* video game playing, hence the name, the focus is on finding non-transitivity, such that metalearning through hyper-heuristics and algorithm portfolios is effective [137, 138], by choosing different agents for different problems. This is also a common thing in ensemble methods [54], where diversity is positive if results are to be combined. The notion of transitivity is vindicated or assumed in indicators such as Elo rating [139], which depend on this transitivity, although more complex game topologies exist [140, 141, 142, 143].

We saw in Fig. 5 (top) in the chess scenario that difficulty is taken from the performance of the opponent. This is also especially interesting for systems that improve with self-play, like AlphaZero [80]. In these settings, it is important to check that the system does not get better and better against more competitive opponents but may end up losing (or drawing more frequently) against weak opponents. This leads us to the more general question of whether a system that develops over time becomes more or less general [144, 145]. As the system evolves, we may experience less flexibility but a wider covering of tasks, and this can be studied using metrics of capability and generality. We can see this in the series of systems from AlphaGo to MuZero. AlphaGo [146] was the first AI system programmed to defeat professional Go players at the ancient game of Go. The system relied on human data, domain knowledge and game rules to master the game. AlphaGo is a clear specialist (low generality), and the *actual* capability only appears for one task. Then, its successor, AlphaGo Zero [147], initially had no capability at Go, but it learnt from self-play only providing the game rules. Its potential capability for Go is thus very high (and very high actual capability after training), but it has very low actual and potential generality. AlphaZero [80] was then introduced with no actual capability on any game initially. But after self-playing, it excelled at a diversity of board games: Go, chess and shogi. AlphaZero has thus potential capability and some potential generality if we consider board games. Finally, we find MuZero [83], in the pursuit of more general-purpose algorithms. This system does not even require the rules of each game. MuZero combines AlphaZero's lookahead-search approaches with new planning abilities. MuZero is able to match the performance of AlphaZero in the above classic board games, but it also demonstrates significant success in environments without known dynamics such as the Atari 2600 video games. MuZero has similar or higher potential capability than AlphaZero but higher potential generality, as it can cover more games.

Another good example of increasing generality in AI is represented by large language models, which have achieved remarkable performance on a wide range of tasks [84, 148]. Their generality is actual rather than potential: they cover all these tasks without retraining, using zero-shot or few-shot inference. Of course, they still have many limitations and fail catastrophically at some very simple examples [149]. Nevertheless, there is great promise that with more parameters or jointly with other technologies in the future they could become general —yet not necessarily very capable— systems: *consistently good at a very wide range of very simple*

tasks.

In the end, the progress of some techniques in AI can be made in such a way that generality is preserved, and the ACCs are just *translated* to the right as the technology improves. We now have tools to check whether this is the case, or, on the contrary, some new techniques solve more challenging problems at the cost of being worse at simpler problems. This is particularly relevant as progress in AI can be attained by combining several approaches, in areas such as ensemble learning or portfolios, where a big switch approach determines which technique is most appropriate for a particular instance. This modular approach to solving problems may well end up in specialised solutions and creating gaps, where some relatively simple problems are not solved, with lack of generality. But this modular approach, combining many specialised solutions, if the set of tasks remains constant, may increase generality (and capability), especially if the combination covers more of the easy ones than the difficult ones. Again, we see that generality measures how capability is distributed in terms of difficulty, but it does not impose constraints on how this is done. It may even include human computation, collective systems, cognitive services or hybrids, in the same way that humans can be enhanced by personal assistants or other devices —and increase or decrease their generality because of this.

Of course, if a modular solution requires hundreds of specialised subsolutions, the cost of keeping all them and designing an appropriate and efficient switch to determine which one to use may end up being less efficient than a more integrated solution, as we have discussed above. The relation between generality and resources is another way of looking at compression and generalisation, Occam's razor, the MML principle, etc., in machine learning, genetic programming and other areas in AI (e.g., [150, 151, 152]). Actually, the issues of generalisation and difficulty were usual (although from a different perspective) in the early days of genetic programming [153] (using the term 'generality' as 'generalisation power' or 'avoiding overfitting'). Commonly, the notion of generalisation is usually linked to whether a model extrapolates from the training data to the test data [154], and a proper validation will just equate this with performance [155]. But, generality, as introduced in this paper, just measures the distribution of success across difficulties, and can be applied to learning problems, planning problems, deductive problems, more in the original spirit of McCarthy [76].

E Formal setting and properties: Further detail

We will consider the evaluation of a set of M agents on a set of N tasks, with results or responses $r_{j,i}$ for each agent π_j and task μ_i, as represented in the example matrix (numbers in black) in Fig. 1 (top). For each agent we have its response mean $\bar{r}_j \stackrel{\text{def}}{=} \text{Mean}_i[r_{j,i}]$, also referred to as *agent average performance*, and its response variance $\sigma_j^2 \stackrel{\text{def}}{=} \text{Var}_i[r_{j,i}]$, also referred to as *agent variance*[10]. From here, we could simply define one notion of regularity as the reciprocal (inverse) of the variance. This would give us $1/\sigma_a^2 = 1/0.016 = 64.0$ and $1/\sigma_b^2 = 1/0.203 = 4.92$ for agents a and b respectively in Fig. 1.

But is the variance produced by unreliability in the measurement, instability in the agent or is it because the agent really performs much better at some problems than others? We could try to exclude all sources of unreliability and work at the definitional level. In order to do this, instead of actual responses, we could work with expected (or ideal) responses. For each agent π_j and an instance or task μ_i, the expected response is given by $\mathbb{E}[r]_{j,i}$. We assume $0 \leqslant \mathbb{E}[r]_{j,i} \leqslant 1$ with 0 meaning worst possible performance and 1 meaning best possible performance. We would then discretise expected responses as $A_{j,i} = 1$ ('acceptable' or 'accomplished') if $\mathbb{E}[r]_{j,i} \geqslant 1-\epsilon$ and 0 otherwise ('unacceptable' or non-accomplished), where $1-\epsilon$ would be just a threshold[11]. For instance, for dichotomous tasks (where agents can only be right or wrong), with an $\epsilon = 0.3$, we could have that $A_{j,i}$ is 1 if the agent is expected to be correct on the instance at least 70% of the times.

This simple transformation would eliminate reliability issues in our analysis of generality. But still, could then we define generality as being good for all possible problems? First, for many sets of tasks N it is not possible to have acceptable results for all of them, as some may be very complex or may require more resources than the agent has. Second, by using binary acceptability, we would have a Bernoulli distribution, and the variance would just be derived from the agent's average performance ($\bar{r}_j \cdot (1-\bar{r}_j)$) thus making it impossible to compare the generality of agents with equivalent performance. Part of this connection can be translated to spread. Consider that all the items are in some interval of difficulties, and hence capabilities $0..q$. If we have N items and an agent is right on all of them, then the ACC would be completely packed on the left, and spread would be 0. If an agent is wrong on all of them, then the ACC would be zero from 0 to q, but assuming that the curve is full (saturated) on the left (translation property) then we would still have a spread of 0. Between these two extreme cases in capability (both with spread 0), as we sort responses by difficulties in the ACC, and we calculate spread in a different way from variance, we can have results that deviate significantly from the Bernoulli distribution. For more discussion about this and its relation to the metrics we are about to introduce, see Appendix F.5.

[10]For the calculation of Var we use the population variance, not the sample variance, so we divide by N, not $N-1$.

[11]The value of ϵ might be different for each task. Actually, by changing the threshold we change the difficulty of the task, which is actually like having another task.

The way-out of these two problems is to look at responses in terms of their *difficulty*. Actually, agents might be better for easy problems than for hard ones. The quantification of difficulty appears at the core of generality.

E.1 Agent characteristic curves (ACCs) and capability: detail

Let us then consider a difficulty function, $\hbar$, mapping each task μ_i to a real value $\hbar(\mu_i) \geqslant 0$. We define an agent characteristic *plot* for agent π_j as a scatter plot showing accomplishment $A_{j,i}$ in terms of the difficulty $\hbar_i$. In other words, we plot difficulty on the x-axis and accomplishment on the y-axis.

We can convert these scatter plots (as the dots are always 0s and 1s) into more interpretable curves. In order to do this, we define $\psi_j(h) \stackrel{\text{def}}{=} \mathbb{P}(A_{j,i} = 1 | \hbar(\mu_i) = h)$, or equivalently, the mean of the accomplishment of agent j on all problems of difficulty h. We then define an agent characteristic curve (ACC) as a plot of $\psi_j(h)$ as a function of h. Fig. S30 shows six scatter plots (grey circles, all either 0 or 1) and their corresponding ACC (blue line).

We can look at the leftmost part of the curve. We say that an agent characteristic curve is s-saturated if $\forall h \leqslant s \,:\, \psi_j(h) = 1$. We see that the two first ACCs are not even saturated for $s = 0$. On the rightmost part, we want agent characteristic curves to ensure that the area under these curves is finite. We will assume difficulty functions that meet this property[12].

We copy the definition of capability we gave in Eq. 1,

$$\Psi_j \stackrel{\text{def}}{=} \int_0^\infty \psi_j(h)\, dh \tag{7}$$

i.e., the sum of all the mean responses per difficulty, which is the area under the ACC (see Fig. S30 for the calculated capabilities). Note that in a discrete way, capability is a weighted sum of all tasks according to a prior uniform distribution (or weight) of difficulties. The area will of course change even with a monotonic transformation on difficulty, such as a change to a logarithmic scale. Some scales make more sense than others and give a more meaningful notion of capability, especially if the x-axis can be associated with an additive unit, as we will discuss later on. These weights can be derived if we know the posterior, how many tasks we have for each difficulty.

Definition of spread and generality: detail

As capability represents the "mass" (how much of accomplishment we have), but not a probability density function, we can normalise this moment (dividing by capability) and we can interpret this as the expected difficulty for agent j for the successful items (binned by difficulty):

$$\mathbb{H}_j \stackrel{\text{def}}{=} \mathbb{E}_{h \sim f_j}[h] = \frac{M_j}{\Psi_j} \tag{8}$$

where $f_j(x) = \psi_j(x)/\Psi_j$. In other words, $\mathbb{H}_j$ is the expected difficulty conditioned to accomplishment, i.e., average difficulty for all successful item responses.

Now, if we look at $\mathbb{H}_j$ as an expected difficulty, then, for a distribution that is fully compacted on the left (a single step function), this should be *half* of the capability. This difference can be multiplied by capability back again and finally square rooted, to make it independent of location and with a unit commensurate with difficulty, as we will see. The result is known as *spread* (already introduced in Eq. 4), and is given alternatively by:

$$S_j \stackrel{\text{def}}{=} \sqrt{(2\mathbb{H}_j - \Psi_j) \cdot \Psi_j} \tag{9}$$

E.2 Properties: detail

We now explore the properties to see their full details. We first need to introduce the notion of difficulty *translation*, defined as a constant shift of the x-axis ($h + k \leftarrow h$). If k is negative we have a translation to the left, where every result with $h < 0$ is cut out. If k is positive we have a translation to the right, and we assume that $\psi_j(h) = 1$ for all $h < k$ (i.e., we saturate the newly introduced part of the curve). Second, we introduce the notation for partial areas, i.e., $\Psi_j^{[h_1:h_2]} \stackrel{\text{def}}{=} \int_{h_1}^{h_2} \psi_j(h)\, dh$.

Now we can introduce eight important properties. The proofs are given in §F.

1. Translation: any positive translation by k implies that capability becomes $\Psi_j + k$. The same happens for negative translation if the $|k|$-leftmost part of the original curve was saturated. On the other hand, generality is invariant to translation (with the same conditions as above for negative translation).

[12] We mentioned this is the previous section, but setting a threshold on tasks ensures curves are finite when difficulty is defined in terms of minimal resources [91], especially in situations where there is a minimum percentage given by chance. Another simpler option is just to set a maximum difficulty.

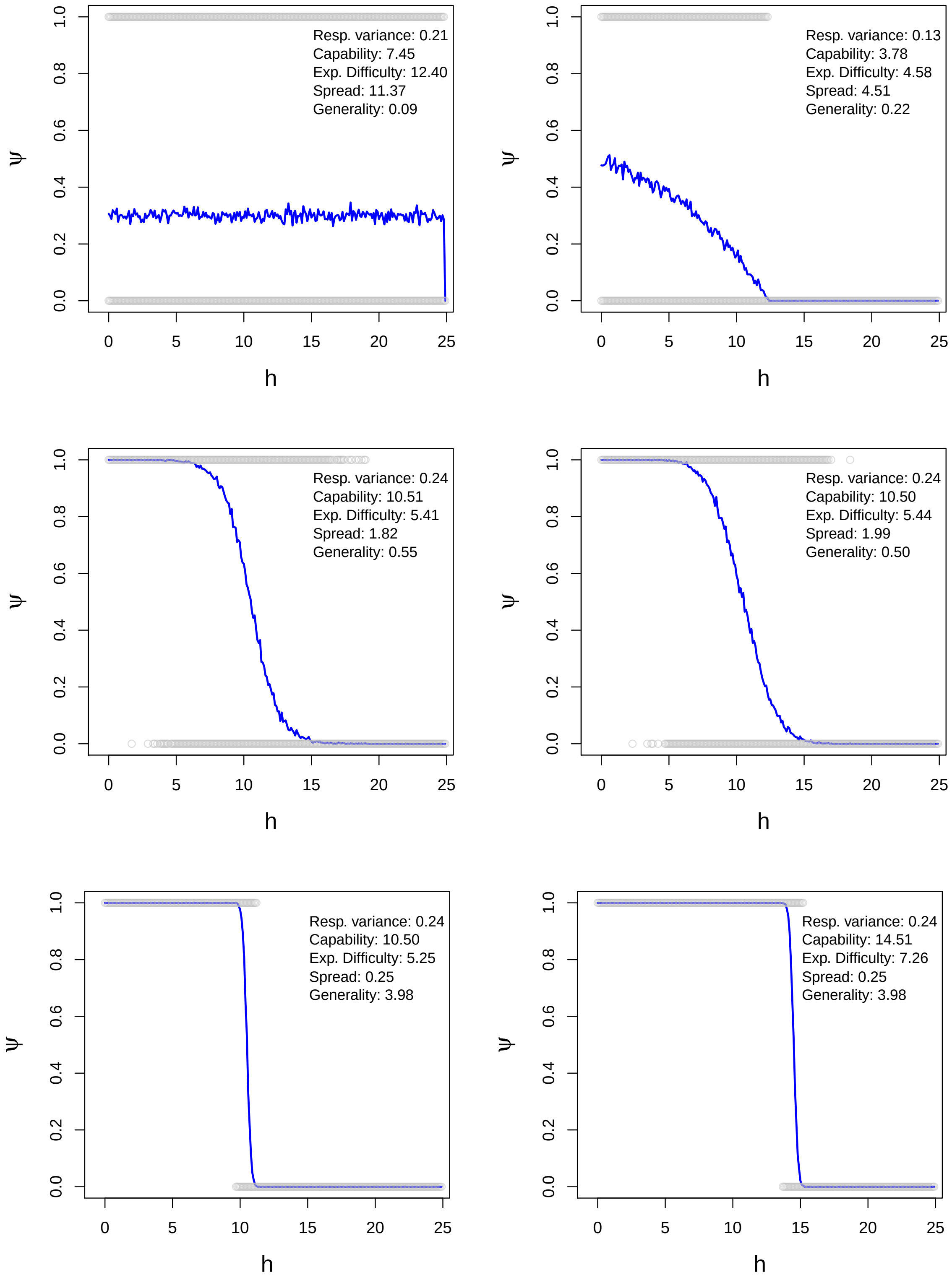

Figure S30: *Agent characteristic curves* (ACC), showing the behaviour of six different agents in terms of difficulty $\hbar$ on the x-axis. The responses $r_{j,i}$ for the items i are shown in grey circles. The means for each difficulty are shown in blue, and connected to form an ACC. We see that different distributions of results give different values for the metrics: response variance (σ_j^2), capability (Ψ_j), expected difficulty ($\mathbb{H}_j$), spread (S_j) and generality ($\mathit{\Gamma}_j$). Curves that have a steplike shape have high generality.

2. Compactness: with equal capability, any equal mass moved to the left of the plot such that $\Psi_j^{[h_1:h_2]} \leftarrow \Psi_j^{[h_1:h_2]} + q$ while $\Psi_j^{[h_3:h_4]} \leftarrow \Psi_j^{[h_3:h_4]} - q$, with $h_2 < h_3$, will increase $\mathit{\Gamma}_j$.

3. Maximum generality (minimum spread): given a fixed capability Ψ_j, the minimum expected difficulty $\mathbb{H}_j$ and the maximum generality $\mathit{\Gamma}_j$ are obtained with a decreasing step agent characteristic function abruptly falling from 1 to 0 on $h = \Psi_j$, where the capability is double the expected difficulty (i.e., $\Psi_j = 2\mathbb{H}_j$), spread $S_j = 0$ and generality $\mathit{\Gamma}_j = \infty$.

4. Constant interval: Given a constant function $\psi_j(h) = c$ from 0 to q, we have $\Psi_j = cq$, $S_j = q\sqrt{c(1-c)}$ and $\mathit{\Gamma}_j = 1/(q\sqrt{c(1-c)})$. In the particular case of $c = 0.5$ we have $S_j = q/2$ and $\mathit{\Gamma}_j = 2/q$. Any spread greater than the value for this constant curve is considered 'abstruse'.

5. Minimum generality (maximum spread): given a fixed capability Ψ_j, and assuming $\psi_j(h) = 0$ beyond difficulty q, then the maximum expected difficulty $\mathbb{H}_j$ and the minimum generality $\mathit{\Gamma}_j$ (the most abstruse result in a bounded interval) are obtained with a increasing step agent characteristic function going sharply from 0 to 1 on $h = q - \Psi_j$ (until q), leading to $S_j = \sqrt{2\Psi_j(q-\Psi_j)}$ and $\mathit{\Gamma}_j = 1/\sqrt{2\Psi_j(q-\Psi_j)}$. With the same capability, the square spread for this case is exactly twice the square spread for the constant case.

6. Task transitivity: if an agent π_j is s-saturated then for every task μ_b such that $A_b^j = 1$ in the saturated area then for all other tasks a of $\hbar(\mu_a) \leqslant \hbar(\mu_b)$ we have that $A_a^j = 1$. In other words, if this agent solves a task in the saturated area then it also solves any other easier task. Agents with maximum generality $\mathit{\Gamma}_j = \infty$ are s-saturated with $s = \Psi_j$, so if a task of a given difficulty is solved we know all other simpler tasks are solved too.

7. Agent transitivity: if two agents π_a and π_b have maximum generality $\mathit{\Gamma}_a = \mathit{\Gamma}_b = \infty$ and $\Psi_a \leqslant \Psi_b$ then for every task μ_i such that $A_i^a = 1$ we have that $A_i^b = 1$. That means that π_b *dominates* π_a or, in other words, that an agent would solve all tasks a less capable agent solves, provided both have maximum generality. Note that if generality is not infinite, it is not sufficient to have a curve for π_b that covers the curve for π_a. We need to check that π_b is s-saturated for at least the maximum value where π_b gets non-zero accomplishment.

8. Same units: if we introduce a unit for difficulty, let us call it *witts*, then capability is also (additively) measured in *witts*, spread is also measured in *witts* and hence generality is measured in 1/*witts*.

Some of these properties (especially the transitivities) have been shown when assuming a Guttman (or deterministic) response model [35, 36], as we discuss in §B. Looking again at Fig. S30 we see that the bottom left and bottom right are basically a translation of each other by $k = 4$. We see that the capability is increased by approximately 4, and the spread and generality are not significantly affected.

F Theoretical results

In this appendix we include the proofs of the properties, their associated lemmata and propositions, the connection between spread and variance for a normal distribution, the aggregation of step curves, the derivation of Guttman curves through thresholding, and the derivation of the isometrics.

F.1 Proofs of the properties

Despite being straightforward, for completeness we include all the proofs of the properties presented in §E.2.

Proposition 4. *Given an agent with capability Ψ_j, any positive translation by k implies that capability becomes $\Psi_j + k$.*

Proof. A translation creates a new function such that $h' \leftarrow h - k$ and $\psi_j(h') = 1$ for all $h' < k$, so the new capability Ψ'_j is now:

$$\Psi'_j = \int_0^k 1\, dh' + \int_k^\infty \psi_j(h-k)\, dh = k + \Psi_j$$

□

Proposition 5. *Given an agent with capability Ψ_j where the $|l|$-leftmost part of the original curve was saturated, any negative translation by $k \leqslant l$ implies that capability becomes $\Psi_j - k$.*

Proof. As the left part of the curve is saturated, Ψ_j can be decomposed into

$$\Psi_j = \int_0^l 1\, dh + \int_l^\infty \psi_j(h)\, dh$$

Now the translation removes part of the first term, so the new capability is:

$$\Psi'_j = \int_0^{l-k} 1\, dh + \int_{l-k}^{\infty} \psi_j(h+l-k])\, dh = l-k+\Psi_j - l = \Psi_j - k$$

□

Proposition 6. *With the same conditions as the above two propositions, generality is invariant to translation.*

Proof. For a positive translation, we have that the new effort M'_j equals:

$$\begin{aligned} M'_j &= \int_0^k h' \cdot 1\, dh' + \int_k^{\infty} h\psi_j(h-k)\, dh \\ &= \frac{k^2}{2} + \int_k^{\infty} (h-k)\psi_j(h-k)\, dh + \int_k^{\infty} k\psi_j(h-k)\, dh \\ &= \frac{k^2}{2} + M_j + k\Psi_j \end{aligned}$$

From proposition 4 we have that $\Psi'_j = k + \Psi_j$. Putting both things together into the definition of spread (Eq. 9), we have:

$$\begin{aligned} S'_j &= \sqrt{2M'_j - \Psi'^2_j} = \sqrt{2\frac{k^2}{2} + 2M_j + 2k\Psi_j - (k+\Psi_j)^2} \\ &= \sqrt{k^2 + 2M_j + 2k\Psi_j - k^2 - 2k\Psi_j - \Psi_j^2} = \sqrt{2M_j - \Psi_j^2} \end{aligned}$$

As generality is the reciprocal of spread, and spread does not change, then it is invariant to positive translation. The proof for the negative translation is similar. □

Proposition 7. *Compactness: any mass moved to the left of the plot such that $\Psi_j^{[h_1:h_2]} \leftarrow \Psi_j^{[h_1:h_2]} + q$ while $\Psi_j^{[h_3:h_4]} \leftarrow \Psi_j^{[h_3:h_4]} - q$, with $h_2 < h_3$ will increase Γ_j.*

Proof. Clearly, $\Psi'_j = \Psi_j$, since the same mass q is included in the integral one way or the other. We have that the new effort M'_j:

$$M'_j = \int_0^{h_1} h\psi_j(h)\, dh + \int_{h_1}^{h_2} h\psi'_j(h)\, dh + \int_{h_2}^{h_3} h\psi_j(h)\, dh + \int_{h_3}^{h_4} h\psi'_j(h)\, dh + \int_{h_4}^{\infty} h\psi_j(h)\, dh$$

Since $h_2 < h_3$, we have that $\int_{h_1}^{h_2} h\psi'_j(h)\, dh + \int_{h_3}^{h_4} h\psi'_j(h)\, dh < \int_{h_1}^{h_2} h\psi_j(h)\, dh + \int_{h_3}^{h_4} h\psi_j(h)\, dh$, and hence $M'_j < M_j$. Now, from the definition of spread (Eq. 9), we have:

$$S'_j = \sqrt{2M'_j - \Psi'^2_j} = \sqrt{2M'_j - \Psi_j^2} < \sqrt{2M_j - \Psi_j^2} = S_j$$

As spread is smaller, and generality is the reciprocal, this completes the proof. □

Corollary 8. *Maximum: given a fixed capability Ψ_j, the minimum expected difficulty $\mathbb{H}_j$ and the maximum generality Γ_j are obtained with a step agent characteristic function on $h = \Psi_j$.*

Proof. By proposition 7, generality is increased as far as we move mass of the function from right to left, while keeping the area constant. This means that the maximum area with highest generality is obtained by a step function, whose location must be on $h = \Psi_j$. □

Proposition 9. *Decreasing step function (maximum generality, minimum spread): given a step function, capability is double the expected difficulty (i.e., $\Psi_j = 2\mathbb{H}_j$), and generality $\Gamma_j = \infty$.*

Proof. The area of a step function with location l is:

$$\Psi_j = \int_0^l 1\, dh = l$$

As effort in this situation is:

$$M_j = \int_0^l h \cdot 1\, dh = \frac{l^2}{2}$$

Expected difficulty is just:

$$\mathbb{H}_j = \frac{M_j}{\Psi_j} = \frac{l^2}{2} = \frac{l}{2}$$

So $\Psi_j = 2\mathbb{H}_j$ and $S_j = \sqrt{2\frac{l^2}{2} - l^2} = 0$, so its reciprocal is ∞. □

Proposition 10. *Constant curve in an interval: given a constant function $\psi_j(h) = c$ from 0 to q, we have $\Psi_j = cq$, $S_j = q\sqrt{c(1-c)}$ and $\Gamma_j = 1/(q\sqrt{c(1-c)})$.*

Proof. We have:

$$\Psi_j = \int_0^q c\,dh = cq$$

and

$$M_j = \int_0^q h \cdot c\,dh = c\frac{q^2}{2}$$

and

$$S_j = \sqrt{2M_j - \Psi_j^2} = \sqrt{2c\frac{q^2}{2} - (cq)^2} = \sqrt{c(1-c)q^2} = q\sqrt{c(1-c)}$$

□

We have two examples at the bottom of Fig. S22. For instance, on the left we have $\Psi_j = cq = 0.2 \cdot 2 = 5 \approx 4.97$, $S_j = \sqrt{c(1-c)}q = \sqrt{0.2(0.8)}25 = 10 \approx 9.93$ and $\Gamma_j = 1/\sqrt{c(1-c)}q = 1/10 \approx 0.10$. The precision divergence is given because the curves are not perfectly flat.

Proposition 11. *Minimum generality (maximum spread): given a fixed capability Ψ_j, and assuming $\psi_j(h) = 0$ beyond difficulty q, then the maximum expected difficulty $\mathbb{H}_j$ and the minimum generality Γ_j (the most abstruse result in a bounded interval) are obtained with an increasing step agent characteristic function on $h = q - \Psi_j$ (until q), leading to $S_j = \sqrt{2\Psi_j(q-\Psi_j)}$ and $\Gamma_j = 1/\sqrt{2\Psi_j(q-\Psi_j)}$.*

Proof. We have:

$$M_j = \int_0^q h \cdot \psi_j(h)\,dh = \int_{q-\Psi_j}^q h \cdot 1\,dh = \left[\frac{h^2}{2}\right]_{q-\Psi_j}^q = \frac{1}{2}(q^2 - (q-\Psi_j)^2)$$

and

$$S_j = \sqrt{2M_j - \Psi_j^2} = \sqrt{(q^2 - (q-\Psi_j)^2) - \Psi_j^2} = \sqrt{2\Psi_j(q-\Psi_j)}$$

□

Proposition 12. *With the same capability, the square spread for the minimum case (most abstruse case in an interval) is exactly twice the square spread for the constant case (in the same interval).*

Proof. For the constant case we had that $\Psi_j = cq$, plugging this into the result of S_j for the constant case, we have:

$$S_j = q\sqrt{c(1-c)} = \sqrt{q^2\frac{\Psi_j}{q}\left(1 - \frac{\Psi_j}{q}\right)} = \sqrt{\Psi_j(q-\Psi_j)}$$

Comparing with S_j for the minimum case, and squaring them, we see that one doubles the other. So we have shown that the square spread for this minimum case is exactly twice the square spread for the constant case. □

Proposition 13. *Task transitivity: if an agent π_j is s-saturated then for every task μ_b such that $A_b^j = 1$ in the saturated area then for all other tasks a with $\hbar(\mu_a) \leqslant \hbar(\mu_b)$ we have that $A_a^j = 1$.*

Proof. If an agent s is s-saturated then $A_b^j = 1$ for all tasks such that $\hbar(\mu_b) \leqslant s$. If b is in the saturated area, any other task a of lower difficulty also is. □

Proposition 14. *Agent transitivity: if two agents π_a and π_b have maximum generality $\Gamma_a = \Gamma_b = \infty$ and $\Psi_a \leqslant \Psi_b$ then for every task μ_i such that $A_i^a = 1$ then $A_i^b = 1$.*

Proof. It is sufficient to see that both agents will have step functions. □

Note that if the generality of π_b is not infinite, it is not sufficient to have a curve for π_b that covers the curve for π_a. The reason is that there might be values of h for which $0 < \psi(h)_a < \psi(h)_b < 1$, and in these cases some of tasks that make the non-zero value in $\psi(h)_a$ might not be in the tasks that make the value of $\psi(h)_b$.

Proposition 15. *Same units: if we introduce a unit for difficulty, let us call it witts, then capability is also measured in witts, spread is also measured in witts and hence generality is measured in 1/witts.*

Proof. As Ψ_j is an integral over difficulty and the domain of the function is unitless (accomplishment, which is a proportion), then Ψ_j has the same units as difficulty. As M_j includes the factor h in the integral, i.e.,

$$M_j = \int_k^\infty h\psi_j(h)\,dh$$

the result is in $witts^2$. Finally, from the definition of spread:

$$S_j = \sqrt{2M_j - \Psi_j^2}$$

we get $\sqrt{witts^2}$, which means that spread is measured in *witts*, and the reciprocal for generality. □

F.2 Proofs when using a normal distribution for capability

Here we include the proofs about the case where the ACC derives from assuming a normal distribution on capability. This can be interpreted as an aggregation of step curves where the capability of each is distributed normally or an agent such that the probability of a correct response depends on the CDF of a normal distribution using the difference between the capability and the instance difficulty.

Proposition 16. *(proposition 1 in the paper) Assuming a normal distribution on capability, with standard deviation σ, the slope of the ACC will be $-\frac{1}{\sigma\sqrt{2\pi}}$.*

Proof. (of proposition 1) We know that a normal distribution with mean μ and standard deviation σ will lead to the following agent characteristic curve:

$$\psi_j(h) = 1 - \Phi\left(\frac{h-\mu}{\sigma}\right)$$

with Φ being the CDF of the standard normal distribution and ϕ being the density function of the standard normal distribution. The maximum slope of this is the first derivative at μ, which is:

$$\text{slope} = -\phi\left(\frac{\mu-\mu}{\sigma}\right) = -\frac{1}{\sqrt{2\pi\sigma^2}}e^{-\frac{(0)^2}{2\sigma^2}} = -\frac{1}{\sqrt{2\pi\sigma^2}}$$

and hence the slope of the ACC will be $-\frac{1}{\sigma\sqrt{2\pi}}$. □

Lemma 17. *(lemma 2 in the paper) Assuming a normal distribution on the capability, with mean μ and standard deviation σ, such that the location is sufficiently beyond 0 to have negligible mass below 0 (i.e., $\frac{\mu}{\sigma} \gg 0$), we have that $M_j = \frac{\sigma^2+\mu^2}{2}$.*

Proof. (of lemma 2) As in proposition 1, we know that a normal distribution with mean μ and standard deviation σ will lead to the following agent characteristic curve:

$$\psi_j(h) = 1 - \Phi\left(\frac{h-\mu}{\sigma}\right)$$

We plug this into the definition of effort and operate a little bit on it in order to put the expression in terms of the cumulative distribution function Φ of the normal distribution:

$$\begin{aligned} M_j &= \int_0^\infty h \cdot \left(1 - \Phi\left(\frac{h-\mu}{\sigma}\right)\right)\,dh \\ &= -\int_{-\infty}^0 h \cdot \Phi\left(\frac{h+\mu}{\sigma}\right)\,dh \end{aligned}$$

Fortunately, we can find the following integral of the moment of the CDF on page 402 (second last, entry 10,001) in [156]:

$$\int x\Phi(a+bx)\,dx = \frac{1}{2b^2}\left((b^2x^2-a^2-1)\Phi(a+bx)+(bx-a)\phi(a+bx)\right)+C$$

And ϕ is the density function.

In our case, $a=\frac{\mu}{\sigma}$ and $b=\frac{1}{\sigma}$, so we can put all things together into:

$$\begin{aligned} M_j &= -\left[\frac{1}{2b^2}\left((b^2x^2-a^2-1)\Phi(a+bx)+(bx-a)\phi(a+bx)\right)\right]_{-\infty}^{0} \\ &= -\left[\frac{1}{2b^2}\left((-a^2-1)\Phi(a)-a\phi(a)\right)\right]-[0+0]] \\ &= \frac{1}{2\left(\frac{1}{\sigma}\right)^2}\left(\left(\left(\frac{\mu}{\sigma}\right)^2+1\right)\Phi\left(\frac{\mu}{\sigma}\right)+\frac{\mu}{\sigma}\phi\left(\frac{\mu}{\sigma}\right)\right) \end{aligned}$$

Since we are assuming that $\frac{\mu}{\sigma}\gg 0$, we have that $\Phi(\frac{\mu}{\sigma})\approx 1$ and $\phi(\frac{\mu}{\sigma})\approx 0$, so we get:

$$\begin{aligned} M_j &= \frac{1}{2(\frac{1}{\sigma})^2}\left(\left(\frac{\mu}{\sigma}\right)^2+1\right)=\frac{\sigma^2\frac{\mu^2}{\sigma^2}+\sigma^2}{2} \\ &= \frac{\mu^2+\sigma^2}{2} \end{aligned}$$

□

Proposition 18. *(proposition 3 in §B.1) With the same assumptions as lemma 2, we have that spread $S_j=\sigma$ and $\Gamma=\frac{1}{\sigma}$.*

Proof. (of proposition 3) As the normal distribution is symmetric, we have that the location of the CDF is of course μ, so the capability $\Psi_j=\mu$, and plugging M_j from lemma 2, we have:

$$\begin{aligned} S_j &= \sqrt{2M_j-\Psi_j^2}=\sqrt{2M_j-\mu^2} \\ &= \sqrt{2\left(\frac{\mu^2+\sigma^2}{2}\right)-\mu^2}=\sigma \end{aligned}$$

And by the definition of generality we have $\Gamma_j=\frac{1}{\sigma}$. □

F.3 Aggregation

We show the following result:

Lemma 19. *Given N step ACCs with capabilities $a_1,a_2,\ldots,a_N$, if we average them into a single ACC, we have a descending staircase ACC with average capability $\Psi_j=\frac{1}{N}\sum_{i=1}^{N}a_i$ and spread S_j being the standard deviation of the original capabilities.*

Proof. We first consider the straightforward claim that $\Psi_j=\frac{1}{N}\sum_{i=1}^{N}a_i$. As each component has capability a_i, their average will make the capability of the composite, as areas are additive.

Now, let us work with the definition of moment. And let us choose an index such that the capabilities $a_1,a_2,\ldots,a_N$ of the N components are sorted by increasing capability, and for ease of notation, let us consider a value $a_0=0$. This means that from difficulties h from $a_0=0$ to a_1 we will have that all the N components are correct, so that the response $\psi_j(h)$ is $\frac{N}{N}=1$, from a_1 to a_2 we will have that $N-1$ components will be correct, so that the response is $\frac{N-1}{N}$, and so on, until the segment beyond a_N, whose response is zero. So,

$$
\begin{aligned}
M_j &\stackrel{\text{def}}{=} \int_0^{\infty} h \cdot \psi_j(h)\, dh \\
&= \int_0^{a_1} h \cdot \frac{N}{N}\, dh + \int_{a_1}^{a_2} h \cdot \frac{N-1}{N}\, dh + \cdots + \int_{a_{N-1}}^{a_N} h \cdot \frac{1}{N}\, dh \\
&= \sum_{i=1}^{N} \frac{N-i+1}{N} \int_{a_{i-1}}^{a_i} h\, dh \\
&= \sum_{i=1}^{N} \frac{N-i+1}{N} \left[\frac{h^2}{2}\right]_{a_{i-1}}^{a_i} \\
&= \sum_{i=1}^{N} \frac{N-i+1}{N} \left(\frac{a_i^2 - a_{i-1}^2}{2}\right) \\
&= \frac{1}{2}\left(\sum_{i=1}^{N} \frac{N-i+1}{N} a_i^2 - \sum_{i=1}^{N} \frac{N-i+1}{N} a_{i-1}^2\right) \\
&= \frac{1}{2}\left(\frac{1}{N} a_N^2 + \sum_{i=1}^{N-1} \frac{N-i+1}{N} a_i^2 - \sum_{i=1}^{N-1} \frac{N-i}{N} a_i^2 - \frac{1}{N} a_0^2\right) \\
&= \frac{1}{2}\left(\frac{1}{N} a_N^2 + \sum_{i=1}^{N-1} \frac{N-i+1-(N-i)}{N} a_i^2\right) \\
&= \frac{1}{2}\left(\frac{1}{N} a_N^2 + \frac{1}{N} \sum_{i=1}^{N-1} a_i^2\right) \\
&= \frac{1}{2N} \sum_{i=1}^{N} a_i^2
\end{aligned}
$$

Now, we plug everything into the definition of spread

$$
\begin{aligned}
S_j &\stackrel{\text{def}}{=} \sqrt{2M_j - \Psi_j^2} \\
&= \sqrt{2\frac{1}{2N} \sum_{i=1}^{N} a_i^2 - \left(\frac{1}{N} \sum_{i=1}^{N} a_i\right)^2} \\
&= \sqrt{\frac{1}{N} \sum_{i=1}^{N} a_i^2 - \left(\frac{1}{N} \sum_{i=1}^{N} a_i\right)^2}
\end{aligned}
$$

which is the expression of population standard deviation for the values $a_1, a_2, \ldots, a_N$. □

F.4 Scale transformations: from incommensurate values to thresholding

For many existing and future results in comparative cognition, psychometrics and AI we start with a matrix of results $r_{j,i}$ for task i and agent j. Each response can be a numeric score, or an aggregation of several items, as the values in black in Fig. 1. In very controlled scenarios, where the magnitudes of each task are binary[13] or correspond to a percentage, or at least in the same scale, we can extract some insight by looking at the variance of rows [157], —but remember the issues of the variance of a Bernoulli distribution as seen in §E, which we will discuss further in the following subsection. In general, however, looking at the variance for rows could be very misleading, as the original magnitudes can increase or decrease this variance in a spurious way. For instance, what if task μ_3 had values in a close interval between 0 and 1, and μ_7 ranges in a much larger interval? With this problematic proxy for generality task μ_7 would simply have more weight. If no further information about the tasks is available, it is hard to put the several columns of this matrix in a way that they are commensurate[14].

[13] Remember the issues of the variance of a Bernoulli distribution as seen in §E, which we will discuss further in the following subsection.

[14] The authors of [135], in https://deepmind.com/blog/article/Agent57-Outperforming-the-human-Atari-benchmark, illustrate this point near their Figure 1, when they realise that averaging rough scores for different games has no meaning, as the scales are different. However, they further state that "this issue is exacerbated if some tasks are much easier than others. By performing very well on very easy tasks, agent A can apparently outperform agent B, which performs well on both easy and hard tasks". This is an interpretation of generality that looks quite opposite to ours, but note that if we have an agent that solves many instances

The commensurability problem is related to the issue of deriving difficulties when no intrinsic difficulty is available. When the magnitudes are not commensurate and difficulty is not given, we need a point of reference. One possibility in these cases is to use a reference agent. For instance, in AI, it is quite common to use humans as a reference, and convert the score for a game or a task into a binary value that simply represents whether the agent is above human level. This is the usual approach in collections such as the Atari Games in ALE [19, 158]. In fact, for this scenario, we have used the ARef transformation in Table 1, which simply converts the score to binary values representing whether the AI system reaches human performance or not. But then we calculate generality as we have introduced in this paper, using task difficulty, and not as the non-informative variance of the Bernoulli distribution that appears as result of the binarisation.

Another common option to make the row variance more meaningful is to normalise the columns (with same column mean and variance) or to use ranks, which in both cases implies a transformation that depends on the particular population. More precisely, the normalisation of $r_{j,i}$ consists of subtracting the mean and dividing by the standard deviation. The conversion of $r_{j,i}$ into ranks would consist of replacing each value in a column i of the matrix by the rank in that column, with values in $1, 2, \ldots, M$, with M being the highest rank and 1 is the lowest rank. In either case, we can now calculate a variance or standard deviation per row that can be more meaningful, as was attempted in the top matrix of Fig. 1.

If this variance is more meaningful now, can we compare it with generality? In order to do this, we can convert each new value into an actual (step) ACC using the value as a threshold. Namely, we convert each response in the response matrix into a step ACC with capability at the $r_{j,i}$, or in other words, we assume that $\psi_j(h) = 1$ iff $r_{j,i} \geqslant h$, and 0 otherwise. With this trick, we use the population results as difficulty and we convert each test into a series of instances of different difficulty. With this configuration, we can now calculate the generality of each row by simply averaging the step ACCs, following the results of lemma 19, and we get a generality that is simply the reciprocal of the row standard deviation using the normalised or ranked values. This approach corresponds to the Rnk method in Table 1 when the $r_{j,i}$ are converted into rank values.

Indeed, this rank normalisation is done in [24] (as described in Table S2 and then used in Table S5 of their supplementary material). This can be seen as a way of circumventing the problem of not having a commensurate difficulty for all tasks. Under this rank transformation, we can use the rank as a metric of difficulty that is commensurate for all tasks (for this population of 53 orangutans). With this particular configuration, we could apply the thresholding approach above and connect row variance with generality.

Of course, this thresholding method assumes a step model, which is too simplistic. As an alternative, we could use IRT instead. For instance, [159] normalise the data and then use IRT models to work with a *different* metric of generality, based on the variance and estimated with a proxy: the slope of the curves. This is related to, but significantly different from, applying IRT and then using GA as suggested by the IRT transformation in Table 1.

The minimum sizes in Table 1 (approximate rules of thumb in some cases or statistical analysis in others) are derived from [160, 161]. Finally, the individual generality score can be applied to situations where the difficulty of a task depends on other agents taking place in a competing or cooperating role, using the Opp transformation in Table 1, which is especially necessary for social and adversarial situations [162, 163, 164]

F.5 Bernoulli limits and Interval Relative Squared Spread (IRSS)

In §E, we saw that considering the row variance as some kind of (inverse) proxy for generality did not work: if we were using a binary acceptability, we would have a Bernoulli distribution, and the variance would be determined by an average of results $(\bar{r}_j \cdot (1 - \bar{r}_j))$. Variance would be a quadratic function of r_j, with minima (0) for minimum and maximum average responses (0 and 1 respectively) and maxima (0.25) at 0.5, as we saw in Fig. S4.

From propositions 9, 11, 10, we identified the highest generality (lowest spread), lowest generality (highest spread) and the generality and spread for a constant curve of height c in this interval. In Fig. 4 (top left). we show an example of 496 results going from a minimum capability of 8.88 to a maximum capability of 19.41, as shown in the x-axis. The figure shows the curves for the maximum spread (in red), a constant curve spread (in blue) and the minimum spread (in green). We see how the points are all located under the red curve and most of them (except for 13.31%) below the blue curve.

Note that the interval does not start in 0, but the calculations can be adapted by subtracting the minimum value (in the example, 8.88) to the capabilities because of the translation property. Then, in Proposition 12, we showed that the squared spread for the minimum case (most abstruse case in an interval), $S^2_{max} = 2\Psi(q - \Psi)$, is exactly twice the squared spread for the constant case (in the same interval): $S^2_{cnst} = \Psi_j(q - \Psi_j)$, and at the same distance then to the minimum: $S^2_{min} = 0$.

---

of low difficulty, this would change average performance but not capability. It is the proportion of each difficulty what matters. Nevertheless, they do not introduce task difficulty in the analysis, and end up using several percentiles [135, Table 1], suggesting a column-wise correlational analysis instead, even if this requires some other assumptions and is performed at the level of the population.

That means that the expression in the Bernoulli constraint is now translated into squared spreads in a band that goes from 0 to twice the value $\Psi_j(q - \Psi_j)$. And a constant ACC (once for which correct or incorrect responses do not depend at all on difficulty) would be at the middle. This suggests that we could use a normalisation of spread, as was given in Eq. 6, copied here for convenience:

$$s^2 \stackrel{\text{def}}{=} \frac{S^2 - S^2_{cnst}}{S^2_{max} - S^2_{cnst}}$$

This Interval Relative Squared Spread (IRSS) —or simply *normalised spread*— goes from −1 (minimum spread, maximum generality) to 1 (maximum spread, minimum generality). Because of this reverse relation of spread to generality, for convenience, we use the term *normalised generality* as the negative value of normalised spread, i.e., $\gamma \stackrel{\text{def}}{=} -s$, also going from −1 (minimum generality) and +1 (maximum generality). Normalised generality does not have units, and we can use it to compare situations with different difficulty scales.

Note that normalised generality depends on the width if the interval (q). For the same agent, with a wider range of difficulties, the normalised generality would be more positive, while the unnormalised generality (and spread) would not change. While the range $(-1, 1)$ might resemble a correlation between difficulty and accomplishment, the right interpretation of the normalised generality must be as follows: for the items inside a range of difficulties, a high number means that the system is good at those with low difficulty and bad at those with high difficulty. Again, values of γ closer to the maximum value (1) would mean that there's a step curve, also meaning that difficulty is very predictable about the behaviour of the system.

G Reproducibility: Code and Data

Code and data for reproducibility is available at `https://github.com/jorallo/generality`, under GNU General Public License (GPL). We use the R programming language [165], which allows us, and everyone else, to easily compare results with a number of packages for factor analysis, item response theory, etc. Unlike factor analysis and item response theory, the code for generality analysis does not use any random component, or any estimation algorithm, and does not rely on parameters (other than the kind of study or options for representation), so the metrics are completely deterministic and perfectly reproducible. Running the code again should lead to the same numerical results and representations.

The library includes many functions for analysis and representations. Some of them plot individual ACCs or perform the full generality analysis, with optional comparison with factor analysis, difficulty extracted from IRT, etc. All the transformations in Table 1 are also available. The functions are implemented in `generality_functions_v.X.X.X.R` with a wrapper in `generality.R` that is independent of the version. We suggest to start with `demo_v.X.X.R`, which includes a simple example on how the functions work.

The library includes particular R files for the following scenarios:

- `ale`: the Atari video games Arcade Learning Environment (§A.7).
- `chess`: the World Computer Chess Championship (§A.6).
- `ctest`: Thurstone letter series using results using the C-test instances (main paper and §A.2).
- `damerius`: Physical cognition tasks (§A.9).
- `dicarlo`: Object recognition problems (main paper and §A.3).
- `gvgai`: General video game AI (§A.8).
- `herrmann`: The Primate Cognition Test Battery (§A.10).
- `lambda`: Lambda-One, a benchmark used to compare humans and simple reinforcement learning algorithms, as used in Fig. 2 in the main paper.
- `mazes`: Elithorn's Perceptual Mazes (main paper and §A.1).
- `odorspan`: Odour Span Task (main paper and §A.4).
- `openml`: Iris classification problem from OpenML (§A.5).

There's also an `evolution.R` file that runs the experiments and plots in the supplementary material dealing with evolutionary selective pressure (section C.3).

The repository includes data folders for many of the above studies, and the data to run the generality analysis for each scenario.

References

[1] Elithorn, A. A preliminary report on a perceptual maze test sensitive to brain damage. *Journal of neurology, neurosurgery, and psychiatry* **18**, 287 (1955).

[2] Davies, A. D. & Davies, M. The difficulty and graded scoring of Elithorn's perceptual maze test. *British Journal of Psychology* **56**, 295–302 (1965).

[3] Elithorn, A., Jones, D., Kerr, M. & Lee, D. The effects of the variation of two physical parameters on empirical difficulty in a perceptual maze test. *British Journal of Psychology* **55**, 31–37 (1964).

[4] Buckingham, R., Elithorn, A., Lee, D. & Nixon, W. A mathematical model of a perceptual maze test. *Nature* **199**, 676 (1963).

[5] Loe, B. S. & Sanchez, M. Maze generator. r package version 0.1.3. *https://cran.r-project.org/web/packages/mazeGen/mazeGen.pdf* **1** (2017).

[6] Thurstone, L. L. Primary mental abilities. *Psychometric monographs* (1938).

[7] Thurstone, L. & Thurstone, T. *Factorial studies of intelligence.* Psychometrika monograph suplements (The University of Chicago press, 1941).

[8] Simon, H. A. & Kotovsky, K. Human acquisition of concepts for sequential patterns. *Psychological Review* **70**, 534 (1963).

[9] Hernández-Orallo, J. & Minaya-Collado, N. A formal definition of intelligence based on an intensional variant of algorithmic complexity. In *Proceedings of International Symposium of Engineering of Intelligent Systems (EIS'98)*, 146–163 (1998).

[10] Hernández-Orallo, J. Beyond the Turing Test. *J. Logic, Language & Information* **9**, 447–466 (2000).

[11] Levin, L. A. Universal sequential search problems. *Problems of Information Transmission* **9**, 265–266 (1973).

[12] Levin, L. A. Universal heuristics: How do humans solve "unsolvable" problems? In Dowe, D. L. (ed.) *Algorithmic Probability and Friends. Bayesian Prediction and Artificial Intelligence*, vol. 7070 of *Lecture Notes in Computer Science*, 53–54 (Springer, 2013).

[13] Katayama, S. An analytical inductive functional programming system that avoids unintended programs. In *Proceedings of the ACM SIGPLAN 2012 workshop on Partial evaluation and program manipulation*, 43–52 (ACM, 2012).

[14] Gulwani, S. *et al.* Inductive programming meets the real world. *Communications of the ACM* (2015).

[15] Lake, B. M., Salakhutdinov, R. & Tenenbaum, J. B. Human-level concept learning through probabilistic program induction. *Science* **350**, 1332–1338 (2015).

[16] Rajalingham, R. *et al.* Large-scale, high-resolution comparison of the core visual object recognition behavior of humans, monkeys, and state-of-the-art deep artificial neural networks. *Journal of Neuroscience* **38**, 7255–7269 (2018).

[17] April, L. B., Bruce, K. & Galizio, M. The magic number 70 (plus or minus 20): variables determining performance in the rodent odor span task. *Learning and Motivation* **44**, 143–158 (2013).

[18] Vanschoren, J., van Rijn, J. N., Bischl, B. & Torgo, L. OpenML: networked science in machine learning. *ACM SIGKDD Explorations Newsletter* **15**, 49–60 (2014).

[19] Bellemare, M. G., Naddaf, Y., Veness, J. & Bowling, M. The arcade learning environment: An evaluation platform for general agents. *Journal of Artificial Intelligence Research* **47**, 253–279 (2013).

[20] Mnih, V. *et al.* Human-level control through deep reinforcement learning. *Nature* **518**, 529–533 (2015).

[21] Perez, D. *et al.* The 2014 general video game playing competition. *IEEE Transactions on Computational Intelligence and AI in Games* **8**, 229–243 (2015).

[22] Perez-Liebana, D. *et al.* General video game AI: A multitrack framework for evaluating agents, games, and content generation algorithms. *IEEE Transactions on Games* **11**, 195–214 (2019).

[23] Perez-Liebana, D., Samothrakis, S., Togelius, J., Schaul, T. & Lucas, S. M. General video game AI: Competition, challenges and opportunities. In *Thirtieth AAAI Conference on Artificial Intelligence* (2016).

[24] Damerius, L. A. *et al.* General cognitive abilities in orangutans (pongo abelii and pongo pygmaeus). *Intelligence* **74**, 3–11 (2018).

[25] Herrmann, E., Call, J., Hernández-Lloreda, M. V., Hare, B. & Tomasello, M. Humans have evolved specialized skills of social cognition: The cultural intelligence hypothesis. *Science* **317**, 1360–1366 (2007).

[26] Rust, J., Kosinski, M. & Stillwell, D. *Modern Psychometrics: The Science of Psychological Assessment* (Routledge, 2020), 4th edn.

[27] Carroll, J. B. Ability and task difficulty in cognitive psychology. *Educational Researcher* 11–21 (1981).

[28] Embretson, S. E. & Reise, S. P. *Item response theory for psychologists* (L. Erlbaum, 2000).

[29] De Ayala, R. J. *Theory and practice of item response theory* (Guilford Publications, 2009).

[30] Vale, C. D. & Weiss, D. J. A study of computer-administered stradaptive ability testing. Tech. Rep., Minnesota Univ. Minneapolis Dept. of Psychology (1975).

[31] Trabin, T. E. & Weiss, D. J. The person response curve: Fit of individuals to item response theory models. In *New horizons in testing*, 83–108 (Elsevier, 1983).

[32] Meijer, R. R. & Sijtsma, K. Methodology review: Evaluating person fit. *Applied Psychological Measurement* **25**, 107–135 (2001).

[33] Tatsuoka, K. K. & Tatsuoka, M. M. Spotting erroneous rules of operation by the individual consistency index. *Journal of Educational Measurement* **20**, 221–230 (1983).

[34] Kane, M. T. & Brennan, R. L. Agreement coefficients as indices of dependability for domain-referenced tests. *Applied Psychological Measurement* **4**, 105–126 (1980).

[35] Guttman, L. A basis for scaling qualitative data. *American sociological review* **9**, 139–150 (1944).

[36] Guttman, L. The basis for scalogram analysis. In Stouffer, S. A. *et al.* (eds.) *Measurement and prediction*, 60–90 (Princeton University Press, 1950).

[37] Mokken, R. J. & Lewis, C. A nonparametric approach to the analysis of dichotomous item responses. *Applied psychological measurement* **6**, 417–430 (1982).

[38] Sijtsma, K. & Molenaar, I. W. *Introduction to nonparametric item response theory*, vol. 5 (Sage, 2002).

[39] Lumsden, J. Person reliability. *Applied Psychological Measurement* **1**, 477–482 (1977).

[40] Ferrando, P. J. A general approach for assessing person fit and person reliability in typical-response measurement. *Applied Psychological Measurement* **38**, 166–183 (2014).

[41] Spearman, C. General Intelligence, Objectively Determined and Measured. *The American Journal of Psychology* **15**, 201–92 (1904).

[42] Spearman, C. *The abilities of man: Their nature and measurement* (Macmillan, New York, 1927).

[43] Jensen, A. R. *The g factor: The science of mental ability* (Westport, Praeger, 1998).

[44] Sternberg, R. J. *Handbook of intelligence* (Cambridge University Press, 2000).

[45] Guttman, L. & Guttman, R. A theory of behavioral generality and specificity during mild stress. *Systems Research and Behavioral Science* **21**, 469–477 (1976).

[46] Detterman, D. K. & Daniel, M. H. Correlations of mental tests with each other and with cognitive variables are highest for low IQ groups. *Intelligence* **13**, 349–359 (1989).

[47] Deary, I. J. *et al.* Intelligence and the differentiation hypothesis. *Intelligence* **23**, 105–132 (1996).

[48] Tucker-Drob, E. M. Differentiation of cognitive abilities across the life span. *Developmental psychology* **45**, 1097 (2009).

[49] Fogarty, G. J. & Stankov, L. Challenging the "law of diminishing returns". *Intelligence* **21**, 157–174 (1995).

[50] Hernández-Orallo, J. Is Spearman's law of diminishing returns (SLODR) meaningful for artificial agents? In *ECAI 2016 - 22nd European Conference on Artificial Intelligence*, 471–479 (2016).

[51] Woolley, A. W., Chabris, C. F., Pentland, A., Hashmi, N. & Malone, T. W. Evidence for a collective intelligence factor in the performance of human groups. *Science* **330**, 686–688 (2010).

[52] Woodley, M. A. & Bell, E. Is collective intelligence (mostly) the general factor of personality? a comment on Woolley, Chabris, Pentland, Hashmi and Malone (2010). *Intelligence* **39**, 79–81 (2011).

[53] Baggio, J. A. *et al.* The importance of cognitive diversity for sustaining the commons. *Nature communications* **10**, 1–11 (2019).

[54] Kuncheva, L. I. & Whitaker, C. J. Measures of diversity in classifier ensembles and their relationship with the ensemble accuracy. *Machine learning* **51**, 181–207 (2003).

[55] Kuncheva, L. I. *Combining pattern classifiers: methods and algorithms* (John Wiley & Sons, 2004).

[56] Bella, A., Ferri, C., Hernández-Orallo, J. & Ramírez-Quintana, M. J. On the effect of calibration in classifier combination. *Applied intelligence* **38**, 566–585 (2013).

[57] Fodor, J. A. *The modularity of mind: An essay on faculty psychology* (MIT press, 1983).

[58] Piaget, J. & Cook, M. *The origins of intelligence in children*, vol. 8 (International Universities Press New York, 1952).

[59] Jolly, A. Lemur social behavior and primate intelligence. *Science* **153**, 501–506 (1966).

[60] Byrne, R. & Whiten, A. *Machiavellian intelligence: social expertise and the evolution of intellect in monkeys, apes, and humans* (Oxford science publications, Clarendon Press, 1989).

[61] Whiten, A. & Byrne, R. W. *Machiavellian intelligence II: Extensions and evaluations*, vol. 2 (Cambridge University Press, 1997).

[62] Holekamp, K. E. Questioning the social intelligence hypothesis. *Trends in cognitive sciences* **11**, 65–69 (2007).

[63] Seed, A., Emery, N. & Clayton, N. Intelligence in corvids and apes: a case of convergent evolution? *Ethology* **115**, 401–420 (2009).

[64] Anderson, B. The g factor in non-human animals. In Gregory R. Bock, J. A. G. & Webb, K. (eds.) *The nature of intelligence*, vol. 233, 79–95 (John Wiley & Sons, 2000).

[65] Burkart, J. M., Schubiger, M. N. & van Schaik, C. P. The evolution of general intelligence. *Behavioral and Brain Sciences* **40** (2017).

[66] Poirier, M.-A., Kozlovsky, D. Y., Morand-Ferron, J. & Careau, V. How general is cognitive ability in non-human animals? a meta-analytical and multi-level reanalysis approach. *Proceedings of the Royal Society B* **287**, 20201853 (2020).

[67] Van Schaik, C. P., Isler, K. & Burkart, J. M. Explaining brain size variation: from social to cultural brain. *Trends in cognitive sciences* **16**, 277–284 (2012).

[68] Allman, J., McLaughlin, T. & Hakeem, A. Brain weight and life-span in primate species. *Proceedings of the National Academy of Sciences* **90**, 118–122 (1993).

[69] Lefebvre, L. Brains, innovations, tools and cultural transmission in birds, non-human primates, and fossil hominins. *Frontiers in human neuroscience* **7**, 245 (2013).

[70] Sol, D., Ducatez, S. & Sayol, F. Cognitive buffer hypothesis, the. *Encyclopedia of Evolutionary Psychological Science* 1–6 (2016).

[71] Del Giudice, M. & Crespi, B. J. Basic functional trade-offs in cognition: An integrative framework. *Cognition* **179**, 56–70 (2018).

[72] Woodley of Menie, M. A., Fernandes, H. B., te Nijenhuis, J., Peñaherrera-Aguirre, M. & Figueredo, A. J. General intelligence is a source of individual differences between species: Solving an anomaly. *Behavioral and Brain Sciences* **40** (2017).

[73] Shuker, D. M., Barrett, L., Dickins, T. E., Scott-Phillips, T. C. & Barton, R. A. General intelligence does not help us understand cognitive evolution. *Behavioral and Brain Sciences* **40** (2017).

[74] Newell, A., Shaw, J. C. & Simon, H. A. Report on a general problem-solving program. In *IFIP Congress*, 256–264 (1959).

[75] Ernst, G. W. & Newell, A. *GPS: A case study in generality and problem solving* (Academic Press, 1969).

[76] McCarthy, J. Generality in artificial intelligence. *Communications of the ACM* **30**, 1030–1035 (1987).

[77] Tenenbaum, J. B., Kemp, C., Griffiths, T. L. & Goodman, N. D. How to grow a mind: Statistics, structure, and abstraction. *Science* **331**, 1279–1285 (2011).

[78] Marcus, G. & Davis, E. *Rebooting AI: building artificial intelligence we can trust* (Vintage, 2019).

[79] Lecun, Y. The future is self-supervised. ICLR Invited Talk (2020).

[80] Silver, D. *et al.* Mastering chess and shogi by self-play with a general reinforcement learning algorithm. *arXiv preprint arXiv:1712.01815* (2017).

[81] Bowling, M., Burch, N., Johanson, M. & Tammelin, O. Heads-up limit hold'em poker is solved. *Science* **347**, 145–149 (2015).

[82] Brown, N. & Sandholm, T. Superhuman AI for multiplayer poker. *Science* **365**, 885–890 (2019).

[83] Schrittwieser, J. *et al.* Mastering atari, go, chess and shogi by planning with a learned model. *Nature* **588**, 604–609 (2020).

[84] Brown, T. B. *et al.* Language models are few-shot learners. *Advances in Neural Information Processing Systems 33 (NeurIPS 2020), arXiv preprint arXiv:2005.14165* (2020).

[85] Adams, S. *et al.* Mapping the landscape of human-level artificial general intelligence. *AI magazine* **33**, 25–42 (2012).

[86] Besold, T. R. & Schmid, U. Why generality is key to human-level artificial intelligence. *Advances in Cognitive Systems* 13–24 (2016).

[87] Solomonoff, R. J. A preliminary report on a general theory of inductive inference (1960). Report V-131, Zator Co., Cambridge, Ma. Feb 4, revision, Nov.

[88] Solomonoff, R. J. A formal theory of inductive inference. Part I. *Information and control* **7**, 1–22 (1964).

[89] Wolpert, D. H. The lack of a priori distinctions between learning algorithms. *Neural Computation* **8**, 1341–1390 (1996).

[90] Hutter, M. *Universal Artificial Intelligence: Sequential Decisions based on Algorithmic Probability* (Springer, 2005).

[91] Hernández-Orallo, J. *The Measure of All Minds: Evaluating Natural and Artificial Intelligence* (Cambridge University Press, 2017).

[92] Igel, C. & Toussaint, M. A no-free-lunch theorem for non-uniform distributions of target functions. *Journal of Mathematical Modelling and Algorithms* **3**, 313–322 (2005).

[93] Wolpert, D. H. & Macready, W. G. No free lunch theorems for search. Tech. Rep., SFI-TR-95-02-010 (Santa Fe Institute) (1995).

[94] Wolpert, D. H. What the no free lunch theorems really mean; how to improve search algorithms. Tech. Rep., Santa fe Institute Working Paper (2012).

[95] Edmonds, B. The social embedding of intelligence. In Epstein, R., Roberts, G. & Beber, G. (eds.) *Parsing the Turing Test*, 211–235 (Springer, 2009).

[96] Chollet, F. On the measure of intelligence. arXiv preprint arXiv:191101547 (2019).

[97] Hernández-Orallo, J. On the computational measurement of intelligence factors. In Meystel, A. M. & Messina, E. R. (eds.) *Measuring the performance and intelligence of systems: proceedings of the 2000 PerMIS Workshop, August 14–16, 2000*, 72–79 (National Institute of Standards and Technology (NIST) Special Publication 970, Gaithersburg, MD, U.S.A., 2000).

[98] Insa-Cabrera, J., Hernández-Orallo, J., Dowe, D. L., Espana, S. & Hernández-Lloreda, M. V. The anynt project intelligence test: Lambda-one. In *AISB/IACAP 2012 Symposium "Revisiting Turing and his Test*, 20–27 (2012).

[99] Legg, S. & Hutter, M. Universal intelligence: A definition of machine intelligence. *Minds and Machines* **17**, 391–444 (2007).

[100] Hernández-Orallo, J. A note about the generalisation of the c-tests. *arXiv preprint, arXiv:1412.8529* (2015).

[101] Hernández-Orallo, J. C-tests revisited: Back and forth with complexity. In Bieger, J., Goertzel, B. & Potapov, A. (eds.) *Artificial General Intelligence - 8th International Conference, AGI 2015, Berlin, Germany, July 22-25, 2015, Proceedings*, 272–282 (Springer, 2015).

[102] Hibbard, B. Bias and no free lunch in formal measures of intelligence. *Journal of Artificial General Intelligence* **1**, 54–61 (2009).

[103] Hernández-Orallo, J. & Dowe, D. L. Measuring universal intelligence: Towards an anytime intelligence test. *Artificial Intelligence* **174**, 1508 – 1539 (2010).

[104] Hernández-Orallo, J. Stochastic tasks: Difficulty and Levin search. In Bieger, J., Goertzel, B. & Potapov, A. (eds.) *Artificial General Intelligence - 8th International Conference, AGI 2015, Berlin, Germany, July 22-25, 2015, Proceedings*, 90–100 (Springer, 2015).

[105] Hernández-Orallo, J. Universal psychometrics tasks: difficulty, composition and decomposition. *arXiv preprint arXiv:1503.07587* (2015).

[106] van Horik, J. O. & Lea, S. E. Disentangling learning from knowing: Does associative learning ability underlie performances on cognitive test batteries? *Behavioral and Brain Sciences* **40** (2017).

[107] Dowe, D. L. & Hernandez-Orallo, J. IQ tests are not for machines, yet. *Intelligence* **40**, 77–81 (2012).

[108] Besold, T., Hernández-Orallo, J. & Schmid, U. Can machine intelligence be measured in the same way as human intelligence? *KI-Künstliche Intelligenz* **29**, 291–297 (2015).

[109] Hernández-Orallo, J., Martínez-Plumed, F., Schmid, U., Siebers, M. & Dowe, D. L. Computer models solving intelligence test problems: Progress and implications. *Artificial Intelligence* **230**, 74–107 (2016).

[110] Martínez-Plumed, F., Ferri, C., Hernández-Orallo, J. & Ramírez-Quintana, M. J. A computational analysis of general intelligence tests for evaluating cognitive development. *Cognitive Systems Research* **43**, 100–118 (2017).

[111] Li, M. & Vitányi, P. *An introduction to Kolmogorov complexity and its applications (3rd ed.)* (Springer-Verlag, 2008).

[112] Hernández-Orallo, J., Dowe, D. L. & Hernández-Lloreda, M. V. Measuring cognitive abilities of machines, humans and non-human animals in a unified way: towards universal psychometrics. *Technical Report 2012/267, Faculty of Information Technology, Clayton School of I.T., Monash University, Australia* (March 2012).

[113] Hernández-Orallo, J., Dowe, D. L. & Hernández-Lloreda, M. V. Universal psychometrics: Measuring cognitive abilities in the machine kingdom. *Cognitive Systems Research* **27**, 50–74 (2014).

[114] Dowe, D. L. & Hernández-Orallo, J. How universal can an intelligence test be? *Adaptive Behavior* **22**, 51–69 (2014).

[115] Hernández-Orallo, J. A (hopefully) non-biased universal environment class for measuring intelligence of biological and artificial systems. In et al., M. H. (ed.) *Artificial General Intelligence, 3rd Intl Conf*, 182–183 (Atlantis Press, 2010).

[116] Insa-Cabrera, J., Dowe, D. L., España-Cubillo, S., Hernández-Lloreda, M. V. & Hernández-Orallo, J. Comparing humans and AI agents. In *International Conference on Artificial General Intelligence*, 122–132 (Springer, 2011).

[117] Insa-Cabrera, J., Dowe, D. L. & Hernández-Orallo, J. Evaluating a reinforcement learning algorithm with a general intelligence test. In Lozano, J., Gamez, J. & Moreno, J. (eds.) *Current Topics in Artificial Intelligence. CAEPIA 2011* (LNAI Series 7023, Springer, 2011).

[118] Martínez-Plumed, F., Prudêncio, R. B. C., Martínez-Usó, A. & Hernández-Orallo, J. Making sense of item response theory in machine learning. In *ECAI 2016 - 22nd European Conference on Artificial Intelligence, Best Paper Award*, 1140–1148 (2016).

[119] Martínez-Plumed, F. & Hernández-Orallo, J. AI results for the Atari 2600 games: difficulty and discrimination using IRT. *EGPAI, Evaluating General-Purpose Artificial Intelligence* (2016).

[120] Martínez-Plumed, F. *et al.* Accounting for the neglected dimensions of ai progress. *arXiv preprint arXiv:1806.00610* (2018).

[121] Prudêncio, R. B., Hernández-Orallo, J. & Martınez-Usó, A. Analysis of instance hardness in machine learning using item response theory. In *Second International Workshop on Learning over Multiple Contexts in ECML 2015. Porto, Portugal, 11 September 2015*, vol. 1 (2015).

[122] Hofstadter, D. R. *Gödel, Escher, Bach* (Vintage Books New York, 1980).

[123] Hernández-Orallo, J. Evaluation in artificial intelligence: from task-oriented to ability-oriented measurement. *Artificial Intelligence Review* **48**, 397–447 (2017).

[124] Genesereth, M., Love, N. & Pell, B. General game playing: Overview of the AAAI competition. *AI Magazine* **26**, 62 (2005).

[125] Genesereth, M. & Thielscher, M. General game playing. *Synthesis Lectures on Artificial Intelligence and Machine Learning* **8**, 1–229 (2014).

[126] Whiteson, S., Tanner, B. & White, A. The Reinforcement Learning Competitions. *The AI magazine* **31**, 81–94 (2010).

[127] Dimitrakakis, C., Li, G. & Tziortziotis, N. The reinforcement learning competition 2014. *AI Magazine* **35**, 61–65 (2014).

[128] McDermott, J. *et al.* Genetic programming needs better benchmarks. In *Proceedings of the 14th international conference on genetic and evolutionary computation conference*, 791–798 (ACM, 2012).

[129] White, D. R. *et al.* Better GP benchmarks: Community survey results and proposals. *Genetic Programming and Evolvable Machines* **14**, 3–29 (2013).

[130] Schaul, T. An extensible description language for video games. *Computational Intelligence and AI in Games, IEEE Transactions on* **6**, 325–331 (2014).

[131] Machado, M. C. *et al.* Revisiting the arcade learning environment: Evaluation protocols and open problems for general agents. arXiv preprint arXiv:1709.06009 (2017).

[132] Castelvecchi, D. Tech giants open virtual worlds to bevy of AI programs. *Nature* **540**, 323–324 (2016).

[133] Hernández-Orallo, J. *et al.* A new AI evaluation cosmos: Ready to play the game? *AI Magazine* **38** (2017).

[134] Crosby, M. *et al.* Translating from animal cognition to AI. NeurIPS Workshop on Biological and Artificial Reinforcement learning (2019).

[135] Puigdomènech-Badia, A. *et al.* Agent57: Outperforming the Atari human benchmark. *arXiv preprint arXiv:2003.13350* (2020).

[136] Crosby, M. *et al.* The animal-AI testbed and competition. *Proceedings of Machine Learning Research* 164–176 (2020).

[137] Bontrager, P., Khalifa, A., Mendes, A. & Togelius, J. Matching games and algorithms for general video game playing. In *Twelfth Artificial Intelligence and Interactive Digital Entertainment Conference*, 122–128 (2016).

[138] Mendes, A., Nealen, A. & Togelius, J. Hyperheuristic general video game playing. In *Proceedings of Computational Intelligence and Games (CIG). IEEE* (2016).

[139] Elo, A. E. *The rating of chessplayers, past and present*, vol. 3 (Batsford London, 1978).

[140] Aziz, H. *et al.* Possible and necessary winners of partial tournaments. *Journal of Artificial Intelligence Research* **54**, 493–534 (2015).

[141] Balduzzi, D., Tuyls, K., Perolat, J. & Graepel, T. Re-evaluating evaluation. In *Advances in Neural Information Processing Systems*, 3268–3279 (2018).

[142] Czarnecki, W. M. *et al.* Real world games look like spinning tops. *arXiv preprint arXiv:2004.09468* (2020).

[143] Tuyls, K. *et al.* Bounds and dynamics for empirical game theoretic analysis. *Autonomous Agents and Multi-Agent Systems* **34**, 7 (2020).

[144] Hernández-Orallo, J. & Dowe, D. L. On potential cognitive abilities in the machine kingdom. *Minds and Machines* **23**, 179–210 (2013).

[145] Martínez-Plumed, F., Ferri, C., Hernández-Orallo, J. & Ramírez-Quintana, M. J. Knowledge acquisition with forgetting: an incremental and developmental setting. *Adaptive Behavior* **23**, 283–299 (2015).

[146] Silver, D. *et al.* Mastering the game of go with deep neural networks and tree search. *Nature* **529**, 484–489 (2016).

[147] Silver, D. *et al.* Mastering the game of go without human knowledge. *Nature* **550**, 354–359 (2017).

[148] Hendrycks, D. *et al.* Measuring massive multitask language understanding. *arXiv preprint arXiv:2009.03300* (2020).

[149] Tamkin, A., Brundage, M., Clark, J. & Ganguli, D. Understanding the capabilities, limitations, and societal impact of large language models. *arXiv preprint arXiv:2102.02503* (2021).

[150] Vitányi, P. & Li, M. On prediction by data compression. In *Machine Learning: ECML-97*, 14–30 (Springer, 1997).

[151] Lathrop, J. I. Compression depth and genetic programs. *Genetic Programming* 370–379 (1997).

[152] Dowe, D. L., Hernández-Orallo, J. & Das, P. K. Compression and intelligence: social environments and communication. In Schmidhuber, J., Thórisson, K. & Looks, M. (eds.) *Artificial General Intelligence*, vol. 6830, 204–211 (LNAI series, Springer, 2011).

[153] Kinnear Jr, K. E. Generality and difficulty in genetic programming: Evolving a sort. In *ICGA*, 287–294 (1993).

[154] Tenenbaum, J. B. & Griffiths, T. L. Generalization, similarity, and Bayesian inference. *Behavioral and brain sciences* **24**, 629 (2001).

[155] Balla, M., Lucas, S. M. & Perez-Liebana, D. Evaluating generalisation in general video game playing. In *2020 IEEE Conference on Games (CoG)*, 423–430 (2020).

[156] Owen, D. B. A table of normal integrals. *Communications in Statistics-Simulation and Computation* **9**, 389–419 (1980).

[157] Hernandez-Orallo, J. AI generality and Spearman's law of diminishing returns. *Journal of Artificial Intelligence Research* **64**, 529–562 (2019).

[158] Kapturowski, S., Ostrovski, G., Quan, J., Munos, R. & Dabney, W. Recurrent experience replay in distributed reinforcement learning (2018).

[159] Martínez-Plumed, F. & Hernández-Orallo, J. Dual indicators to analyse AI benchmarks: Difficulty, discrimination, ability and generality. *IEEE Transactions on Games* **12**, 121–131 (2020).

[160] Osborne, J. W. & Costello, A. B. Sample size and subject to item ratio in principal components analysis. *Practical assessment, research & evaluation* **9**, 8 (2004).

[161] Morizot, J., Ainsworth, A. T. & Reise, S. P. Toward modern psychometrics. *Handbook of research methods in personality psychology* 407 (2009).

[162] Hernández-Orallo, J., Dowe, D. L., España-Cubillo, S., Hernández-Lloreda, M. V. & Insa-Cabrera, J. On more realistic environment distributions for defining, evaluating and developing intelligence. In Schmidhuber, J., Thórisson, K. & Looks, M. (eds.) *Artificial General Intelligence*, vol. 6830, 82–91 (LNAI, Springer, 2011).

[163] Insa-Cabrera, J., Benacloch-Ayuso, J.-L. & Hernández-Orallo, J. On measuring social intelligence: Experiments on competition and cooperation. In *International Conference on Artificial General Intelligence*, 126–135 (Springer, 2012).

[164] Insa-Cabrera, J. & Hernández-Orallo, J. Instrumental properties of social testbeds. In *International Conference on Artificial General Intelligence*, 101–110 (Springer, 2015).

[165] R Core Team. R: A language and environment for statistical computing (2014). URL http://www.R-project.org/.
